# ATM inhibitor KU60019 synergistically sensitizes lung cancer cells to topoisomerase II poisons by multiple mechanisms

**DOI:** 10.1038/s41598-023-28185-z

**Published:** 2023-01-17

**Authors:** Jianfeng Shu, Xiaofang Wang, Xuejie Yang, Guofang Zhao

**Affiliations:** 1 HwaMei Hospital, University of Chinese Academy of Sciences, 41 Xibei Road, Ningbo, 315010 Zhejiang China; 2 Ningbo Institute of Life and Health Industry, University of Chinese Academy of Sciences, Ningbo, 315000 Zhejiang China

**Keywords:** Biochemistry, Cancer, Cell biology, Molecular biology

## Abstract

Type II topoisomerases (TOP2) poisons represent one class of the most successful and widely prescribed chemotherapeutics, which is frontline therapy for a myriad of systemic cancers and solid tumors, including lymphomas, leukemias, and lung cancer. Despite this, treatment with this class of drugs induces unwanted side effects (including cardiovascular morbidity and secondary malignancies). Additionally, the emergence of drug resistance also greatly compromises the clinical use of these drugs. To enhance therapeutic efficiency while lowering unwanted side effects, new insights into effective combination therapy are required. In this study we found that KU60019, a novel, and highly specific ATM kinase inhibitor interferes with the association of ATM with TOP2β and stabilizes TOP2β-DNA cleavage complex, thereby impairing the repair of TOP2 poison-induced DSBs and contributes to genome stability, leading to accelerated cell death. In H1299 as well as in A549 lung cancer cell lines, biologically, KU60019 combined with VP-16 (one of the TOP2 poisons) synergistically suppressed the growth of cells and survival and triggered a much higher apoptosis rate. In summary, we provide a proof-of-concept strategy that ATM inhibitors combined with TOP2 poison would synergistically suppresses lung cancer cell survival as well as reduce DNA damage responses, thus may lowering the possibility of cardiotoxicity and secondary malignancy linked to therapy.

## Introduction

Type II topoisomerases (TOP2) are ubiquitous enzymes that address the topological issues that arise from double-stranded DNA by introducing double-strand breaks (DSBs) into the first segment of DNA that is bridged by an enzyme, in which dimeric enzyme’s each monomer covalently binds to the DSBs ends utilizing a 5’-phosphotyrosyl link and the second segment of DNA is facilitated to ‘‘pass” through the DNA gate with an enzyme bridge^1,2^. Finally, the break is re-ligated in situ^2^. Besides, failure to complete re-ligation can result in persistent TOP2-DNA covalent complexes (TOP2cc)^3,4^. In fact, TOP2 poisons that impede the re-ligation phase of the reaction cycle have been used in cancer therapy to take advantage of this TOP2 characteristic, resulting in an accumulation of TOP2-DNA adducts^4,5^. Elongating polymerases can be stalled by the TOP2-DNA adducts, As a result, replication and transcription are inhibited, potentially resulting in cell death^6^. To survive the stress on TOP2cc, cancer cells have evolved a myriad of DNA repair enzymes and cellular processes to eliminate these covalent TOP2-DNA complexes, resulting in protein-free DSBs, facilitating repair of the unique DSBs by standard DNA repair pathways^7,8^. If mis-repaired, DSBs often cause chromosome translocation and genomic instability^9^. A high prevalence of cardiotoxicity and secondary malignancies is linked to TOP2-based medications based on such mechanisms^10,11^. Meanwhile, TOP2 degradation can also lead to the decrease of cytotoxic TOP2cc levels in tumor cells, leading to the emergence of drug resistance^12^. Despite this, TOP2 poison remains a crucial factor in cancer treatment. To enhance therapeutic efficiency while lowering unwanted side effects, new insights into effective combination therapy are required.

To eliminate TOP2 from the DNA 5′-end, the DNA repair enzyme called tyrosyl-DNA phosphodiesterase 2 (TDP2) catalyzes the hydrolysis of the covalent bond between the TOP2 catalytic tyrosine hydroxyl groups and the DNA phosphate^8,13^. By using a specific oligonucleotide to study the irreversible TOP2 cleavage complexes, Rui Gao and colleagues demonstrate that native TOP2 cleavage complexes first need to be proteolyzed before they can be processed by TDP2^14^. Consistently, prior research has demonstrated that the TOP2 poison etoposide (VP-16) induces degradation of both TOP2α and TOP2β (two TOP2 isoforms) in a manner that is dependent on ubiquitin–proteasome, with TOP2β being preferentially degraded over TOP2α^15^. Therefore, the formation of DSBs and DNA sequence rearrangements are primarily caused by TOP2β degradation^16^. Mechanistically, we reported recently that upon the TOP2 poison stimuli, ATM attaches to and phosphorylates TOP2β, thereby facilitating the ubiquitination and degradation of TOP2β through SCF^β-TrCP^^17^. Thus, we hypothesized that the combination of small molecule inhibitors targeting ATM with TOP2-targeting medications may not only synergistically suppresses lung cancer cell survival but also attenuate the DNA damage response, thus lowering the possibility of cardiotoxicity and secondary malignancy caused by therapy.

A serine/threonine kinase that belongs to the phosphatidylinositol 3-kinase (PI3K)-related protein kinase (PIKK) family, ataxia telangiectasia mutated (ATM), is mutated in the uncommon human disease ataxia-telangiectasia (A-T). Initial studies on ATM focused on how it affected DNA damage response (DDR)^18^. In response to DSBs, ATM recruits and activates DNA checkpoint response and promotes either HR or NHEJ repair of broken chromosomes^18,19^. Importantly, it has been reported that IR and DNA-damaging agents are effectively enhanced by ATM inhibition, even if it is temporary, while it is not problematic for normal cells^20^. Accordingly, ATM inhibition seems a promising approach for anticancer chemo-sensitization, as well as for overcoming drug resistance in tumors.

Etoposide (VP-16) represents one of the most successful and widely prescribed TOP2 poisons, which is used as first-line therapy for a variety of solid tumors and systemic cancers, including lymphomas, leukemia, and lung cancer^1^. Herein, we mechanistically and functionally demonstrate the therapeutic potential of VP-16 in combination with ATM inhibitors for the eradication of lung cancer cells. A novel, second-generation ATM kinase inhibitor, KU60019, demonstrated high selectivity and limited toxicity against healthy cells, for which the preclinical studies have been validated^21^, was selected as the combination therapy for VP-16 to validate our hypothesis. In this work, VP-16 alone or in conjunction with KU60019 was applied to cell lines H1299 and A549. The detection of TOP2βcc levels, as well as cell proliferation, survival, and apoptosis, demonstrated the synergistic effects. Authentically, a viable lung cancer-targeted therapeutic approach is offered by the combinatorial regimen, which in fact inhibits the ATM pathway to improve the tumor suppressive potential of VP-16.

## Result

### ATM signaling pathway is activated by VP-16 stimuli

**Figure 1:**
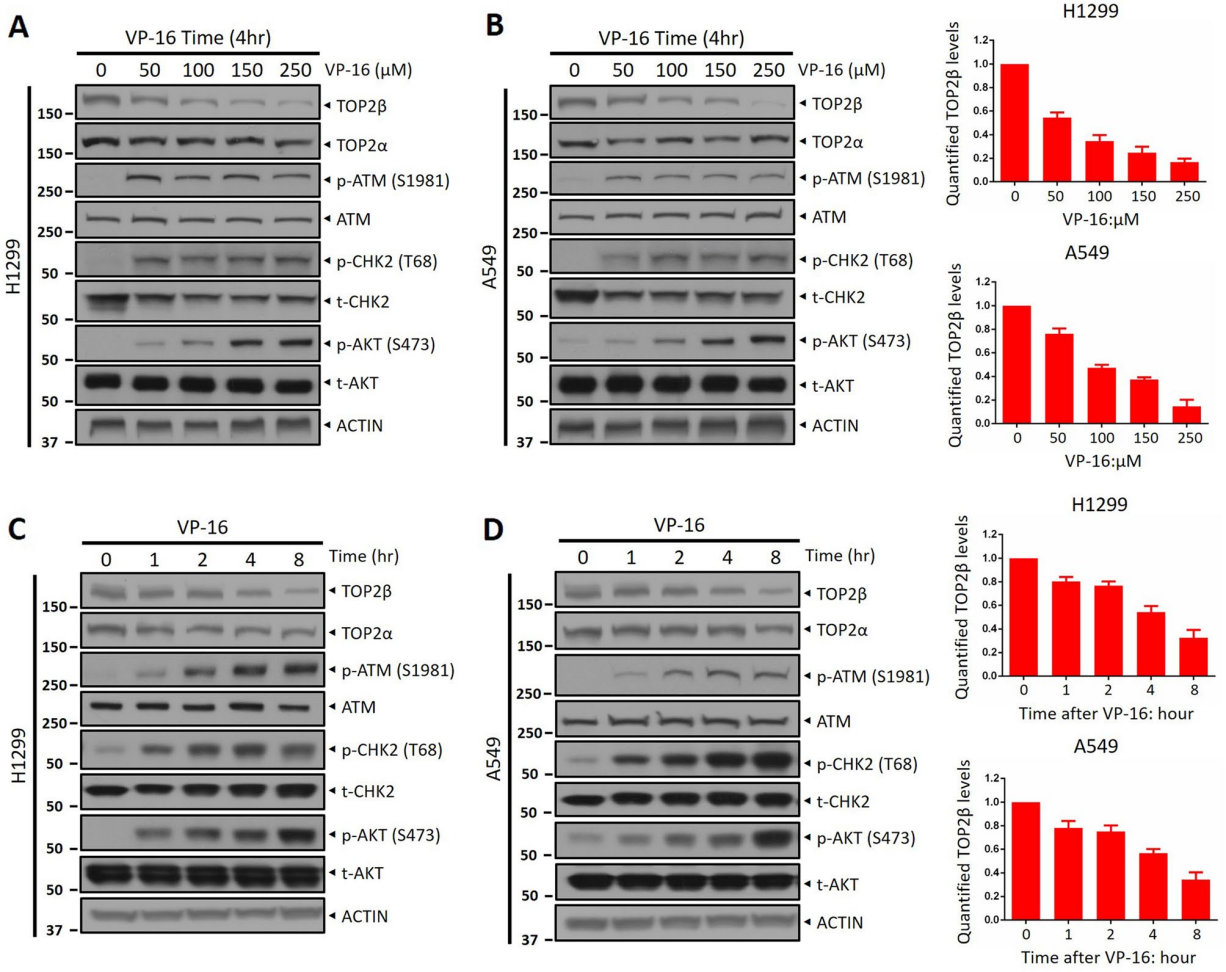
ATM signaling pathway is activated by VP-16 stimuli. (**A**, **B**) H1299 and A549 cells were incubated with various concentrations of VP-16 (0, 50, 100, and 250 μM) for 4 h, then, cells were collected, and subjected to immunoblotting (IB) with the indicated antibodies (Abs). ACTIN was used as a loading control. (**C**, **D**) H1299 and A549 cells were treated with VP-16 (100 μM) for the indicated time periods (0, 1, 2, 4, and 8 h), followed by western blotting using the indicated Abs. All experiments were independently repeated three times. Densitometry quantification was performed with Image J, and the decay curves are shown. According to the molecular weight, the nitrocellulose membrane was cut prior to hybridization with antibodies and the original blots are presented in Supplementary Information. Molecular weight markers are noted to the left of blot figure.

We have previously found that upon the TOP2 poison stimuli, ATM attaches to and phosphorylates TOP2β, thereby facilitating the ubiquitination and degradation of TOP2β by SCF^β-TrCP^^17^. Besides, accumulating evidence has shown that ATM activation is involved in cancer cell resistance to chemotherapy. To confirm that the ATM signaling is associated with VP-16 resistance, cell lines H1299 and A549 from human lung cancer were treated with VP-16 at various concentrations following the examination of ATM activation. Indeed, VP-16 does stimulate the ATM signaling pathway, as evidenced by elevated ATM phosphorylation on S1981 and elevated phosphorylation of AKT and CHK2 at S473 and T62 position, two effector kinases downstream of ATM^22^, after VP-16 treatment in a manner that is dependent on dose (Fig. 1A, B). Consistent with previous reports, VP-16 indeed led to a dramatic degradation of TOP2β with negligible influence on TOP2α levels (Fig. 1A, B). Similarly, ATM was also significantly activated by VP-16 over time and the phosphorylation level of ATM was negatively correlated with the protein level of TOP2β (Fig. 1C, D). Collectively, these results suggest an influence of ATM on the cancer cell killing of VP-16.

### KU60019, an ATM inhibitor causes a dose and time-dependent accumulation of TOP2β

**Figure 2:**
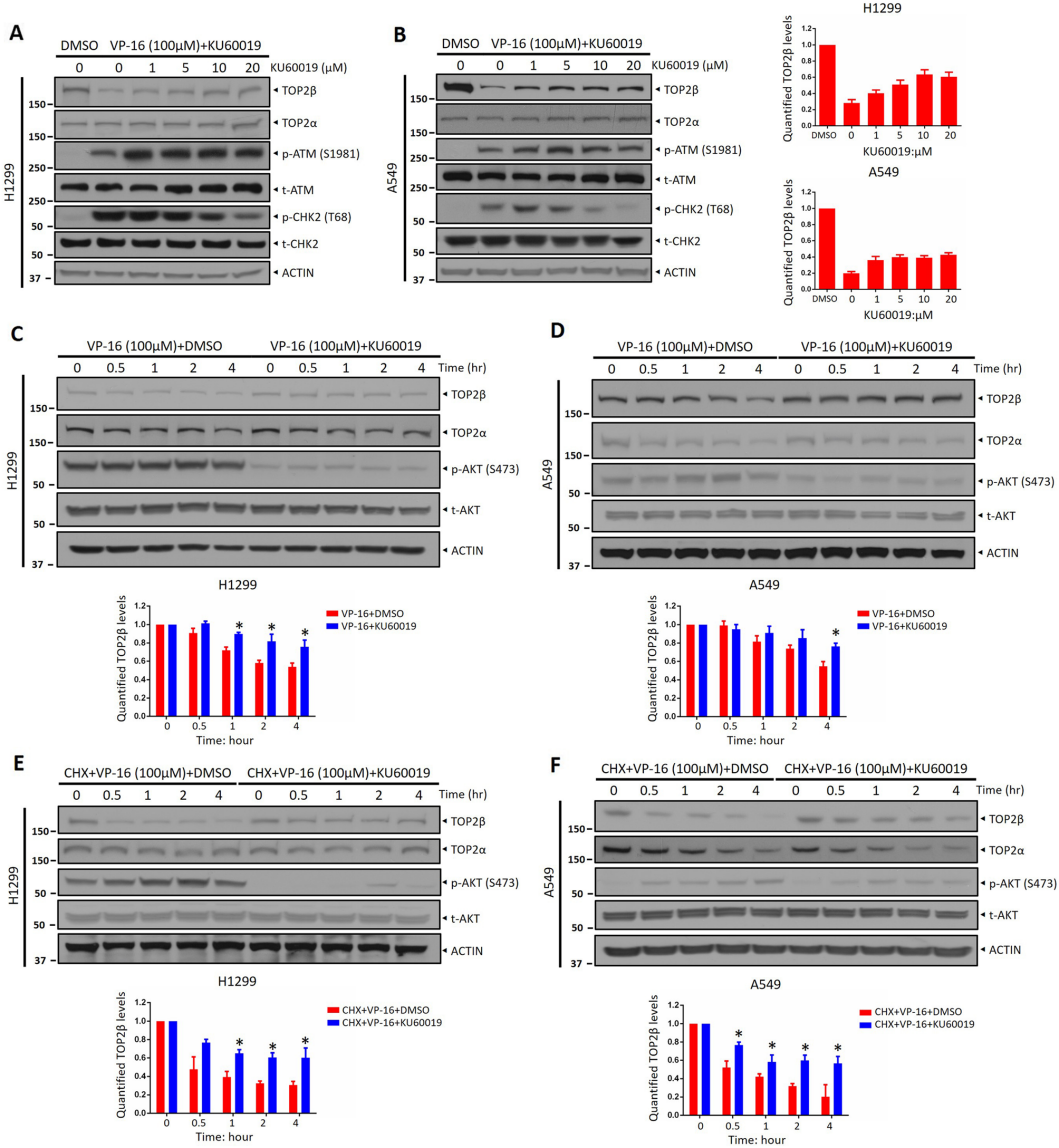
KU60019, an ATM inhibitor causes a dose and time-dependent accumulation of TOP2β. (**A**, **B**) H1299 and A549 cells were treated for 2 h with vehicle or VP-16 (100 μM) and varying doses of KU60019 (0, 1, 5, 10, and 20 μM) and then IB with the relevant antibodies. (**C**, **D**) H1299 and A549 cells were pretreated with DMSO or KU60019 (5 μM) for 1 h, followed by treatment with VP-16 for various time periods, and then, IB was undertaken with the indicated Abs. (**E**, **F**) H1299 and A549 cells following treatment with CHX and VP-16 or with CHX, VP-16, and KU60019 (5 μM) for the respective times, cells were collected and IB with the relevant antibodies. All experiments were independently repeated three times. Densitometry quantification was performed with Image J, and the decay curves are shown. According to the molecular weight, the nitrocellulose membrane was cut prior to hybridization with antibodies and the original blots are presented in Supplementary Information. Molecular weight markers are noted to the left of blot figure.

Given that the preclinical studies have validated KU60019 as a highly selective and low-toxic ATM kinase inhibitor with limited toxicity against healthy cells^21^. We then chose KU60019 for the further combination study. As it is known that ATM is associated with the degradation of TOP2β, and induces tumor cells to exhibit resistance to VP-16. We next determined whether the KU60019 could restrain the degradation of TOP2β in response to VP-16 stimuli by inhibition of ATM kinase activity. First, we treated H1299 and A549 cells for 2 h with numerous concentrations of KU60019 alone or in conjunction with VP-16, followed by an immunoblotting assay to evaluate ATM signaling and TOP2β levels. The ATM signaling pathway is inhibited by KU60019, as shown in Fig. 2A and B, as evidenced by decreased CHK2 phosphorylation on T68 after KU60019 therapy in a manner that is dependent on dose. Moreover, in H1299 and A549 cells, KU60019 led to a dose-dependent accumulation of TOP2β (Fig. 2A, B). Consistently, we found that ATM inhibitor KU60019 caused TOP2β protein accumulation in a manner that is dependent on time with minimal influence on TOP2α (Fig. 2C, D). In addition, by blocking new protein synthesis with cycloheximide (CHX), we found in tested cells that KU60019 treatment substantially prolonged the half-life of endogenous TOP2β (Fig. 2E, F). Collectively, these data suggested that KU60019 can block the degradation of TOP2β.

### KU60019 interferes with the association of ATM with TOP2β and blocks TOP2β ubiquitination

**Figure 3:**
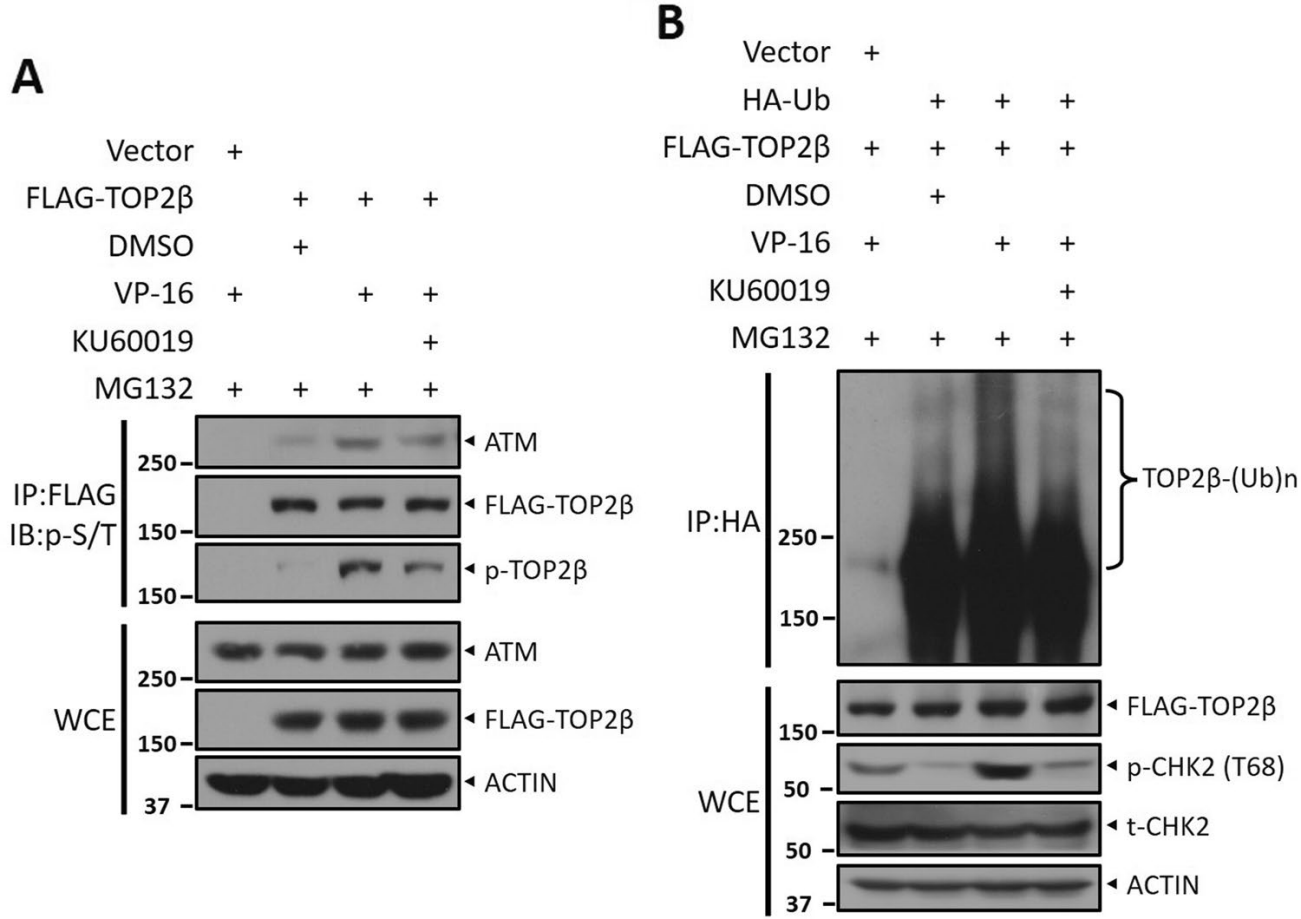
KU60019 interferes with the association of ATM with TOP2β and blocks TOP2β ubiquitination. (**A**, **B**) HEK293 cells were transfected with Flag-TOP2β for 48 h, then treated with KU60019 or vehicle combined with VP-16 and MG132 (20 μM) for 5 h, followed by IP with Flag beads and IB with the relevant antibodies. (**C**, **D**) HEK293 cells transfected with the specified plasmids were treated with VP-16 and MG132 (20 μM) or in association with KU60019 (5 μM) for 5 h, and then IP was performed with anti-HA beads, and direct IB was performed with the selected antibodies. All experiments were independently repeated three times. According to the molecular weight, the nitrocellulose membrane was cut prior to hybridization with antibodies and the original blots are presented in Supplementary Information. Molecular weight markers are noted to the left of blot figure.

Mechanistically, it is now known that ATM attaches to and phosphorylates TOP2β for targeted TOP2β ubiquitination. Next, we explore whether KU60019 could block ATM activity by interfering with binding to TOP2β and thereby whether KU60019 could prevent TOP2β from being ubiquitinated. We overexpressed Flag- TOP2β in HEK293 cells and found that treatment with KU60019 resulted in the dissociation of endogenous ATM and Flag-TOP2β by IP-based pulldown assay (Fig. 3A). Consistently, VP-16-induced polyubiquitination of TOP2β was similarly significantly suppressed by KU60019 treatment (Fig. 3B). Together, these results show that KU60019 interferes with the association of ATM with TOP2β and block TOP2β ubiquitination.

### KU60019 increases the level of the TOP2β-DNA cleavage complex and impairs the repair of VP-16-induced DSBs

**Figure 4:**
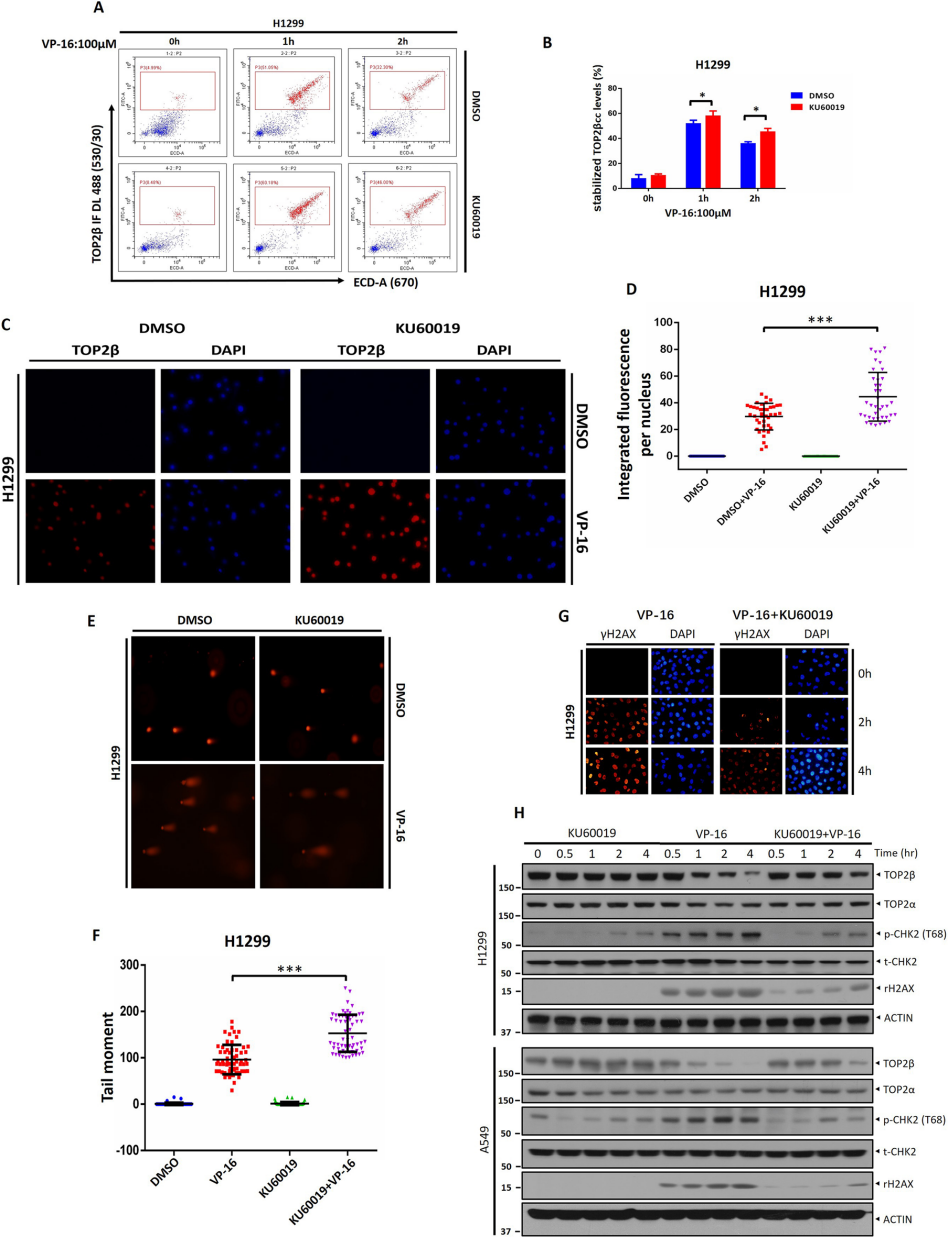
KU60019 increases the level of the TOP2β-DNA cleavage complex and impairs the repair of VP-16-induced DSBs. (**A**, **B**) H1299 cells were treated with VP-16 alone or in conjunction with KU60019 for the appropriate times, and then the TOP2cc levels were determined by FACS. (**C**, **D**) Two hours were spent treating H1299 cells with VP-16 or VP-16 in conjunction with KU60019 (5 μM). The cells were then collected for the TARDIS assay. (**E**, **F**) H1299 cells were pretreated with KU60019 (5 μM) for 1 h and then cotreated with VP-16 for an additional 1 h. Cells were then harvested for the neutral comet assay. Representative images are shown, and the data are presented as the mean ± S.D. from three independent experiments; ***p < 0.001. (**G**) Cells treated with VP-16 alone or in conjunction with KU60019 for the indicated time periods were harvested at the indicated time points for immunofluorescence. (**H**) H1299 and A549 cells were treated with KU60019 or VP-16 alone or in conjunction with IB with the indicated Abs. All experiments were independently repeated three times. According to the molecular weight, the nitrocellulose membrane was cut prior to hybridization with antibodies and the original blots are presented in Supplementary Information. Molecular weight markers are noted to the left of blot figure.

Previous studies have shown that TOP2 poisons impede the TOP2 reaction cycle’s re-ligation phase, causing an accumulation of cytotoxic TOP2 protein-DNA covalent complex (TOP2cc). To further confirm that KU60019 abrogates TOP2β degradation, thereby resulting in the accumulation of TOP2cc. A flow cytometry-based method (TOP2cc-flow cytometry assay) exploited by Marcelo de Campos Nebel et al*.* was carried out to assess the TOP2β-DNA cleavage complex levels^23^. As shown in Fig. 4A, B, VP-16 stimuli induce TOP2β to form cleavage complexes with DNA, while the inactivation of ATM by KU60019 induced a time-dependent elevation in levels of TOP2βcc. Meanwhile, the levels of TOP2βcc were also assessed employing the TARDIS assay. To detect drug-stabilized TOP2βcc in individual cells, H1299 cells were treated with VP-16 alone or in conjunction with KU60019 for two hours, and cells were subsequently embedded in agarose on microscope slides, following lysing to eliminate nuclear proteins. This procedure results in the capture of each cell's DNA in agarose, together with any drug-stabilized cleavable complexes linked to it. A primary anti-TOP2 antibody and FITC-conjugated second antibody were then used to detect covalently bound TOP2 in situ by immunofluorescence. The results showed that untreated cells showed no immunofluorescence associated with DNA, while VP-16-treated cells showed readily detectable immunofluorescence, and the immunofluorescence intensity was increased by KU60019 treatment (Fig. 4C, D), suggesting that the levels of remaining stabilized TOP2βcc were increased.

It has been reported that DNA damage signals are triggered by the degradation of TOP2 cleavage complexes, which can convert concealed DNA strand breaks into concealed DNA strand breaks, thus facilitating the repair of the unique DSBs by standard DNA repair pathways. We measured the amount of DSBs utilizing a neutral comet assay to ascertain if the VP-16-induced DNA damage response is physiologically mediated by ATM-driven TOP2 degradation. In line with expectations, VP-16 treatment substantially enhanced the comet tail moment, signifying that DSBs were induced (Fig. 4E, F). Co-treatment with KU60019 caused a significant increase in DSBs compared with the treatment of VP-16 alone (Fig. 4E, F). According to this finding, KU60019 stabilizes TOP2β-DNA covalent complexes and hides DSBS by restraining TOP2β degradation. Consequently, the neutral comet assay shows an increase in DSBs because of an impaired ability to detect damaged DNA to initiate DNA damage response, followed by repair.

Next, to further support this notion, we looked at the formation of foci and the levels of γH2AX. It is not surprising that VP-16 led to the formation of foci and elevated the level of γH2AX in H1299 cells as predicted (Fig. 4G, H). Distinctly, the co-treatment of KU60019, which inhibited TOP2β degradation, significantly reduced the formation of foci and levels of γH2AX (Fig. 4G, H). Likewise, TOP2β protein levels were also observed to accumulate over time in the presence of KU60019 (Fig. 4G, H). In conclusion, our results clearly demonstrate that KU60019 restrains TOP2β degradation induced by VP-16 stimuli, thereby blocking the conversion of TOP2cc into true DSBs and impairing the repair of VP-16-induced DSBs.

### KU60019 combined with VP-16 suppressed the survival of lung cancer cells

**Figure 5:**
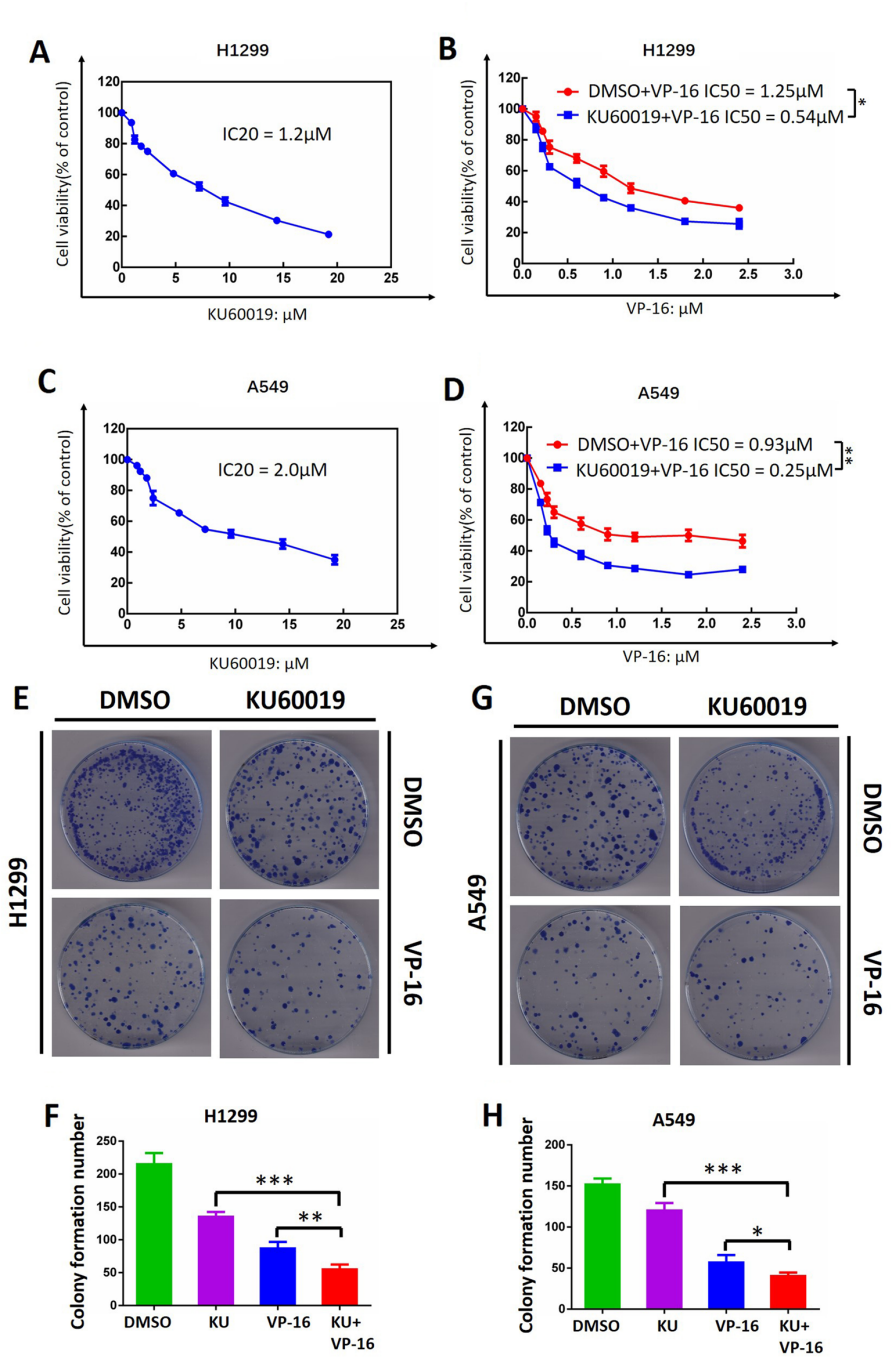
KU60019 Combined with VP-16 suppressed the survival of lung cancer cells. (**A**, **C**) Cells were treated with the indicated concentrations of KU60019 for 72 h, followed by the ATP-lite assay. (**B**, **D**) Cells were treated with various concentrations of VP-16 or in conjunction with an IC_20_ concentration of KU60019 and subjected to the ATP-lite assay. (**E**–**H**) H1299 and A549 cells were plated in triplicate in 60-mm dishes and treated with VP-16 (0.5 μM) alone or in conjunction with KU60019 (1 μM). After 10–14 days, colonies were stained and counted (> 50 cells in a colony). All experiments were independently repeated three times.

Given that ATM triggers the DNA checkpoint response and enhances the repair of broken chromosomes, thereby controlling genome stability and cell survival. Next, we investigate whether KU60019 could enhance VP-16-mediated suppression of survival in lung cancer cells. First, we treated H1299 and A549 cells with various concentrations of KU60019 to identify the IC_20_ values of H1299 and A549, respectively. The ATPlite cell viability assay proved that KU60019 with no remarkable cytotoxic at up to 1.2 μM or 2 μM in the case of H1299 and A549 cell lines (Fig. 5A, C), indicating that KU60019 is a satisfactory ATM inhibitor with excellent effectiveness against ATM and negligible cytotoxicity. Next, we used the IC_20_ concentration of KU60019 in conjunction with numerous concentrations of VP-16 to investigate the IC_50_ values of VP-16 with or without KU60019. In H1299 and A549 cells, the ATPlite cell viability assay exhibited that KU60019 led to a substantial decline in the IC_50_ values of VP-16 from 1.25 μM to 0.54 μM and from 0.93 μM to 0.25 μM, respectively (Fig. 5B, D). As a conclusion of these results, KU60019 was found to sensitize lung cancer cells to VP-16 stimuli. Furthermore, the colony formation assay was performed to evaluate whether KU60019 could synergistically suppress lung cancer cell survival with VP-16. As shown in Fig. 5E–H, VP-16 treatment significantly reduced colony formation. Furthermore, compared with the VP-16-only group, the combinatorial regimen (VP-16+KU60019) was remarkably inhibiting colony formation. Taken together, KU60019 synergistically suppresses lung cancer cell survival with VP-16.

### A combination of KU60019 and VP-16 promotes the apoptosis of lung cancer cells

**Figure 6:**
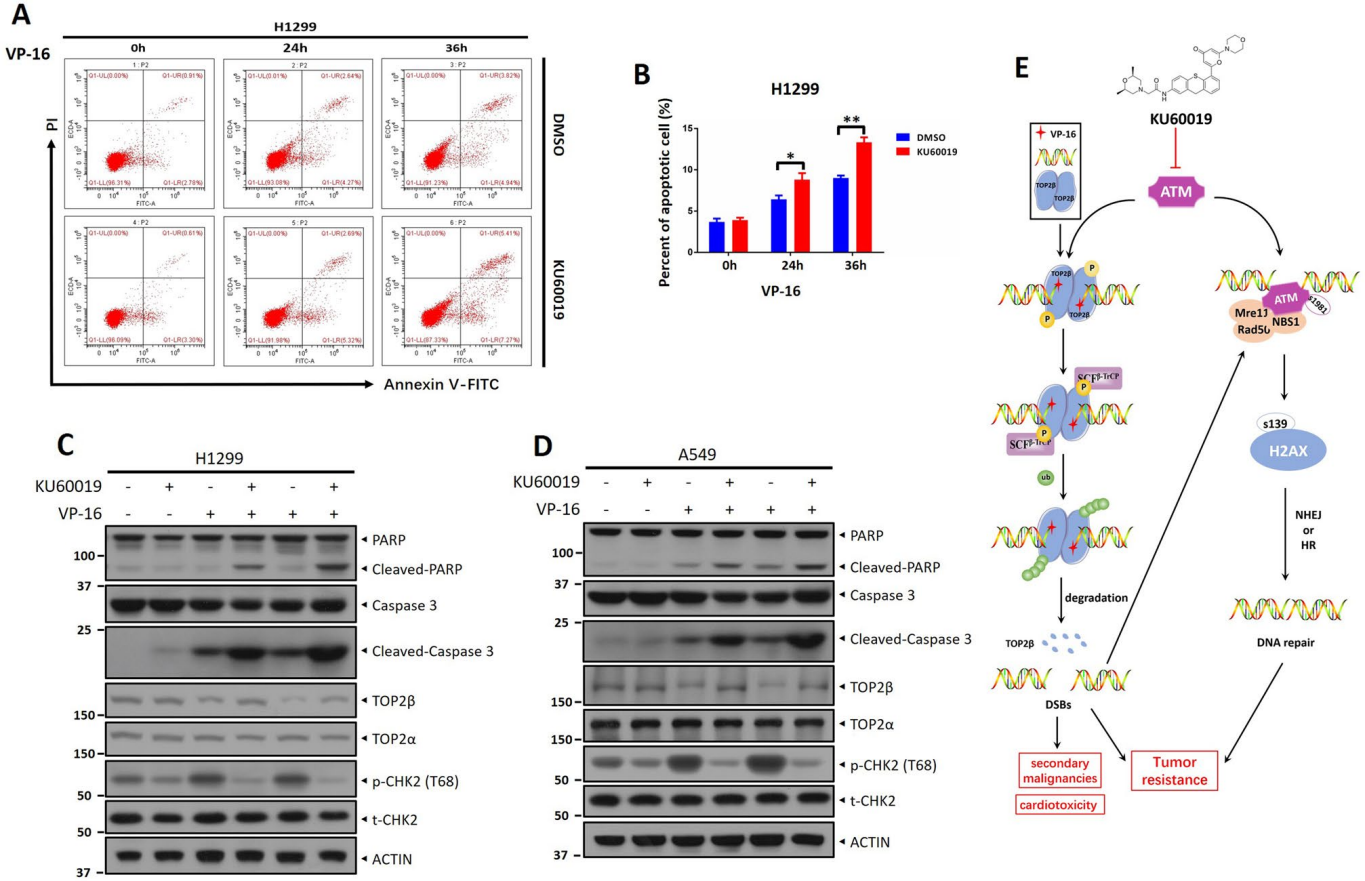
A combination of KU60019 and VP-16 promotes the apoptosis of lung cancer cells. (**A**, **B**) H1299 cells were treated with VP-16 (10 μM) alone or in conjunction with KU60019 (5 μM) for the indicated time periods and then subjected to FACS analysis to determine the apoptotic population. (**C**, **D**) H1299 and A549 cells were incubated with various concentrations of KU60019 or VP-16 alone or in combination for 24 h. Cells were harvested for western blotting using indicated antibodies. All experiments were independently repeated three times. According to the molecular weight, the nitrocellulose membrane was cut prior to hybridization with antibodies and the original blots are presented in Supplementary Information. Molecular weight markers are noted to the left of blot figure.

Chemotherapeutic drugs targeting TOP2 have been shown to degrade TOP2β rapidly and cause apoptosis in cells. Lastly, we investigated whether KU60019 and VP-16 could synergistically induce cell apoptosis. Apoptosis was determined by FACS analysis of H1299 cells treated with VP-16 with or without KU60019 for the indicated periods. FACS analysis showed that the combinatorial regimen induced significantly more apoptosis than VP-16 stimuli alone, as evidenced by the higher proportion of Annexin V+ individual cells in the combinatorial regimen group (Fig. 6A, B). Likewise, a combination of KU60019 and VP-16 remarkably elevated the level of cleavage PARP and caspase-3, two marks of apoptosis, compared with VP-16 alone, in both H1299 and A549 cells (Fig. 6C, D). Meanwhile, KU60019 significantly blocked ATM activation and caused an accumulation of TOP2β induced by VP-16 treatment, as reflected by the changes in phosphorylation level at T68 on CHK2 (Fig. 6C, D). Collectively, these data suggested that KU60019 enhances VP-16-induced apoptosis synergistically.

## Discussion

Chemotherapeutic drugs targeting TOP2 (TOP2 poisons) play important roles in human cancer management. For example, teniposide is commonly used to treat some brain cancers, Hodgkin's lymphoma, and acute lymphoblastic leukemia (ALL) in children. Etoposide (VP-16) is also authorized for the treatment of solid tumors, including lung cancer and testicular cancer. Doxorubicin is licensed to treat a variety of human cancers^1,24^. However, like any other chemotherapy, treatment with this class of drugs causes unwanted side effects, such as cardiovascular toxicity and secondary malignancies^10,11^. For example, chromosomal translocation and therapy-related secondary acute leukemia (t-AL) are often observed in a high proportion of patients treated with TOP2 poisons, including etoposide, epirubicin, mitoxantrone and anthracyclines^25,26^. The side effects were related to their ability to cause DNA damage. Previous reports have demonstrated that although both the TOP2α and TOP2β contribute to the anti-tumor effect of TOP2-targeted drugs, mounting evidence suggests that TOP2β is the primary isoform responsible for the genotoxic effects of TOP2 poisons and initiating TOP2–associated secondary malignancies and cardiovascular toxicity^10,25,27,28^. For example, cardiomyocyte-specific deletion of TOP2β protects cardiomyocytes from doxorubicin-induced DNA double-strand breaks and transcriptome changes which are responsible for defective mitochondrial biogenesis and ROS formation, thereby protecting mice from the development of doxorubicin-induced progressive heart failure^29^. Additionally, the adult heart exclusively expresses TOP2β^27^. In contrast, TOP2α is typically expressed in quickly growing cancer cells and is essential for cell growth. Therefore, TOP2α-specific agents may reduce unwanted genotoxic DNA damage while maintaining the anticancer cytotoxic activity and TOP2α, instead of TOP2β, is a favorable target for anticancer drug development^28^. However, at present no truly TOP2α or TOP2β specific drugs are available for clinical use. Despite this, TOP2 poison remains an indispensable part of cancer therapy. Therefore, it's crucial to pinpoint the factors that determine how serious side effects are and to create tumor chemosensitizers that are both effective and safe.

In many instances, to reduce the toxicity of TOP2-target anticancer drugs or other primarily clinical considerations, the preclinical and clinical research on combinatorial regimens that incorporate multimodal therapy has been comprehensively done and has proven extraordinarily successful. For instance, ICRF-187 has been adopted in the clinic as a cardio-protectant against doxorubicin cardiotoxicity^30^, Oncolytic HSV and etoposide combinatorial therapy enhanced antitumor activity, and reduced toxicity^31^.

It has been shown that TOP2 poisons combined with PARP1 inhibitors can reduce the risk of secondary malignancies^32^, targeting myeloperoxidase activity reduce TOP2 poisons induced genetic damage that leads to therapy-related leukemia^33^, and TOP2 poison hypersensitivity may result from DNA-PKcs inhibitors^34^. It may therefore be possible to significantly increase therapeutic efficacy while reducing toxicity by developing combination strategies that target TOP2 via multiple pathways. It has been reported that the protein level of TOP2^35^, increased expression of ABC transporters, such as MDR1 and MRP1^36^, expression of DNA mismatch repair-related genes^37^, and the degradation of TOP2-DNA cleavage complex are the leading factors responsible for the therapeutic effect of TOP2 poison. In our previous study, we found that upon the TOP2 poison stimuli, ATM attaches to and phosphorylates TOP2β, thereby facilitating the ubiquitination and degradation of TOP2β by SCF^β-TrCP^. Thus, this finding inspired us to evaluate the therapeutic potential of the combination of TOP2 poison and ATM inhibitors against cancer cells. In this study, we demonstrate the following: 1) KU60019, an ATM inhibitor interferes with the association of ATM with TOP2β and blocks TOP2β ubiquitination, thereby causing a dose and time-dependent accumulation of TOP2β; 2) Inactivation of ATM by KU60019 increases the level of the TOP2β-DNA cleavage complex and impairs the repair of VP-16 induced DSBs; 3) the therapeutic combination regimen synergistically suppressed the survival of lung cancer cells by promoting apoptosis.

ATM is among the central kinases that participated in the cellular response to DNA DSBs, which regulates cell cycle DNA checkpoint response and promotes the repair of broken chromosomes by either NHEJ or HR pathways^18^. Much data has emerged that supports the involvement of ATM in many other signaling pathways, including metabolism and growth of cells, oxidative stress, and chromatin remodeling, over the past few decades^22^. ATM, therefore, has been linked to cancer since its discovery. Additionally, it is well established that tumor initiation and tumorigenicity are suppressed by ATM^22,38^. As an example, A-T patients are more likely to develop cancers, especially lymphoma, and leukemia^39^. Furthermore, the lack of ATM increases the predisposition of mice to lymphoid tumors, epithelial tumors, and intestinal tumors^39,40^. However, in post-formed tumors, recent studies suggest that ATM signaling can also be advantageous to cancer cells and several cancer cells have an upregulated ATM signaling rather than a downregulated ATM signaling^22^. It is reported that for HER2-dependent breast tumorigenesis, ATM expression, as well as its activity, are crucial^41^, and inhibiting ATM causes EMT to be reversed, thus through JAK/STAT3/PD-L1 pathway, attenuating the metastatic potential of cisplatin-resistant lung cancer cells^42^. AKT, ERK, and Wnt signaling pathways, among others, have all been reported to be activated by ATMs and play a role in cell migration and proliferation^43^. Similarly, in cancer cells, cell migration and invasion are sustained by oxidative stress activation of ATM^44^. Taken together, ATM seems to have a much more complicated role than just serving as a tumor suppressor during the onset and progression of cancer. Because of this, to treat cancer, ATM inhibitors have been created^45^. However, it has not been studied whether ATM inhibitors combined with TOP2 poison are effective in treating lung cancer. In the current investigation study, we discovered that VP-16 resistance may also be mediated through ATM activation, and we demonstrate that the ATM inhibitor KU60019 enhances VP-16's ability to suppress tumors synergistically. Mechanistically, this synergy may work in two ways, on the one hand, KU60019 stabilizes TOP2βcc by inhibiting TOP2β degradation, thereby contributing to genome and increasing the TOP2β-concealed DSBs, leading to accelerated cell death (Fig. 6E), on the other hand, inhibitors of ATM by KU60019 are expected to suppress DSB DNA repair, as reflected by the reduction of γH2AX levels and foci formation, thereby blocking checkpoint controls and leading cell apoptosis (Fig. 6E).

In summary, we provide a new approach that ATM inhibitors combined with TOP2 poison would synergistically suppresses lung cancer cell survival as well as reduce DNA damage responses, thus may lowering the possibility of cardiotoxicity and secondary malignancy linked to therapy. While, further study is needed to systematically investigate whether the combinatorial regimen is a safe and effective combination in the whole organism or human.

## Materials and methods

### Cell lines and chemicals

The American Type Culture Collection (ATCC, Manassas, VA) provided the human lung cancer cell H1299, A549 cells, and HEK293 cells, which were cultured in DMEM media containing 1% antibiotic–antimycotic and 10% FBS. In a humidified 5% CO_2_ incubator, all cells were maintained at 37 °C. From MedChemExpress, etoposide and KU60019 were procured. DMSO and cycloheximide were procured from Sigma-Aldrich and MG132 from Cayman. For inhibition studies, sub-confluent cells were incubated with VP-16 and KU60019 at several concentrations for variable times.

### Immunoblotting and immunoprecipitation

For immunoblotting (IB), in 60-mm dishes, cells were cultured and incubated with the specified chemicals. The next step was to extract the cells, lyse them in a buffer that contained phosphatase and protease inhibitors, and then perform ultrasound procedures. The supernatant was harvested. Employing the BCA protein assay kit, the protein concentration was calculated. Employing the indicated Abs, immunoblotting was also carried out.

Cells were lysed for 30 min on ice in NP-40 lysis buffer with protease inhibitors for immunoprecipitation (IP). The whole-cell lysates were harvested and incubated for 12 h at 4 °C with the beads-conjugated FLAG or beads-conjugated HA in a rotating incubator. The immune precipitates were then rinsed four times utilizing a lysis buffer. After separation on SDS-PAGE, IB was performed with the indicated Abs, as stated earlier^17^.

The following antibodies were used: TOP2α (12286, Cell Signaling Technology, 1:1000), TOP2β (611493, BD Biosciences, 1:1000), p-CHK2 (ab32148, Abcam, 1:1000), CHK2 (2662, Cell Signaling Technology, 1:1000), p-AKT (4060, Cell Signaling Technology, 1:2000), AKT (4691, Cell Signaling Technology, 1:2000), FLAG (F1804, Sigma, 1:2000), ACTIN (A5441, Sigma, 1:10000), p-ATM (200-301-400S, Rockland, 1:1000), ATM (2873, Cell Signaling Technology, 1:1000), p-S/T (9631, Cell Signaling Technology, 1:1000), PARP (9542, Cell Signaling Technology, 1:1000), Caspase 3 (9665, Cell Signaling Technology, 1:1000) and γH2AX (05-636, Millipore, 1:10000).

### Flow cytometry

Into 6-well plates, cells were seeded. After at least 12 h incubation, VP-16 alone or in conjunction with KU60019 was added. The cells were collected after 24 or 48 h of exposure to 0.05% trypsin (Gibco), mixed with 500 μl × binding buffer, and stained with an annexin V-FITC/propidium iodide (PI) apoptosis detection kit (Beyotime Biotechnology, C1063), in accordance with the manufacturer’s instructions, and then flow cytometry was employed for counting, as stated earlier^46^.

### Neutral comet assay

As mentioned earlier, neutral comet tests were carried out^47^. First, 2 mM thymidine was utilized to treat the cells that were cultured in 60-mm dishes., The cells were rinsed with PBS after 24 h and treated with VP-16 or KU60019 alone or in combination for an additional 2 h. Following cell harvesting, the slide was coated with the cells. Slides were immersed in neutral N1 lysis solution for an overnight period at 37 °C to cause cellular lysis. After that, the slides were stained with 10 μg/ml propidium iodide (PI) for 30 min, and electrophoresis was performed on the cells for 25 min at 15 V (0.6 V/cm) before being observed under a fluorescence microscope. The CometScore software was used to analyze the tail moment of the comet.

### Immunofluorescence

After treatment, cells were fixed and stained as stated earlier utilizing DAPI and anti-γH2AX antibody^17^. Cells were ultimately observed and photographed under fluorescence employing the microscope.

### TOP2cc-flow cytometry assay

TOP2cc-flow cytometry assay was executed as earlier published^23^. In brief, for the treatment of cells, VP-16 alone or in conjunction with KU60019 was used at varying times. Then, cells were collected and resuspended in PHEM buffer containing 2 mM PMSF. Next, a 4% paraformaldehyde solution was employed to fix the cells, and proteins were extracted by adding the extraction buffer. Finally, following blocking, the cells were then labeled with anti-TOP2β antibodies. The TOP2βcc and DNA were then labeled by appropriate fluorescein-conjugated secondary antibody and propidium iodide. The TOP2βcc levels were counted and analyzed by flow cytometry.

### The TARDIS assay

TOP2 intermediates on genomic DNA were generated by etoposide-treated cells. After treatment, cells are immobilized in agarose on a glass slide. After washing and extracting the slides with SDS and salt, the majority of cellular components are eliminated, but genomic DNA and covalent adducts remain. Anti-TOP2β polyclonal antibody and Alexa Fluor 568-conjugated secondary antibody are then used to label adducts. The slides were then stained for 15 min with DAPI and coverslips were put and fastened. Then, images of red (Alexa Fluor 568-stained covalently bound TOP2) and blue (DAPI-stained DNA) immunofluorescence were taken using an epifluorescence microscope equipped with a slow scan charge-coupled camera. TARDIS analysis measured the TOP2β-DNA cleavage complex as reported previously^48^.

### Cell viability and clonogenic survival assays

The cell viability was assessed employing the ATPlite 1-step Luminescence Assay System (PerkinElmer), as stated earlier^17^. In brief, cells were seeded in triplicate in 96-well plates at a density of 2 × 103 cells per well, treated for 72 h with different concentrations of VP-16 or in conjunction with KU60019, and then the manufacturer's recommendations for ATPlite cell viability assaying were followed. Plots of the findings from three separate trials were generated.

For clonogenic survival assays, the cells were seeded on 60-mm dishes coated with 0.1% gelatin (Sigma, V900863) and allowed to adhere overnight. After that, the culture medium was refreshed and supplemented with DMSO, KU60019, and VP-16 alone or the combinatorial regimen for the following days. After 7–14 days, cell culture was terminated. For colony counting, colonies were photographed after being stained with coomassie brilliant blue solution.

### Statistical analysis

Data are presented as the means ± standard deviation (SD) of three independent measurements. To compare parameters between groups, Statistical Program for the Social Sciences 18 20.0 (SPSS, Chicago, IL, USA) was utilized to conduct a two-tailed Student's t-test for statistical analysis. A P value of < 0.05 was considered statistically significant.

### Supplementary Information

Supplementary Information.

## Data Availability

The authors declare that all data supporting the findings of this study are available with the article or from the corresponding author upon reasonable request.
