# Author Correction: ATM inhibitor KU60019 synergistically sensitizes lung cancer cells to topoisomerase II poisons by multiple mechanisms

**DOI:** 10.1038/s41598-023-40952-6

**Published:** 2023-08-24

**Authors:** Jianfeng Shu, Xiaofang Wang, Xuejie Yang, Guofang Zhao

**Affiliations:** 1 HwaMei Hospital, University of Chinese Academy of Sciences, 41 Xibei Road, Ningbo, 315010 Zhejiang China; 2 Ningbo Institute of Life and Health Industry, University of Chinese Academy of Sciences, Ningbo, 315000 Zhejiang China

Correction to: *Scientific Reports* 10.1038/s41598-023-28185-z, published online 17 January 2023

The original version of this Article contained errors in Figure 4 and Supplementary Figure S4, the legends of Figures 3, 5 and 6, and the Acknowledgements section.

In Figure 4, panel H, as well as in Supplementary Figure S4, the ACTIN panels of the “A549” cell lines were incorrect. The original Figure 4 and accompanying legend appear below.

**Figure 4:**
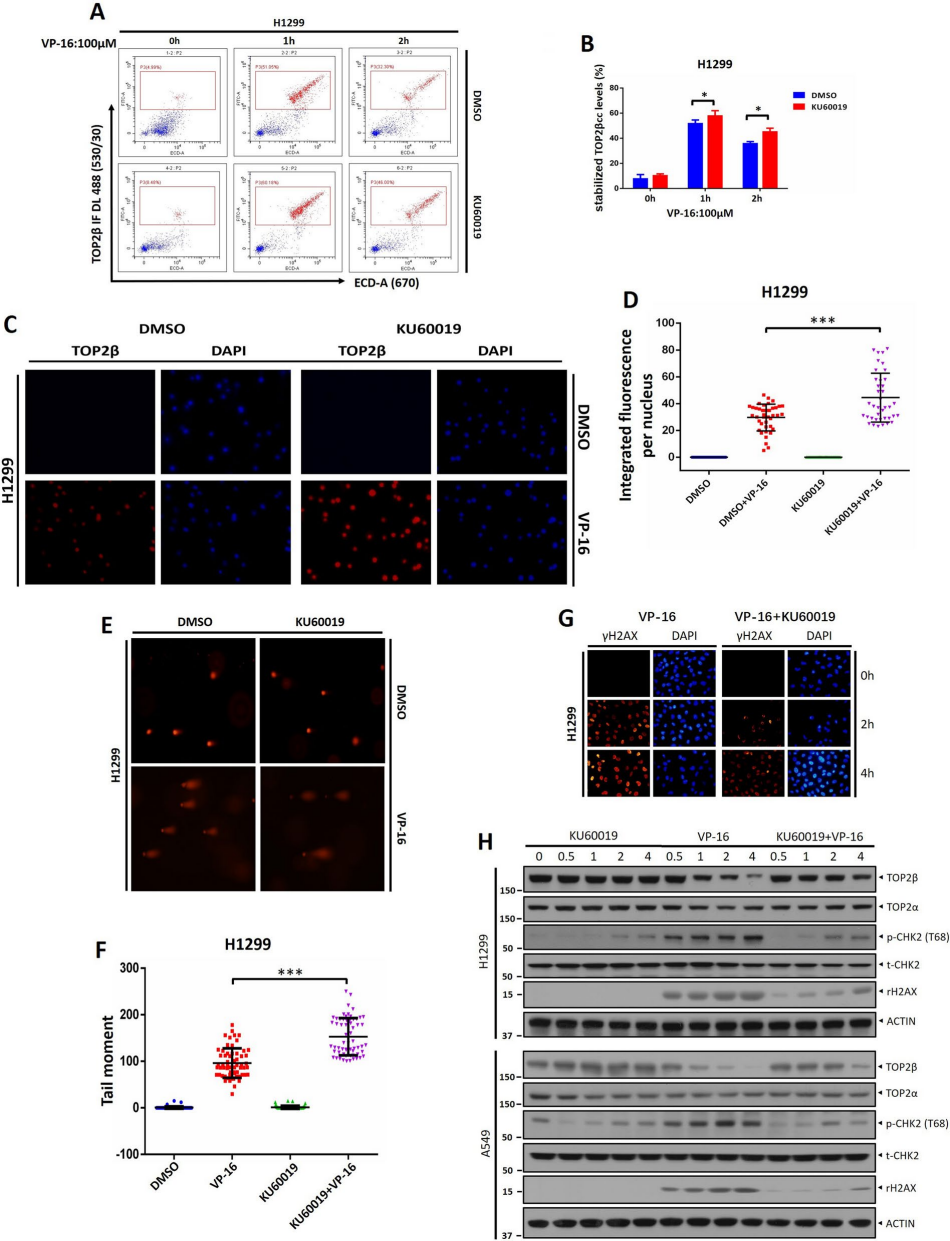
KU60019 increases the level of the TOP2β-DNA cleavage complex and impairs the repair of VP-16-induced DSBs. (**A**, **B**) H1299 cells were treated with VP-16 alone or in conjunction with KU60019 for the appropriate times, and then the TOP2cc levels were determined by FACS. (**C**, **D**) Two hours were spent treating H1299 cells with VP-16 or VP-16 in conjunction with KU60019 (5 μM). The cells were then collected for the TARDIS assay. (**E**, **F**) H1299 cells were pretreated with KU60019 (5 μM) for 1 h and then cotreated with VP-16 for an additional 1 h. Cells were then harvested for the neutral comet assay. Representative images are shown, and the data are presented as the mean ± S.D. from three independent experiments; ***p < 0.001. (**G**) Cells treated with VP-16 alone or in conjunction with KU60019 for the indicated time periods were harvested at the indicated time points for immunofluorescence. (**H**) H1299 and A549 cells were treated with KU60019 or VP-16 alone or in conjunction with IB with the indicated Abs. All experiments were independently repeated three times. According to the molecular weight, the nitrocellulose membrane was cut prior to hybridization with antibodies and the original blots are presented in Supplementary Fig. 4. Molecular weight markers are noted to the left of blot figure.

In addition, descriptions of panels C and D were incorrectly included in the legend of Figure 3, which have now been removed. And,

“HEK293 cells transfected with the specified plasmids were treated with VP-16 and MG132 (20 μM) or in association with RG7112 (5 μM) for 5 h, and then IP was performed with anti-HA beads, and direct IB was performed with the selected antibodies.”

now reads:

“HEK293 cells transfected with the specified plasmids were treated with VP-16 and MG132 (20 μM) or in association with KU60019 (5 μM) for 5 h, and then IP was performed with anti-HA beads, and direct IB was performed with the selected antibodies.”

In the legend of Figure 5,

“(**E**–**H**) H1299 and A549 cells were plated in triplicate in 60-mm dishes and treated with VP-16 (0.5 μM) alone or in conjunction with RG7112 (1 μM).”

now reads:

“(**E**–**H**) H1299 and A549 cells were plated in triplicate in 60-mm dishes and treated with VP-16 (0.5 μM) alone or in conjunction with KU60019 (1 μM).”

In the legend of Figure 6,

“(**C**, **D**) H1299 and A549 cells were incubated with various concentrations of KU60019 or VP-16 for 24 h, followed by MK-2206 (0.5 or 1 μM) or DMSO for 24 h.”

now reads:

“(**C, D**) H1299 and A549 cells were incubated with various concentrations of KU60019 or VP-16 alone or in combination for 24 h.”

Additionally, the Acknowledgements section contained errors, where information about funders that did not support this study was included. The Acknowledgements section now reads:

“This work was supported by the Natural Science Foundation of Zhejiang Province (LY22H160013 to J.S.); the Natural Science Foundation of Ningbo (202003N4205 to J.S.); the Research Foundation of Ningbo Institute of Life and Health Industry, University of Chinese Academy of Sciences (2020YJY0209 to J.S.).”

The original Article and accompanying Supplementary Information file have been corrected.

