# Author Correction: ATM inhibitor KU60019 synergistically sensitizes lung cancer cells to topoisomerase II poisons by multiple mechanisms

**DOI:** 10.1038/s41598-024-59332-9

**Published:** 2024-04-16

**Authors:** Jianfeng Shu, Xiaofang Wang, Xuejie Yang, Guofang Zhao

**Affiliations:** 1 HwaMei Hospital, University of Chinese Academy of Sciences, 41 Xibei Road, Ningbo, 315010 Zhejiang China; 2 Ningbo Institute of Life and Health Industry, University of Chinese Academy of Sciences, Ningbo, 315000 Zhejiang China

Correction to: *Scientific Reports* 10.1038/s41598-023-28185-z, published online 17 January 2023

The Supplementary Information published with this Article contained errors. Due to errors in the assembly of the original immunoblot image, the bands labelled as Figure 3A p-TOP2β, Figure 4H p-CHK2, and Figure 6C ACTIN do not match precisely with the corresponding bands in the main manuscript. The original Supplementary Information files are provided below.

These errors have now been corrected in the Supplementary Information that accompanies the original Article.

### Supplementary Information

Supplementary Information 1.

Supplementary Information 2.

Supplementary Information 3.

Supplementary Information 4.

Supplementary Information 5.

