# Supplementary material for: ATM inhibitor KU60019 synergistically sensitizes lung cancer cells to topoisomerase II poisons by multiple mechanisms
Source: Sci Rep. 2023 Jan 17;13:882. doi: 10.1038/s41598-023-28185-z (PMC9845372; doi:10.1038/s41598-023-28185-z)

Figure 1A and 1B

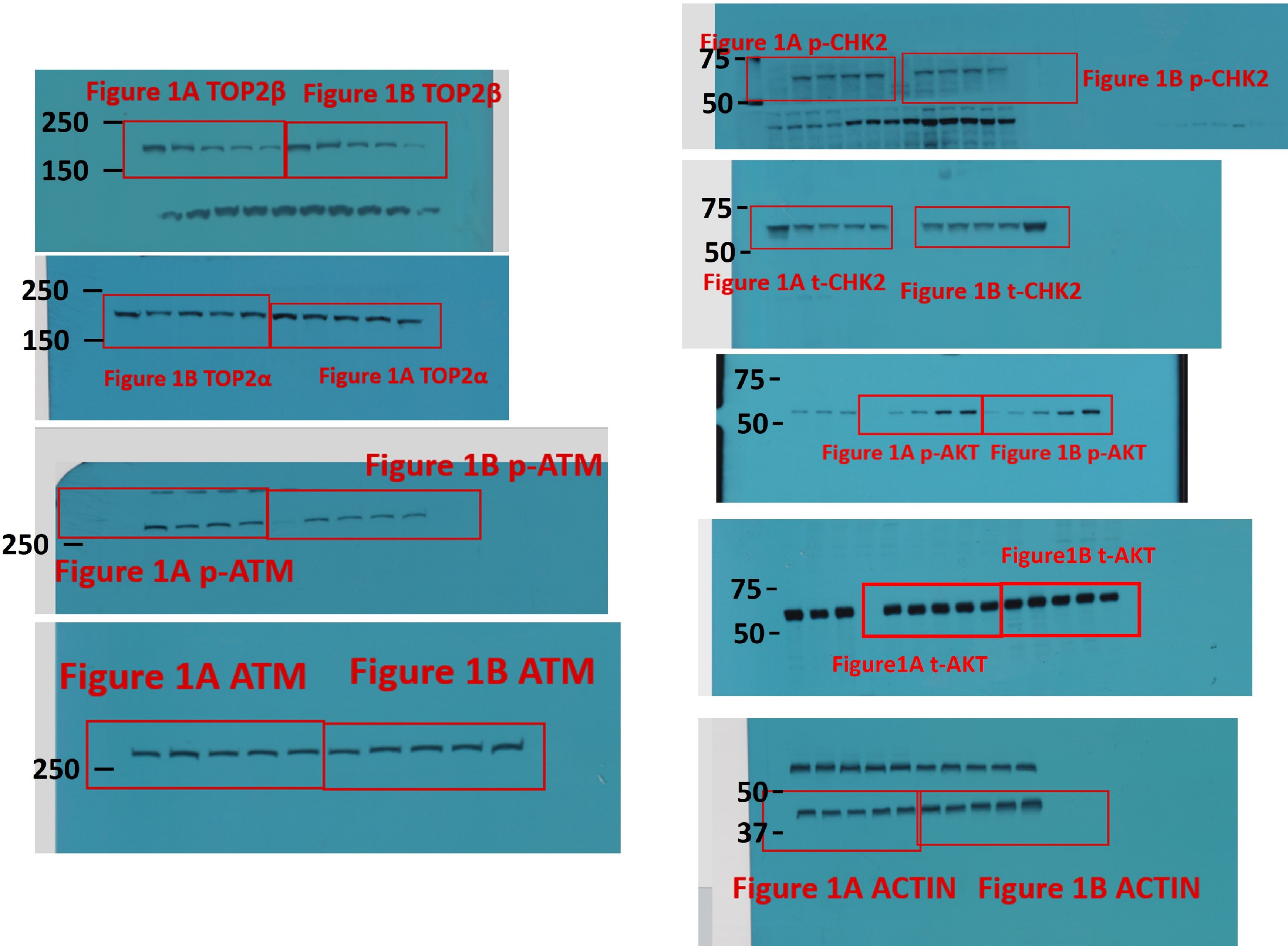

Figure 1C and 1D

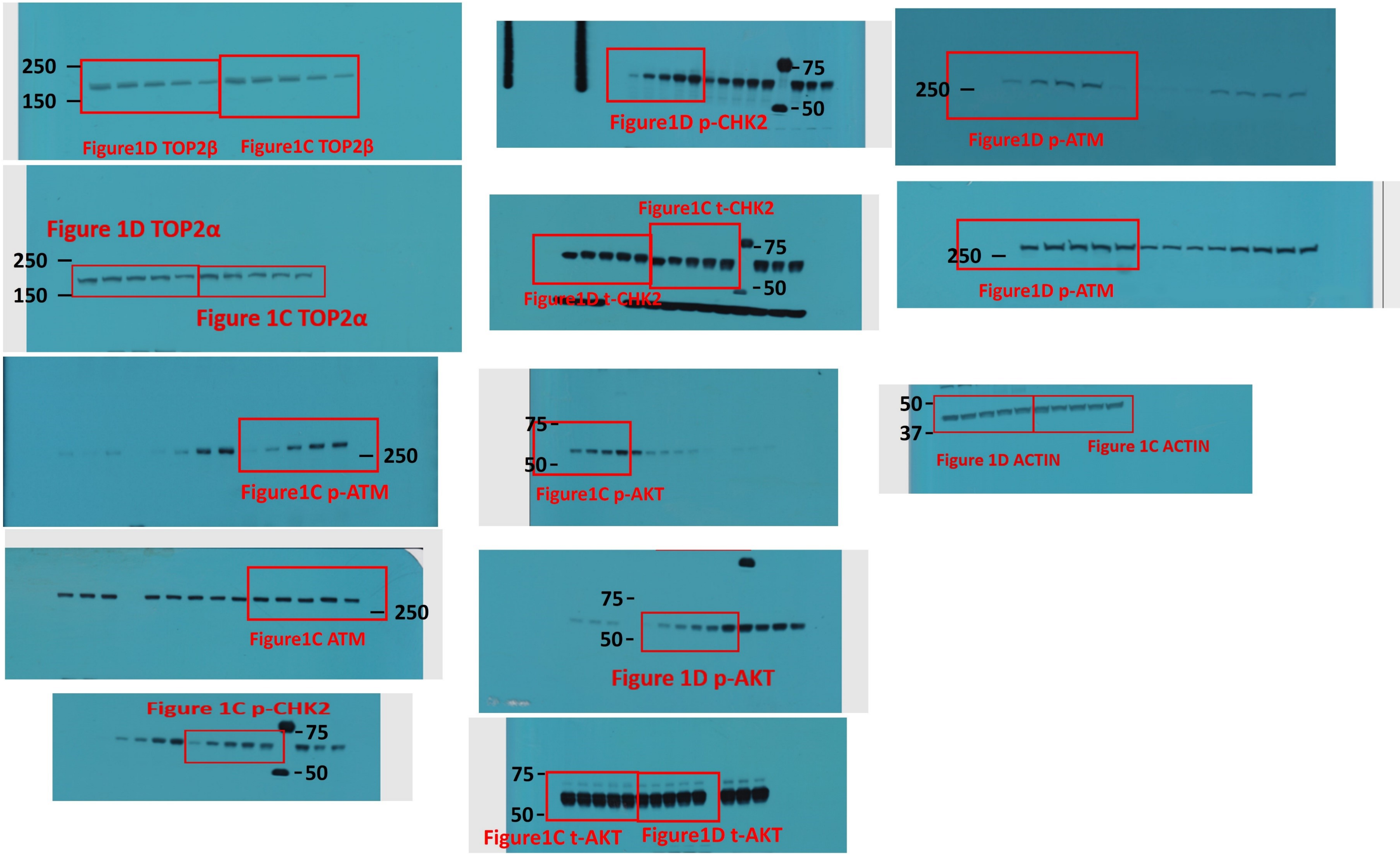

Figure 2A

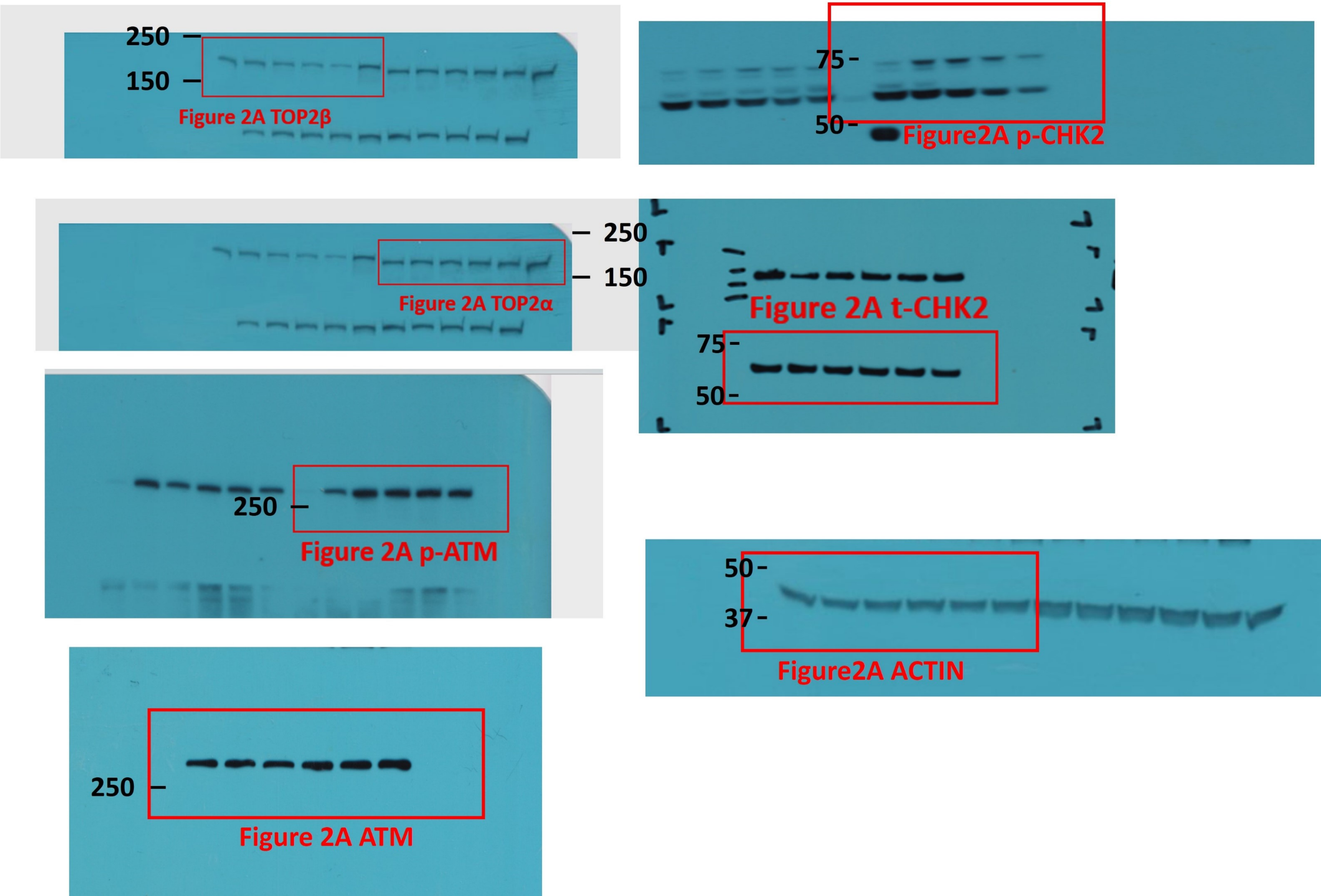
250
150
Figure 2A TOP2β
75
50
Figure2A p-CHK2
250
150
Figure 2A TOP2α
Figure 2A t-CHK2
75
50
250
Figure 2A p-ATM
50
37
Figure2A ACTIN
250
Figure 2A ATM

Figure 2B

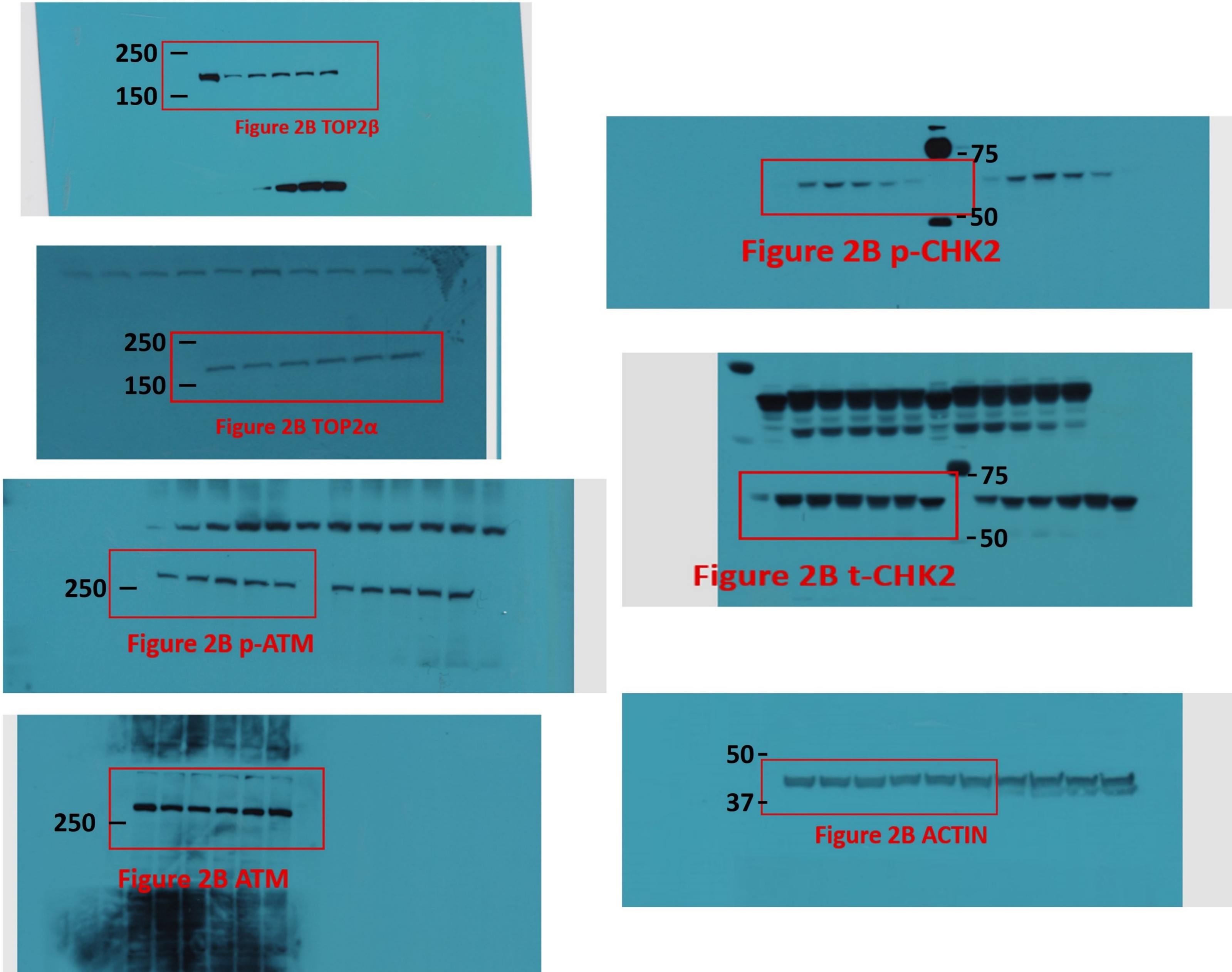
250
150
Figure 2B TOP2β
75
50
Figure 2B p-CHK2
250
150
Figure 2B TOP2α
75
50
Figure 2B t-CHK2
250
Figure 2B p-ATM
50
37
Figure 2B ACTIN
250
Figure 2B ATM

Figure 2C and 2D

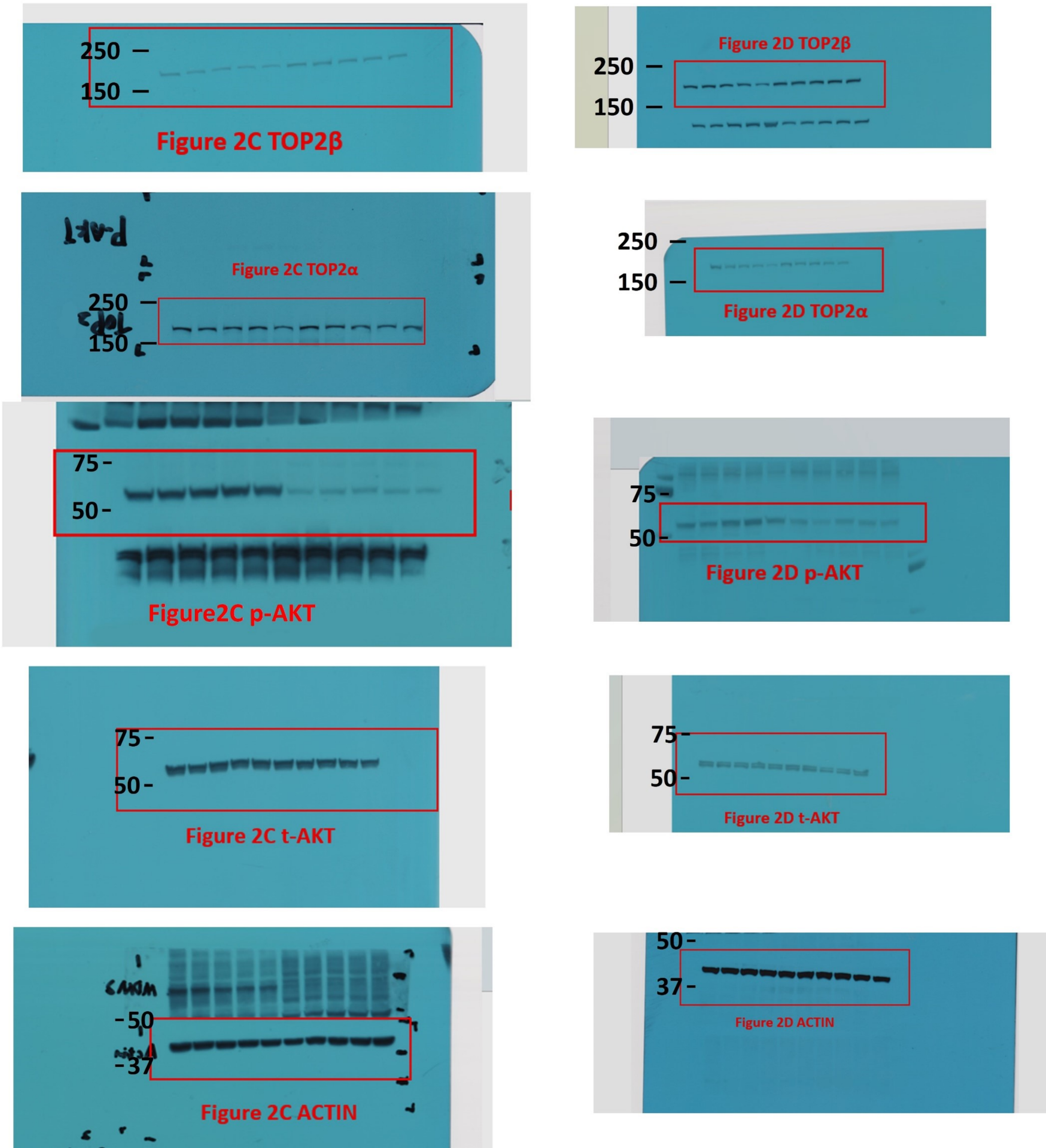

Figure 2E and 2F

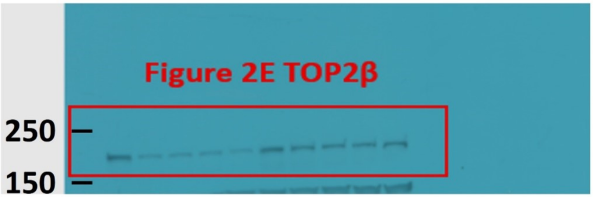

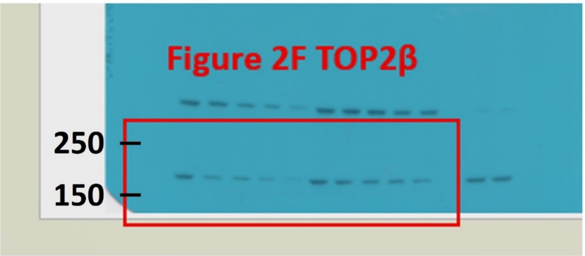

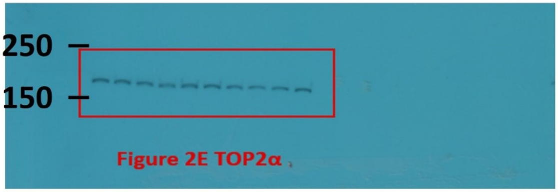

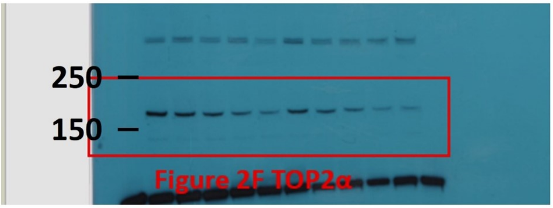

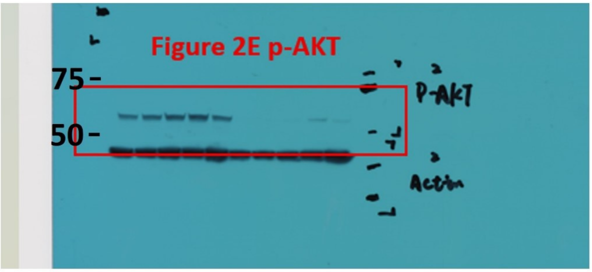

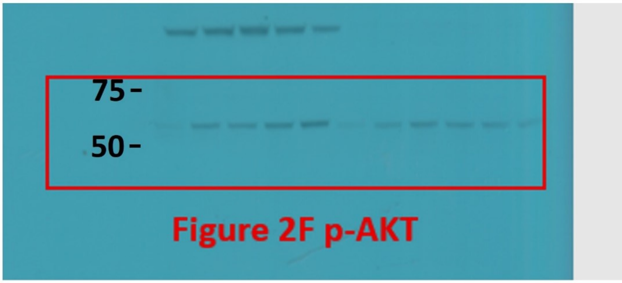

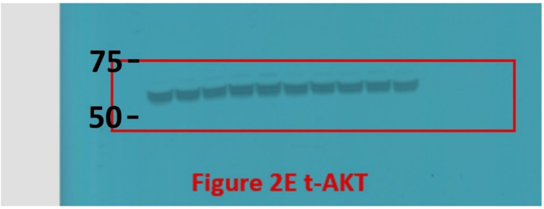

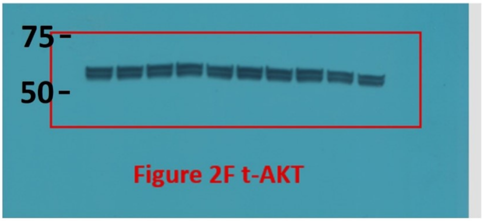

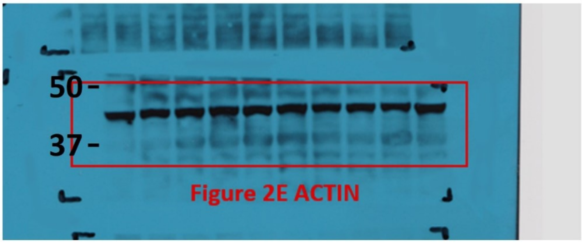

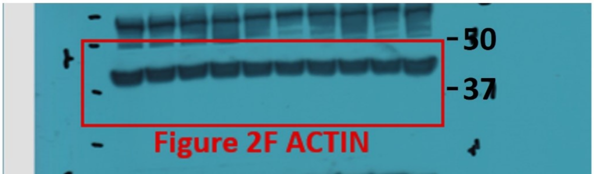

Figure 3A and 3B

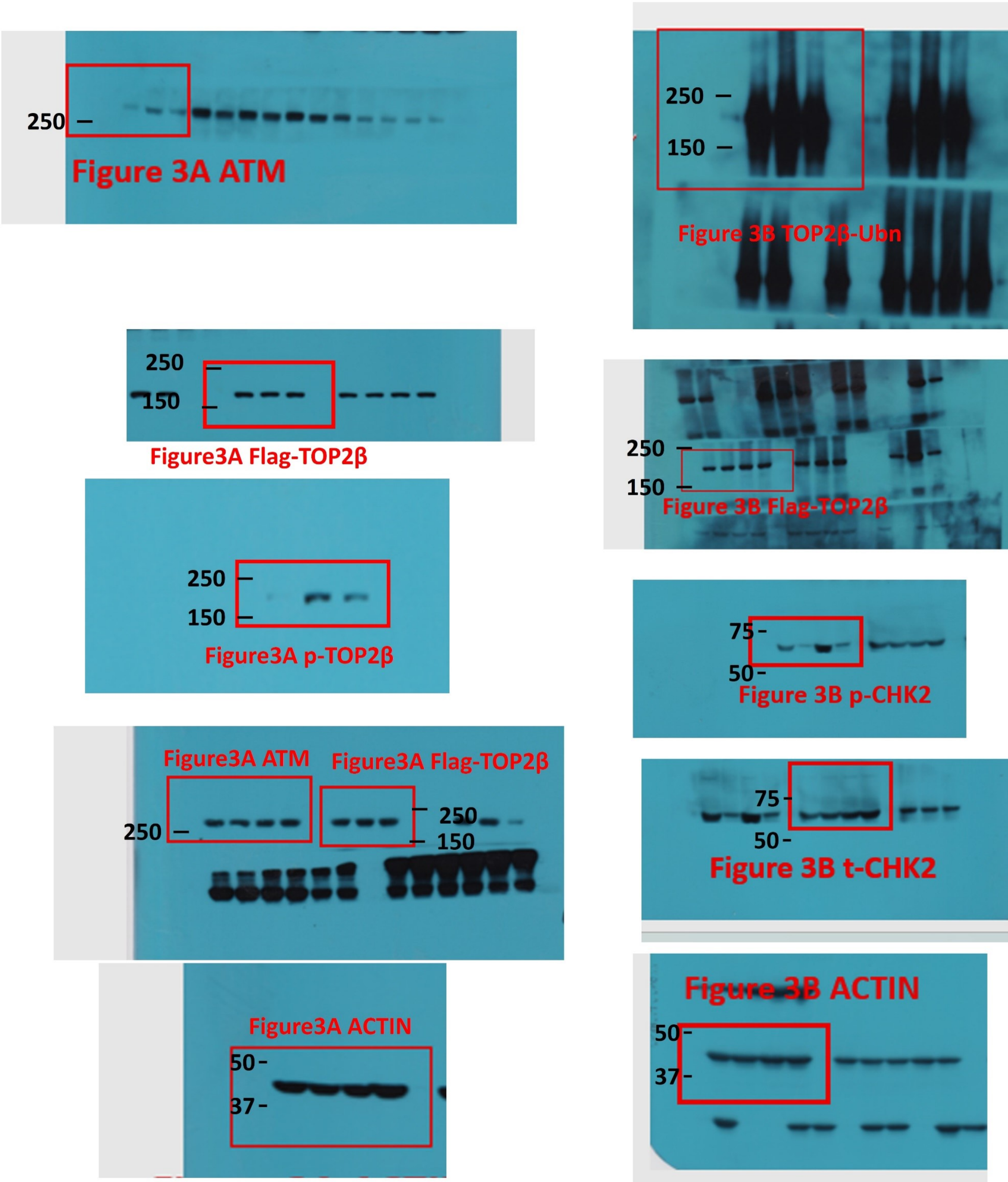

Figure 4H

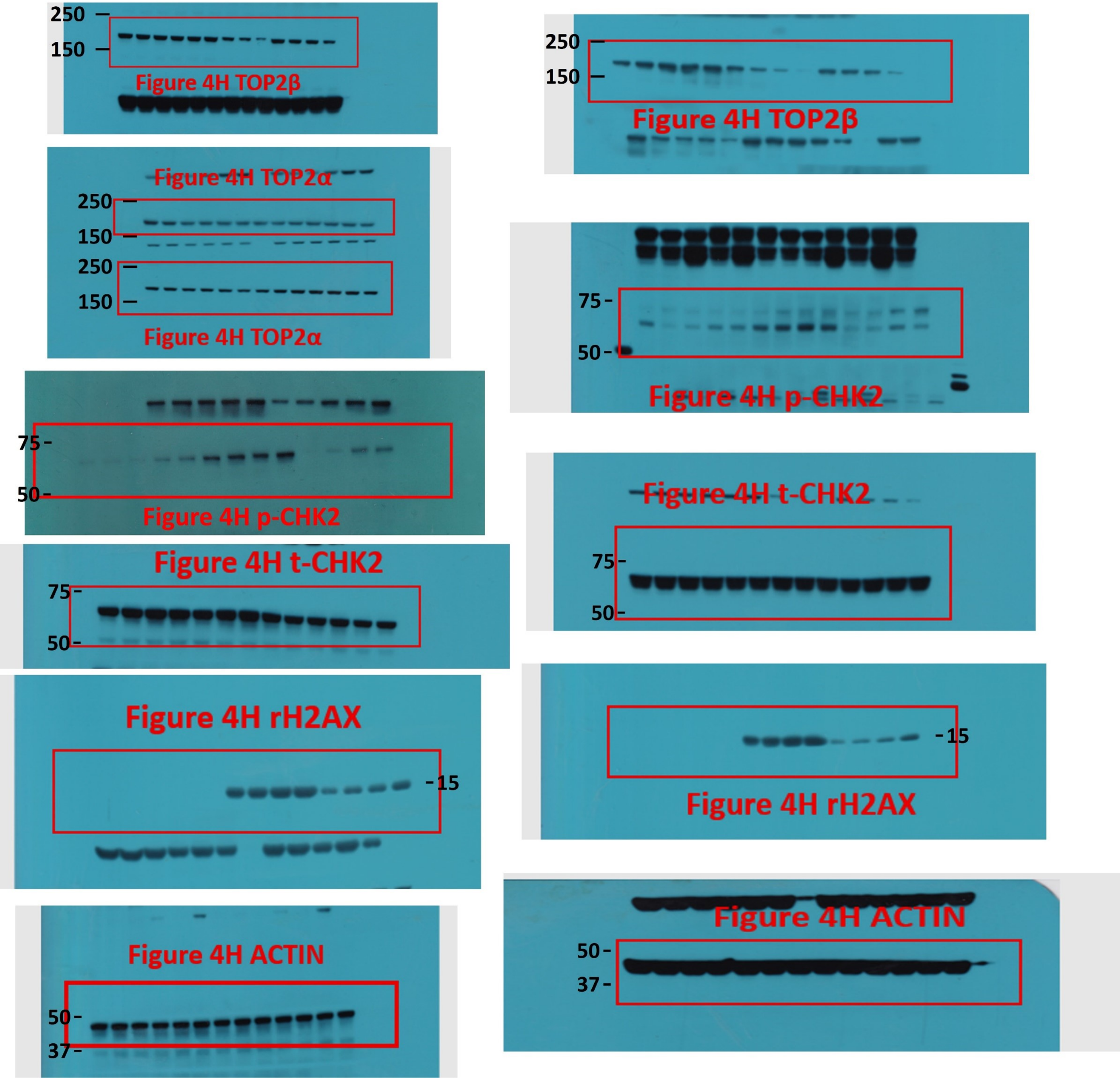
250
150
Figure 4H TOP2β
Figure 4H TOP2α
250
150
250
150
Figure 4H TOP2α
75
50
Figure 4H p-CHK2
Figure 4H t-CHK2
75
50
Figure 4H rH2AX
15
Figure 4H ACTIN
50
37
250
150
Figure 4H TOP2β
75
50
Figure 4H p-CHK2
Figure 4H t-CHK2
75
50
15
Figure 4H rH2AX
Figure 4H ACTIN
50
37

Figure 6C and 6D

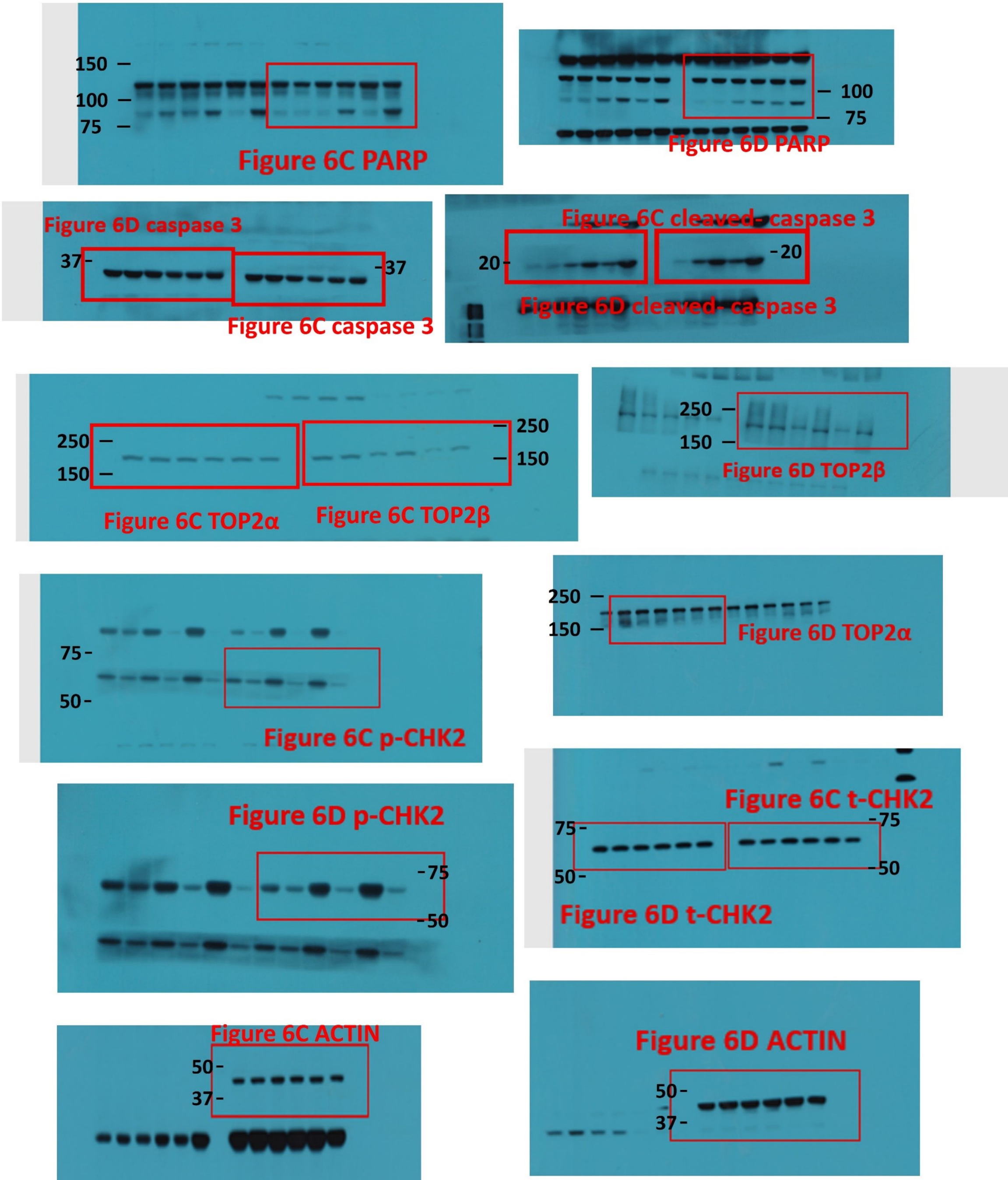

Supplement: Supplementary file 1 — Supplementary Information. [file 41598_2023_28185_MOESM1_ESM.pdf]
