# Supplementary material for: Author Correction: ATM inhibitor KU60019 synergistically sensitizes lung cancer cells to topoisomerase II poisons by multiple mechanisms
Source: Sci Rep. 2024 Apr 16;14:8785. doi: 10.1038/s41598-024-59332-9 (PMC11021496; doi:10.1038/s41598-024-59332-9)

Supplementary Fig.1

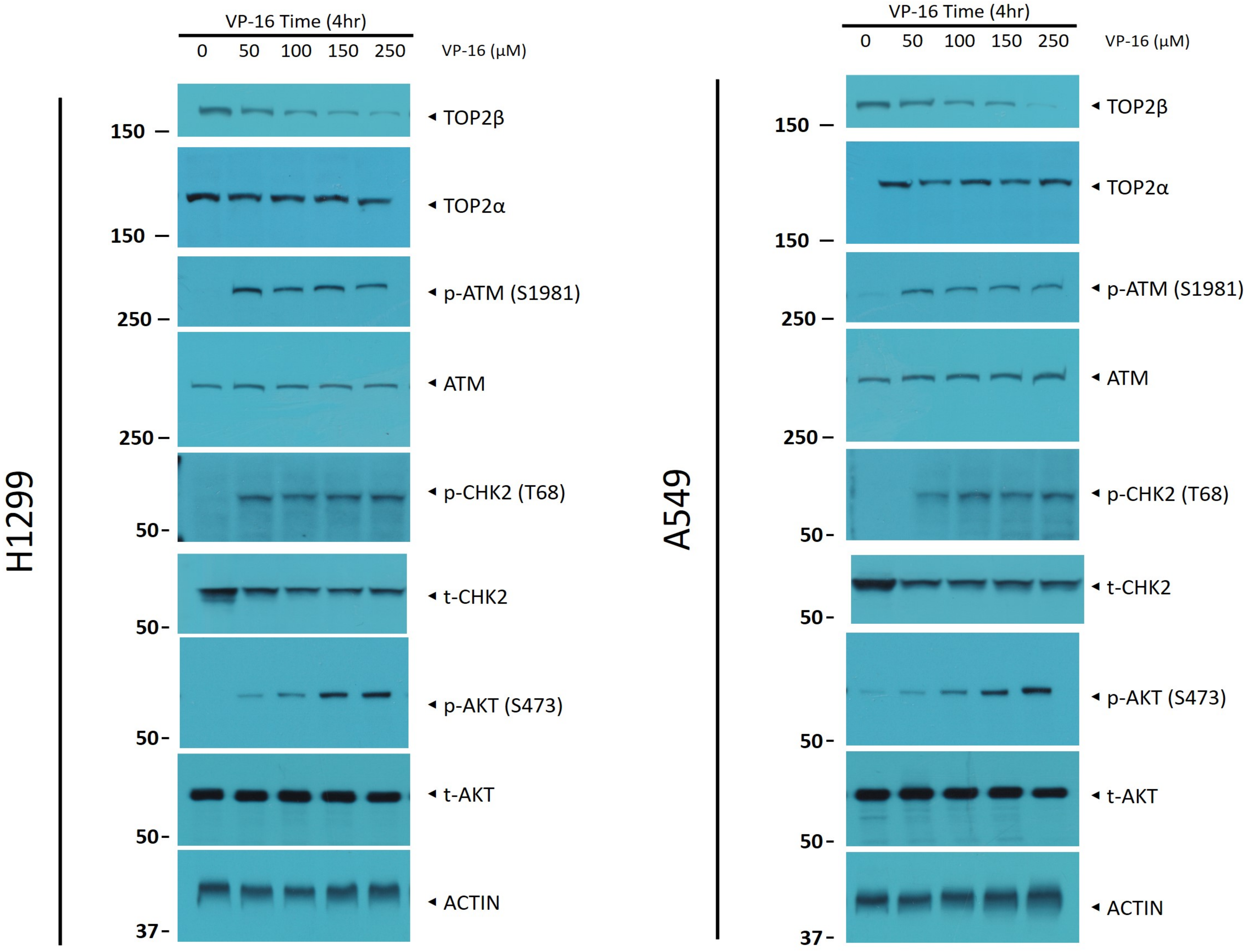

Supplementary Fig.1

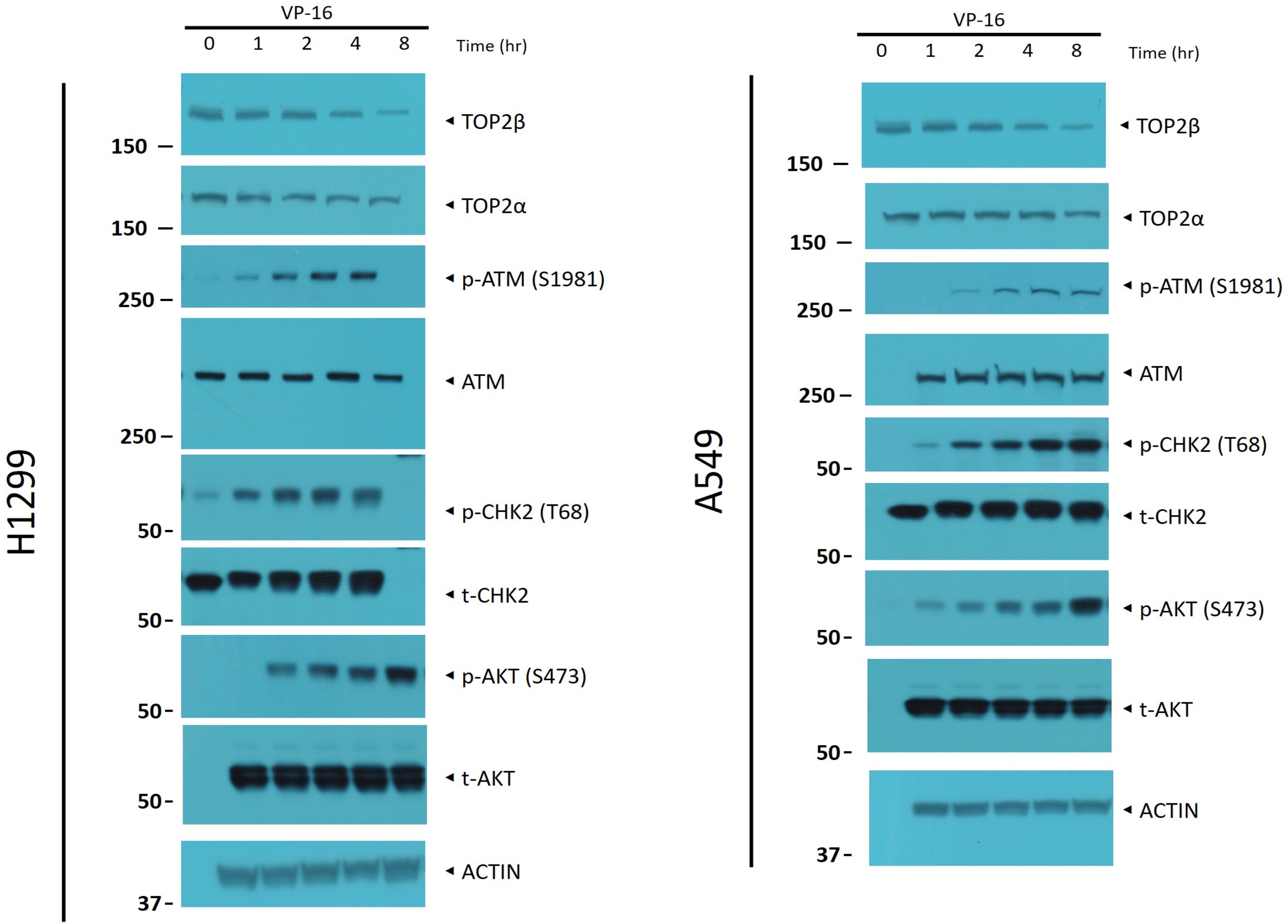

Supplementary Fig.1

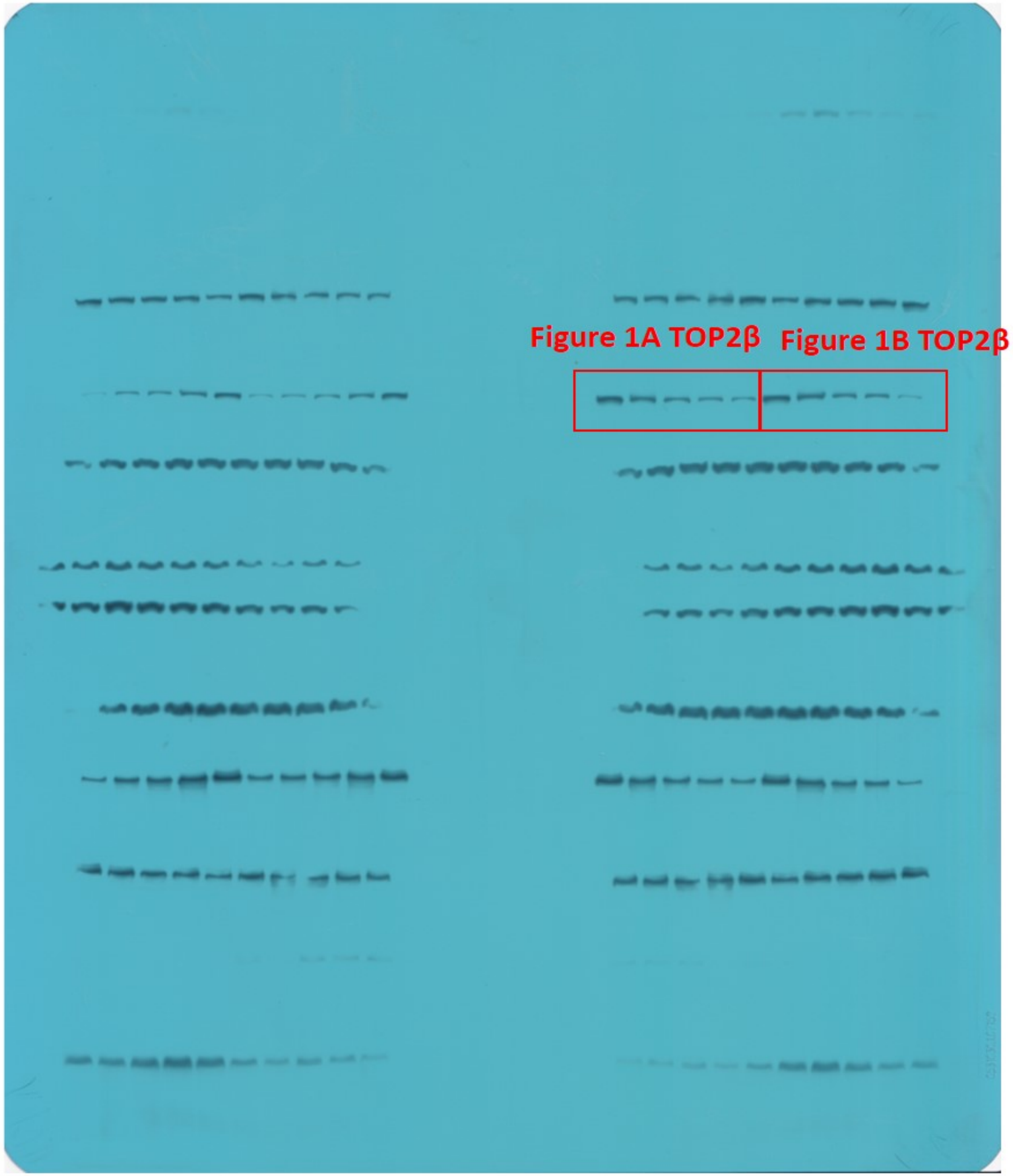

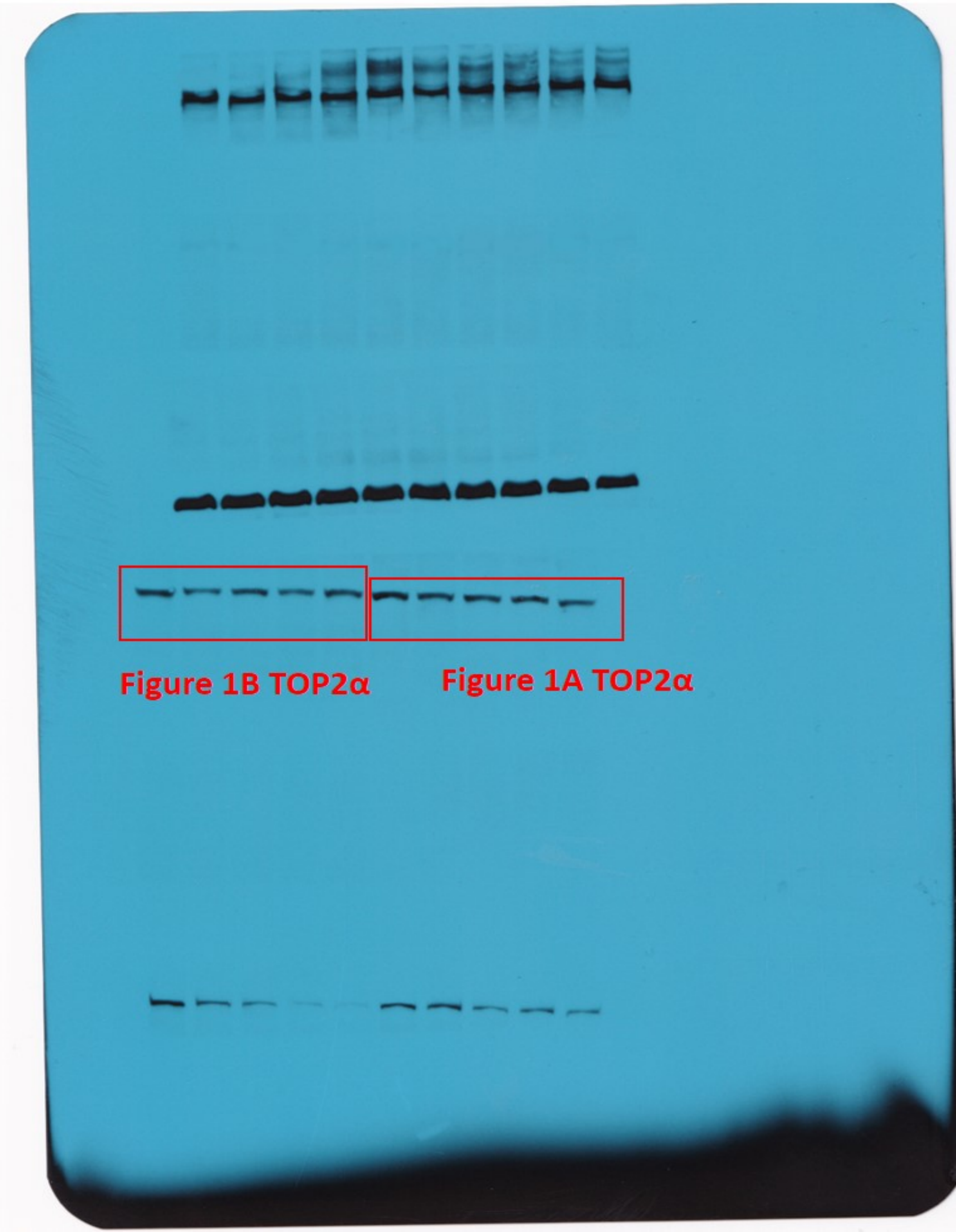

Supplementary Fig.1

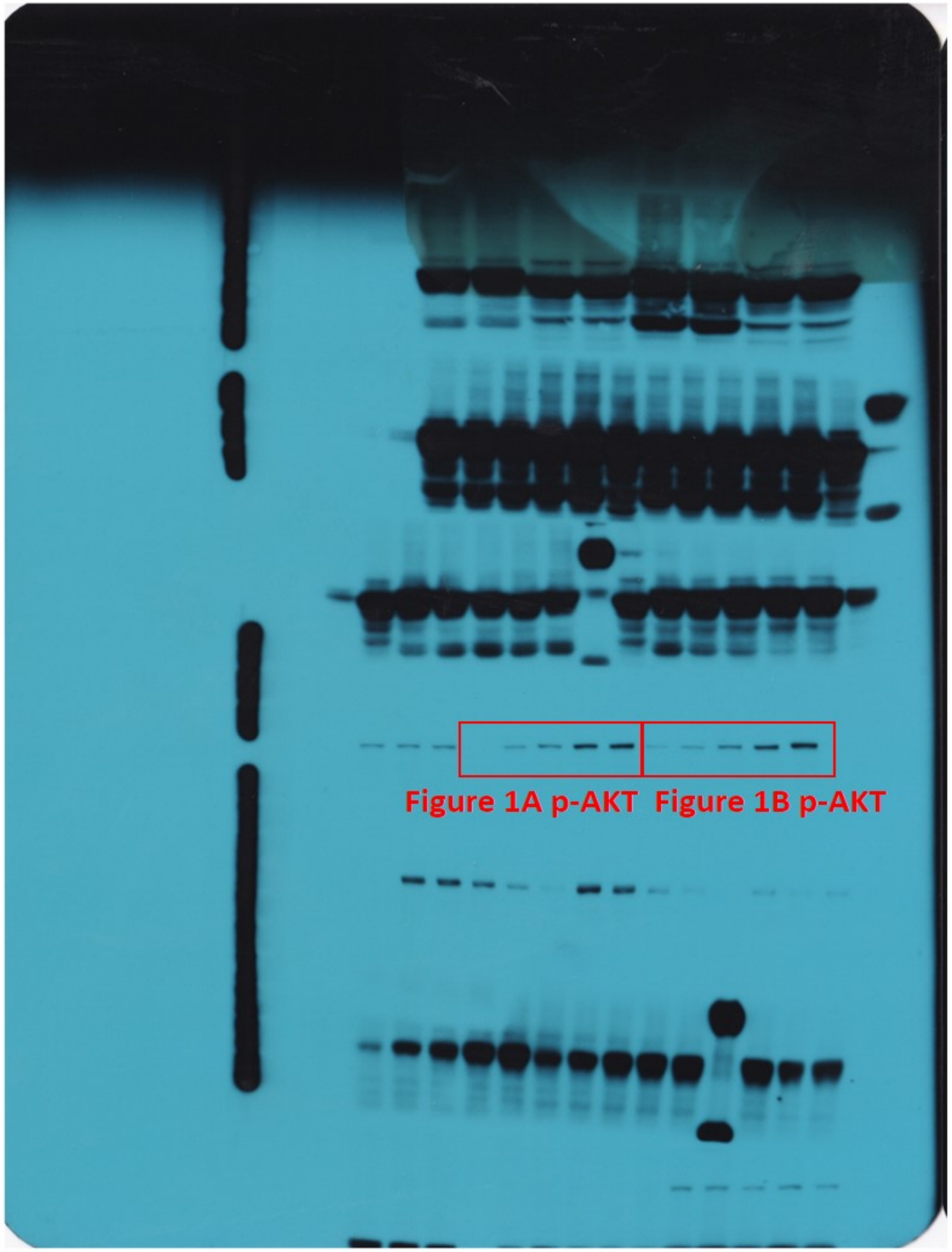

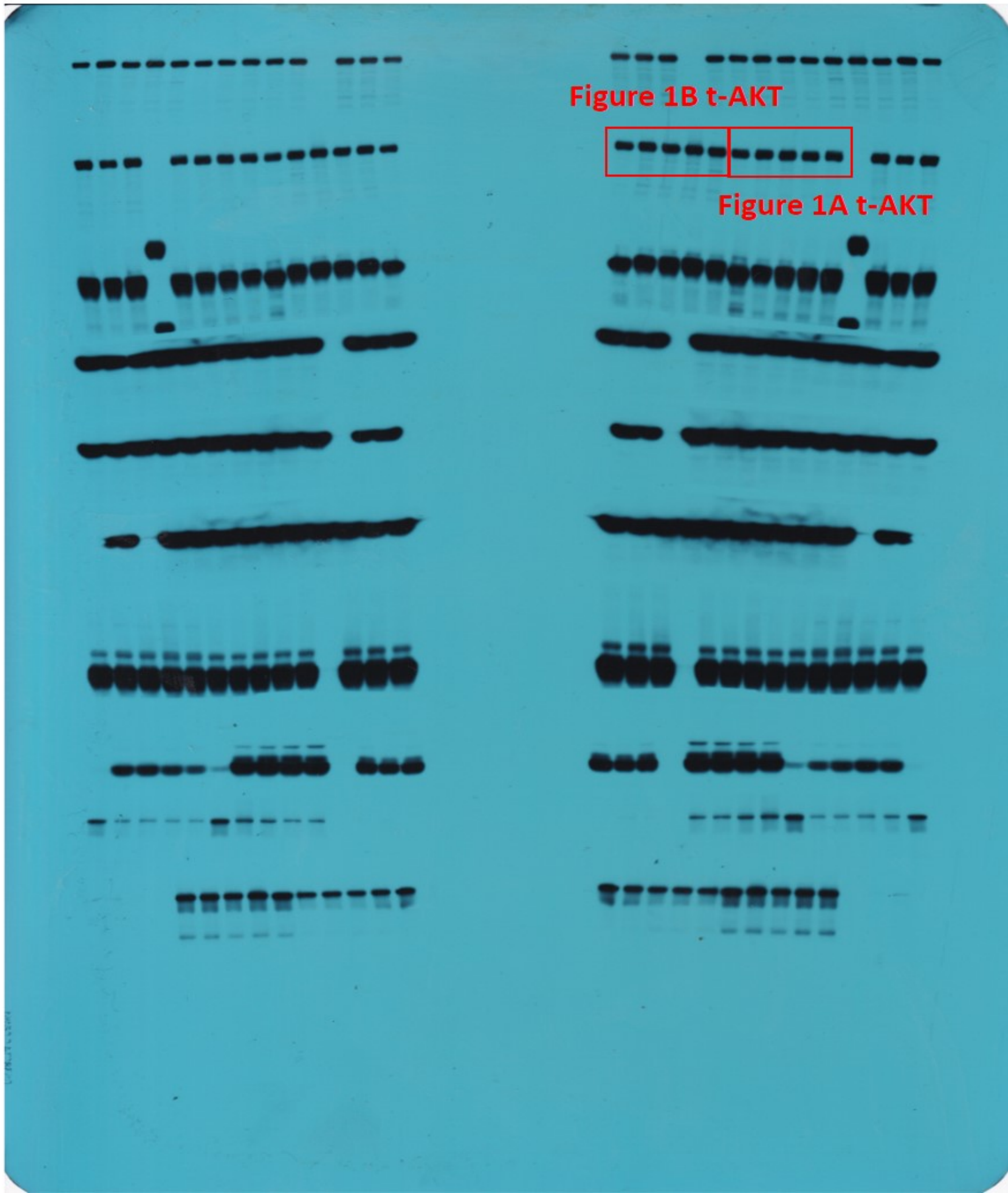

Supplementary Fig.1

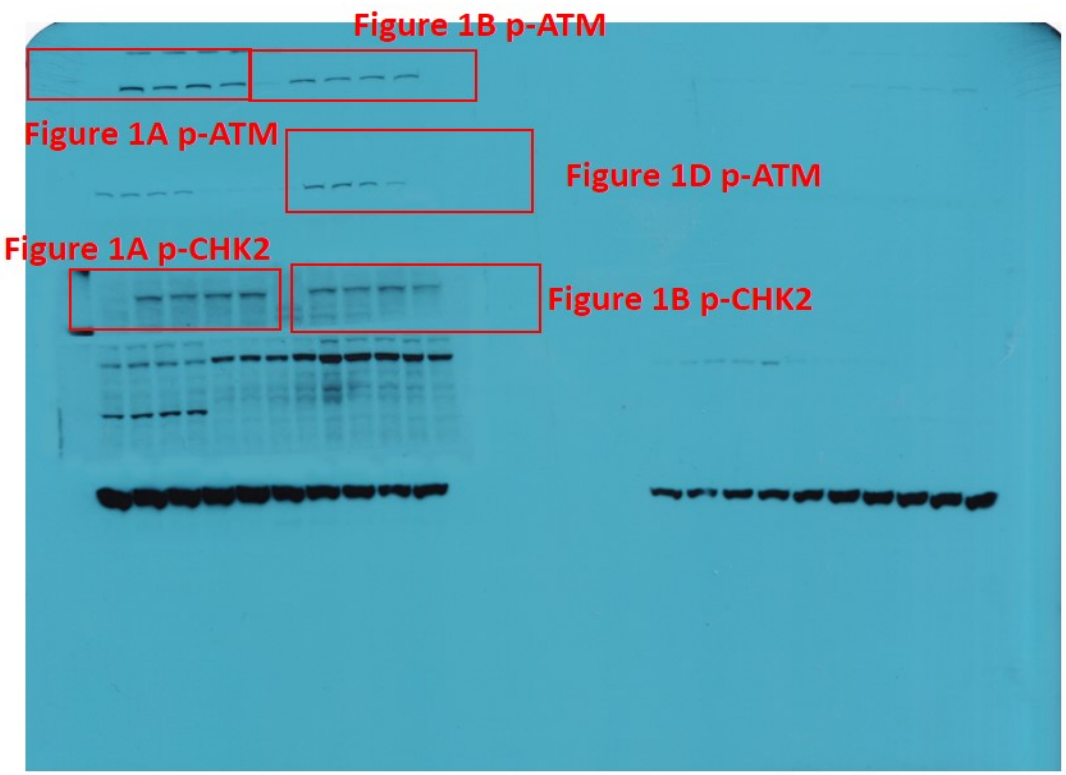

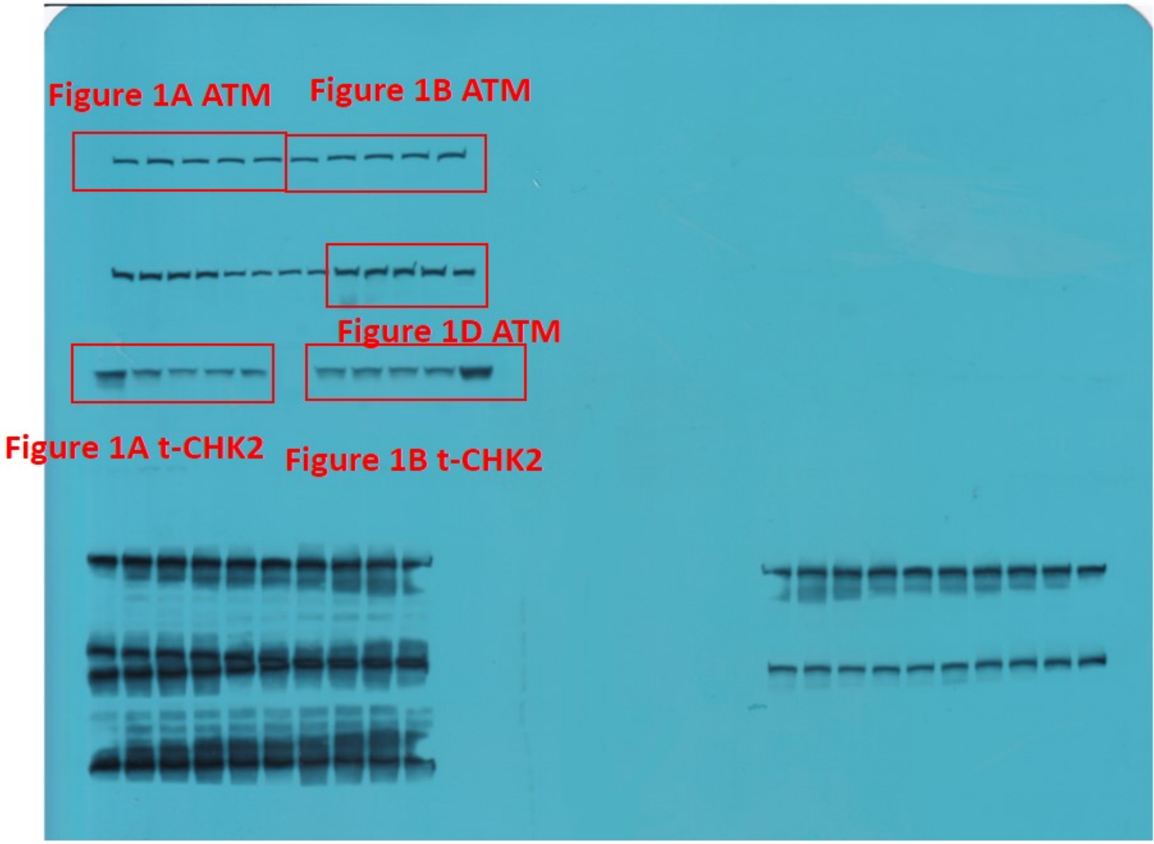

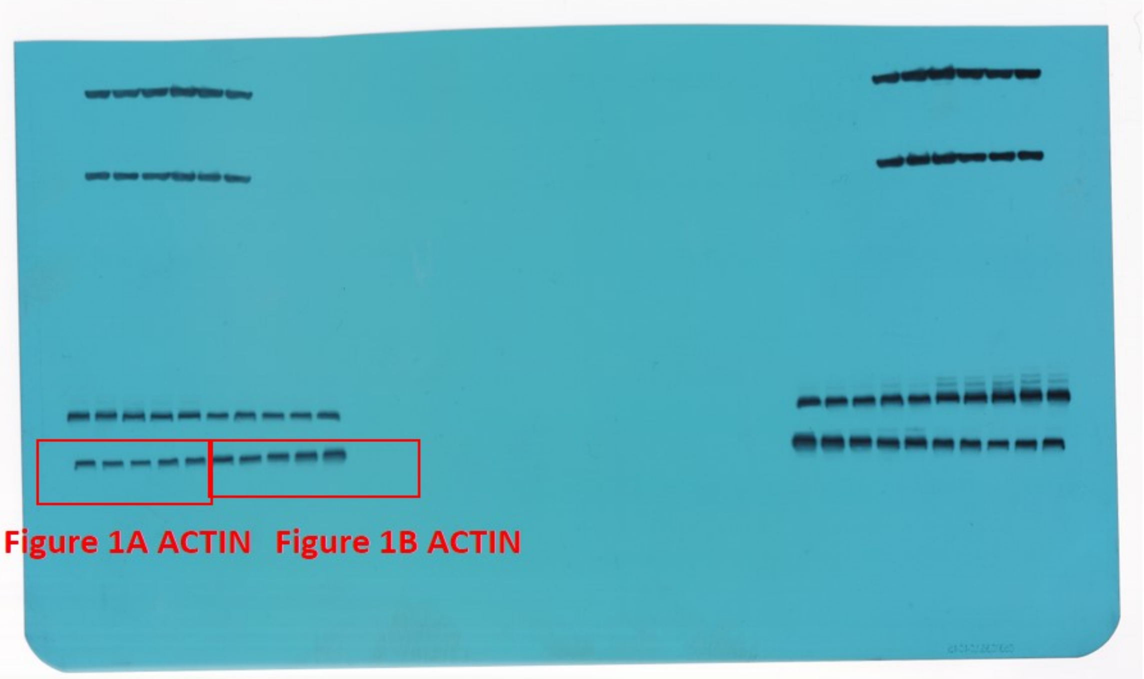

Supplementary Fig.1

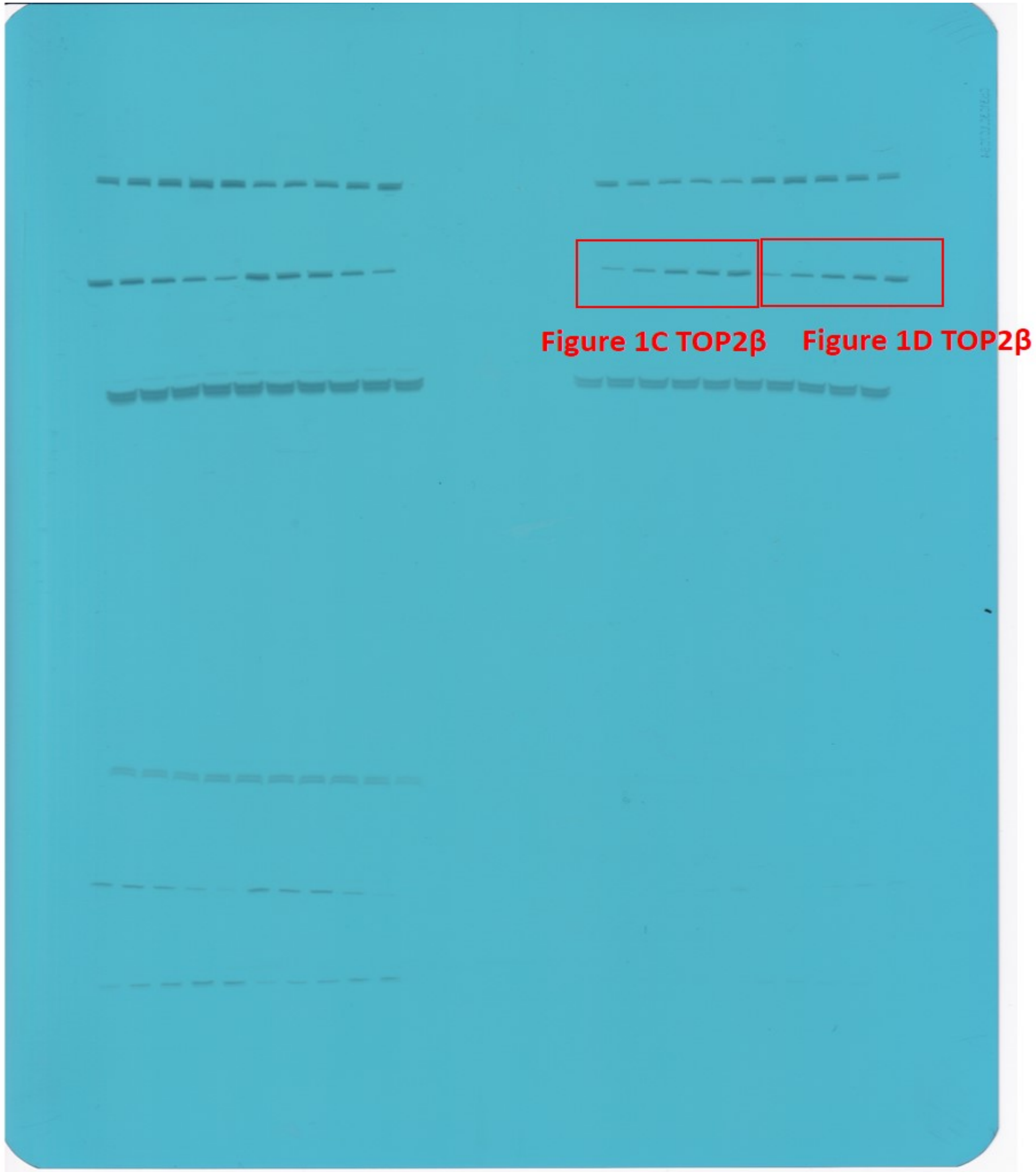

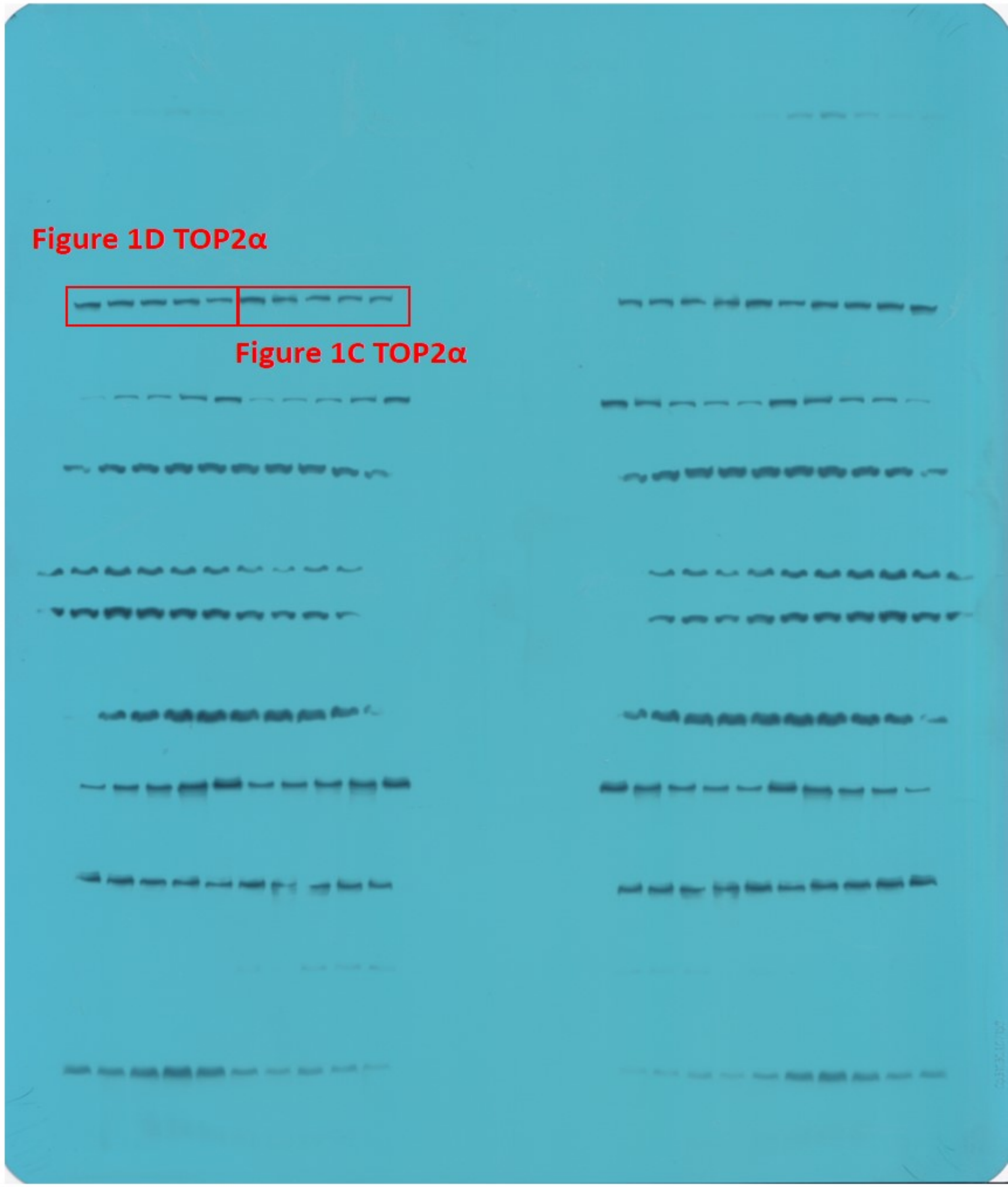

Supplementary Fig.1

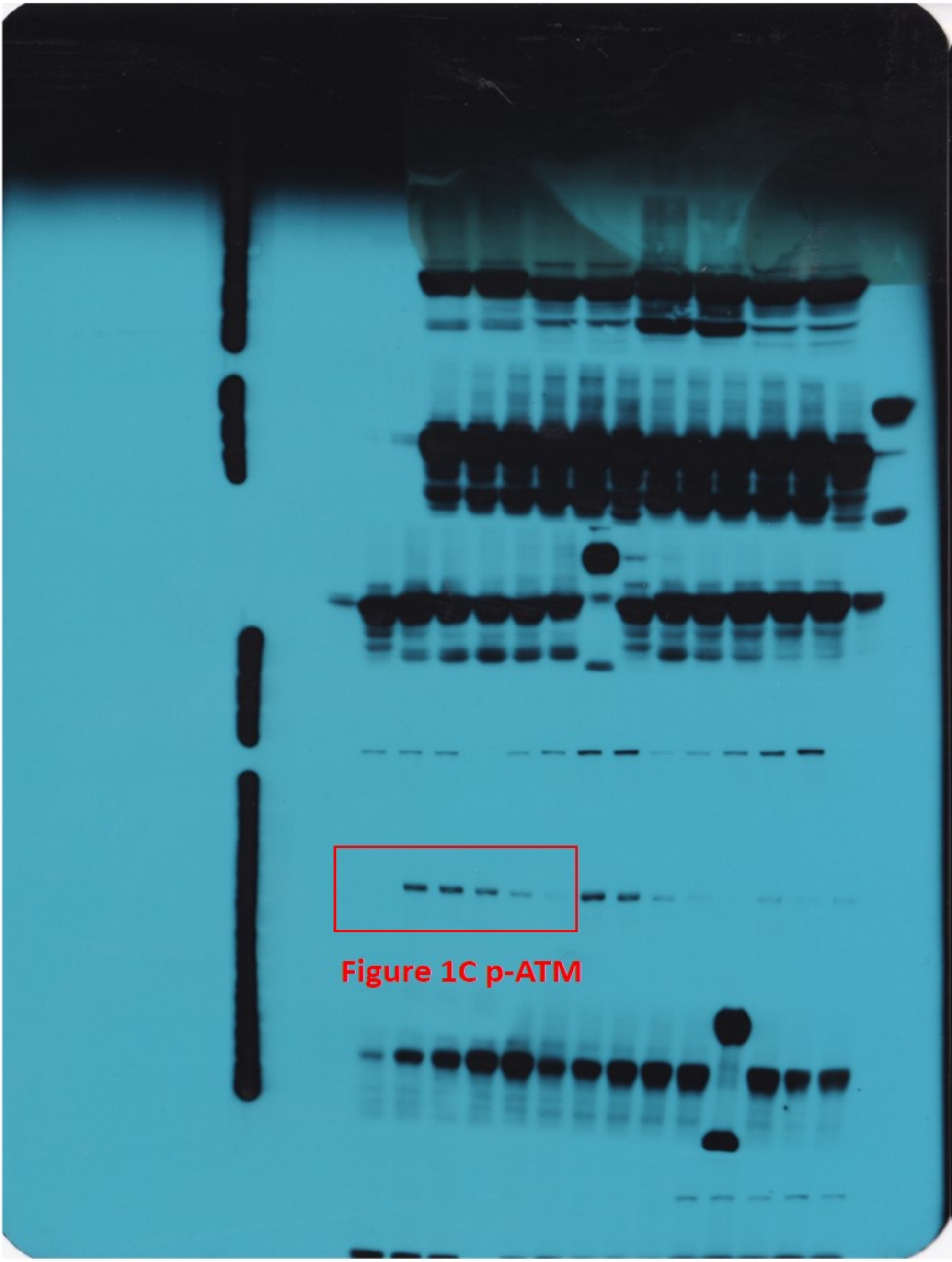

Supplementary Fig.1

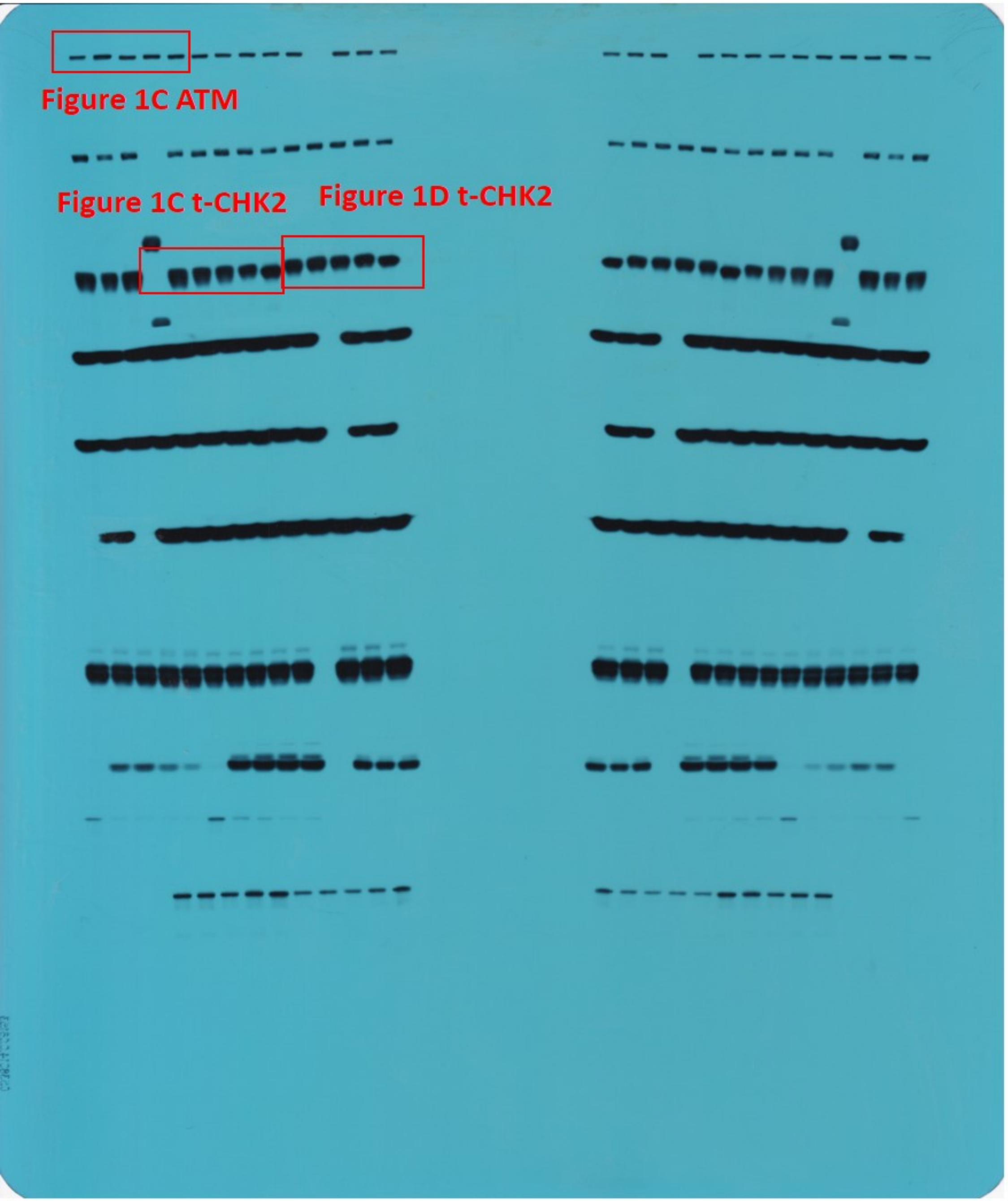

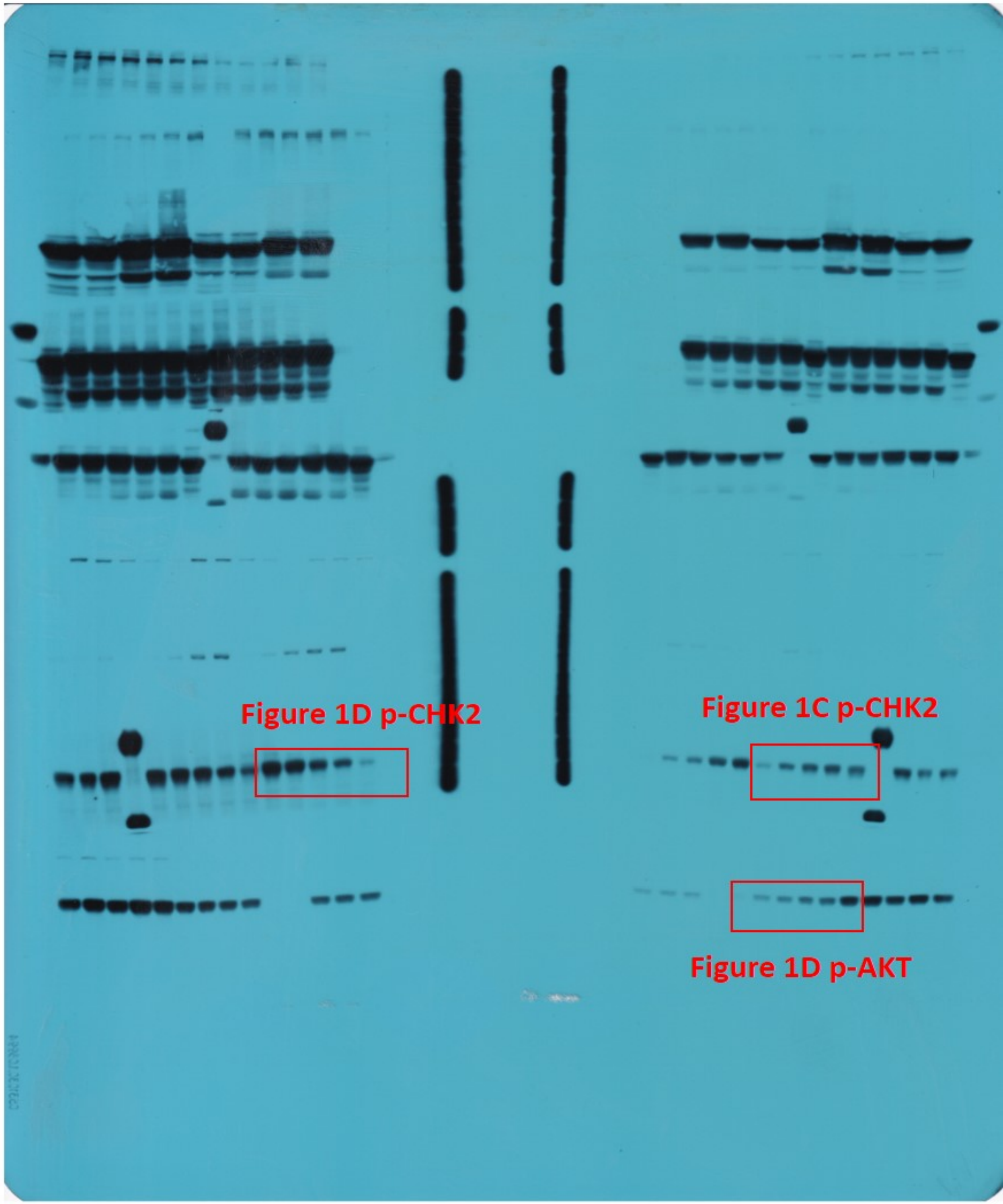

Supplementary Fig.1

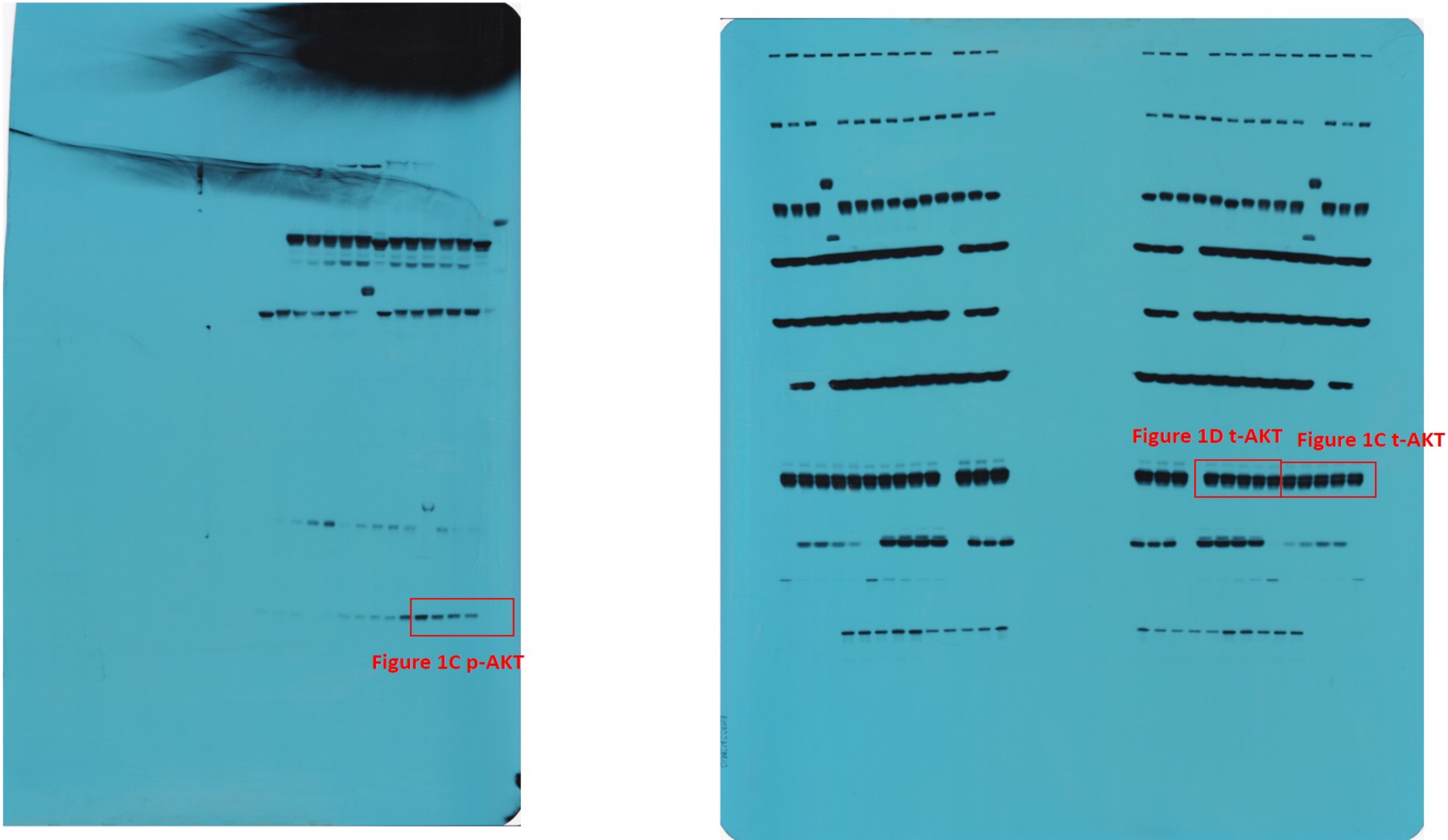

Supplementary Fig.1

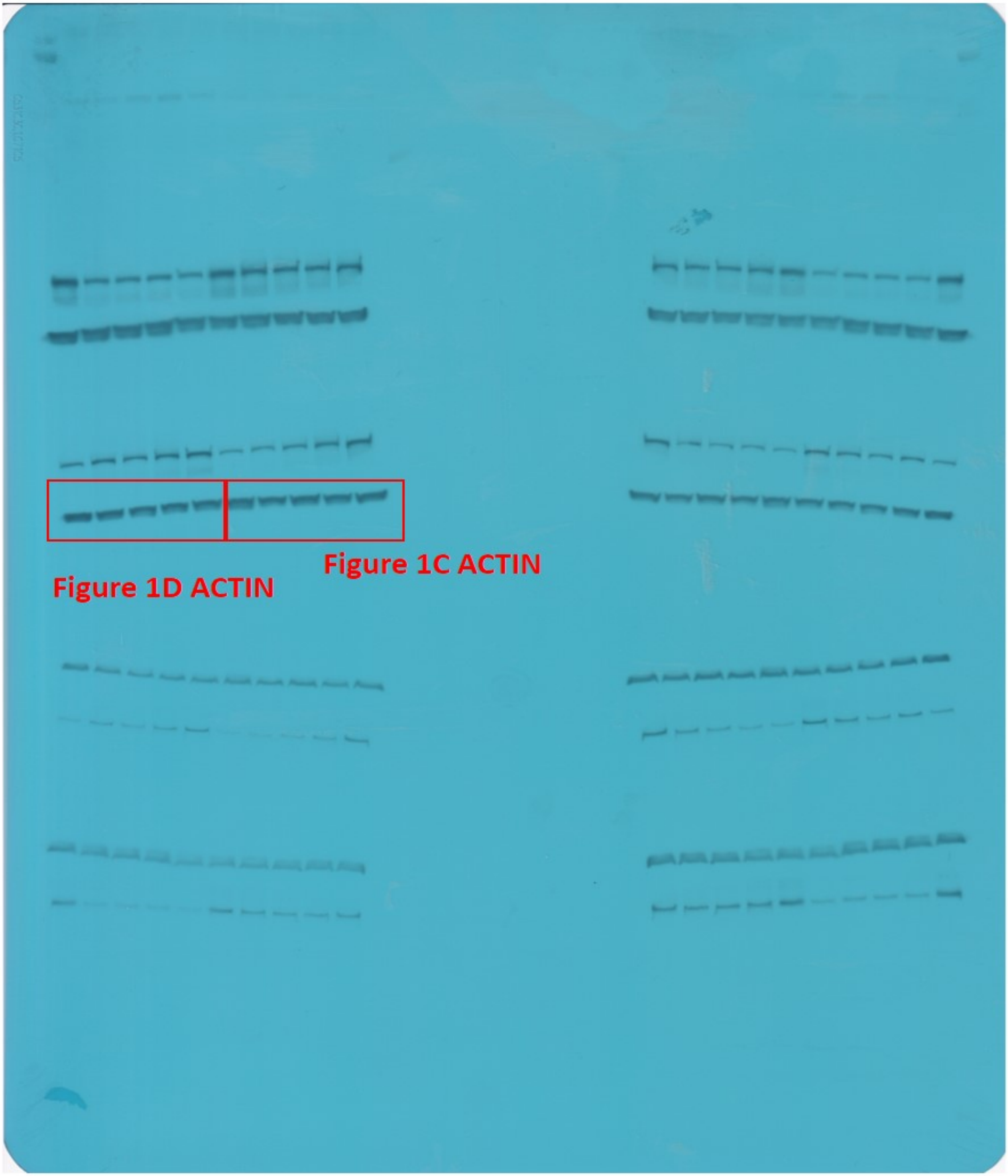

Supplement: Supplementary file 1 — Supplementary Information 1. [file 41598_2024_59332_MOESM1_ESM.pdf]
