# Supplementary material for: Author Correction: ATM inhibitor KU60019 synergistically sensitizes lung cancer cells to topoisomerase II poisons by multiple mechanisms
Source: Sci Rep. 2024 Apr 16;14:8785. doi: 10.1038/s41598-024-59332-9 (PMC11021496; doi:10.1038/s41598-024-59332-9)

Supplementary Fig.2

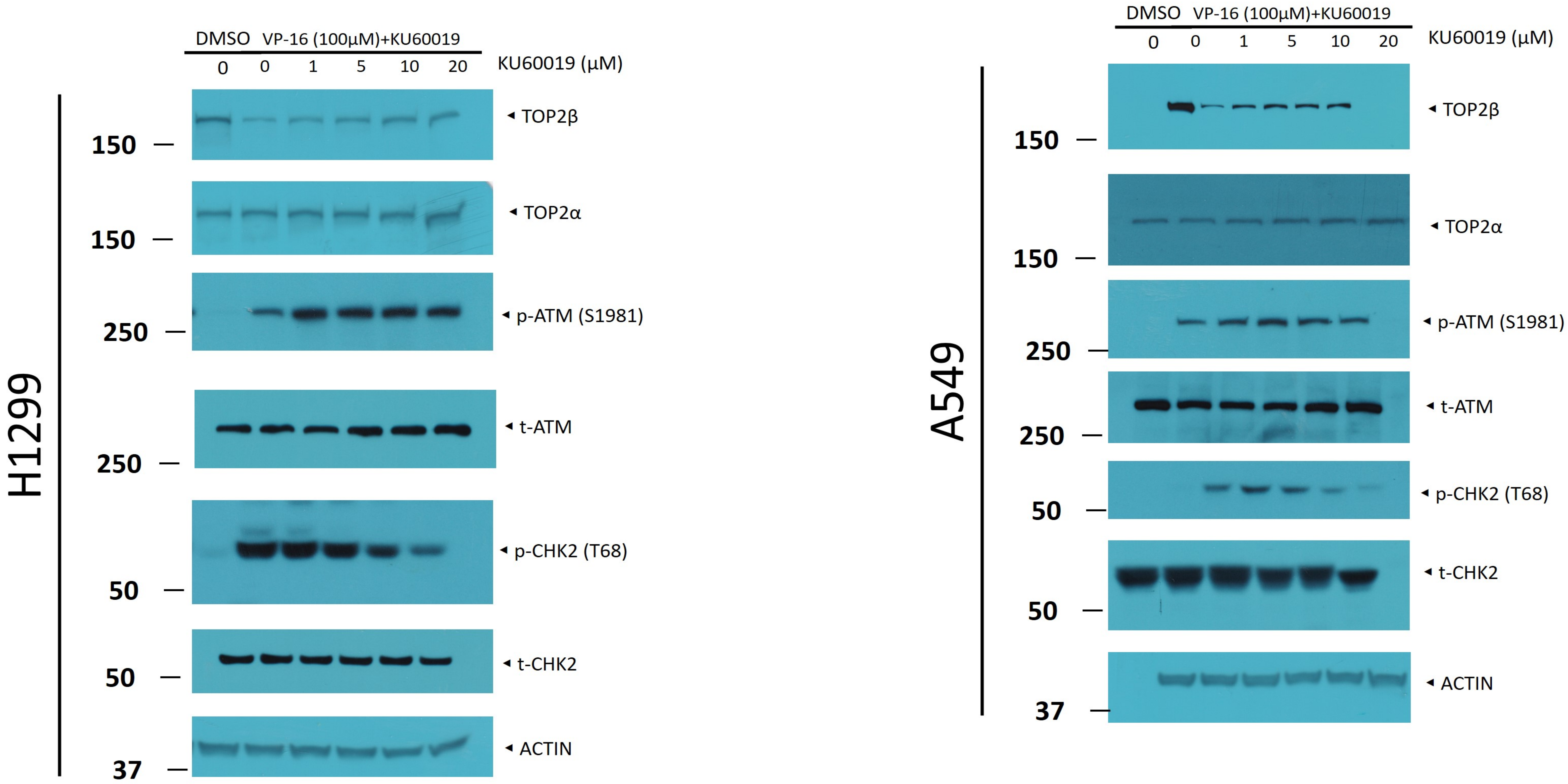

Supplementary Fig.2

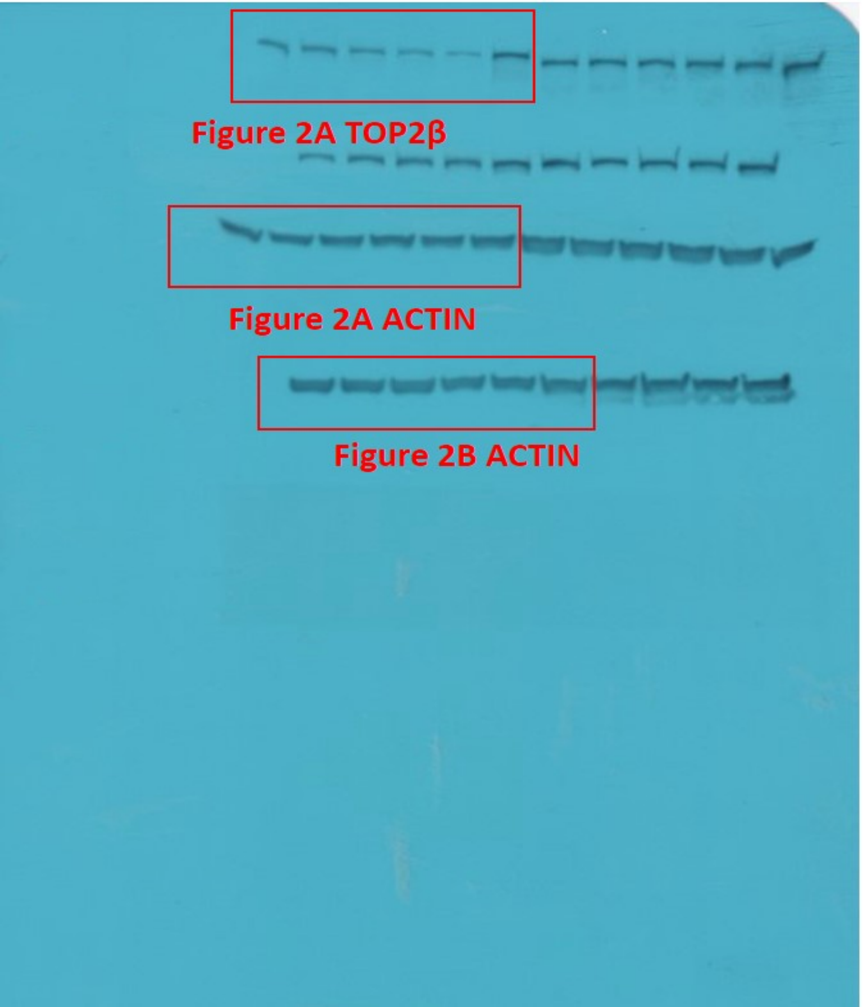

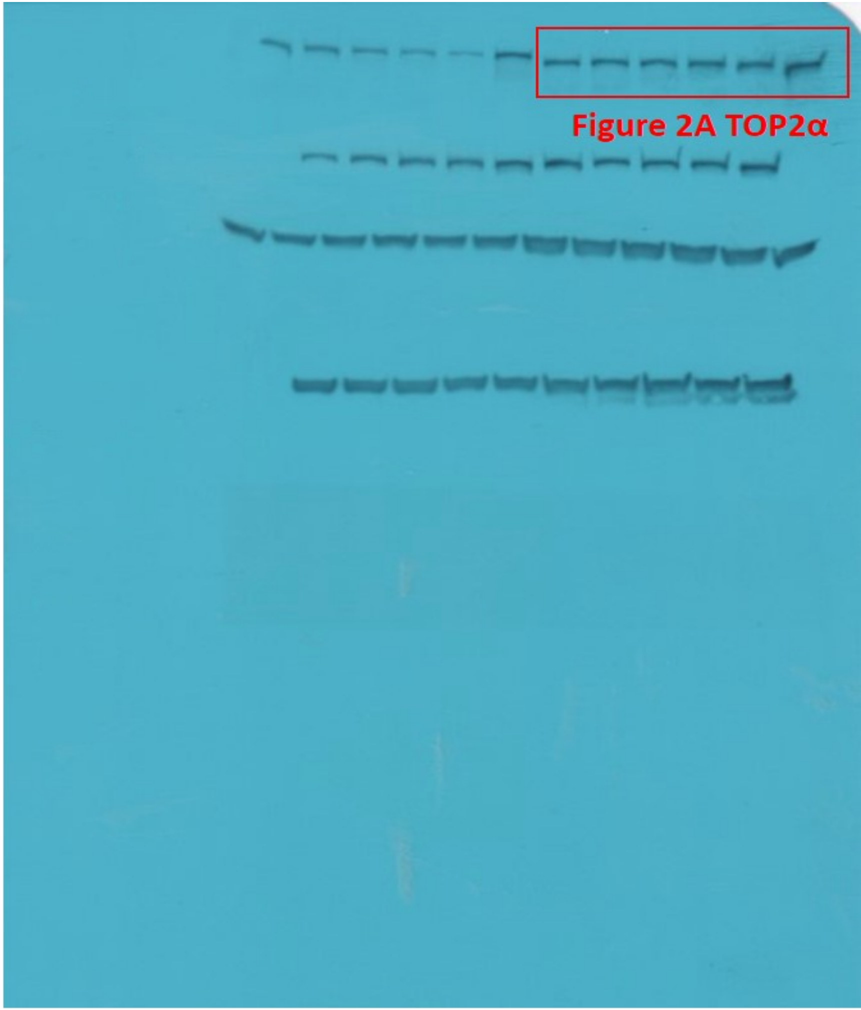

Supplementary Fig.2

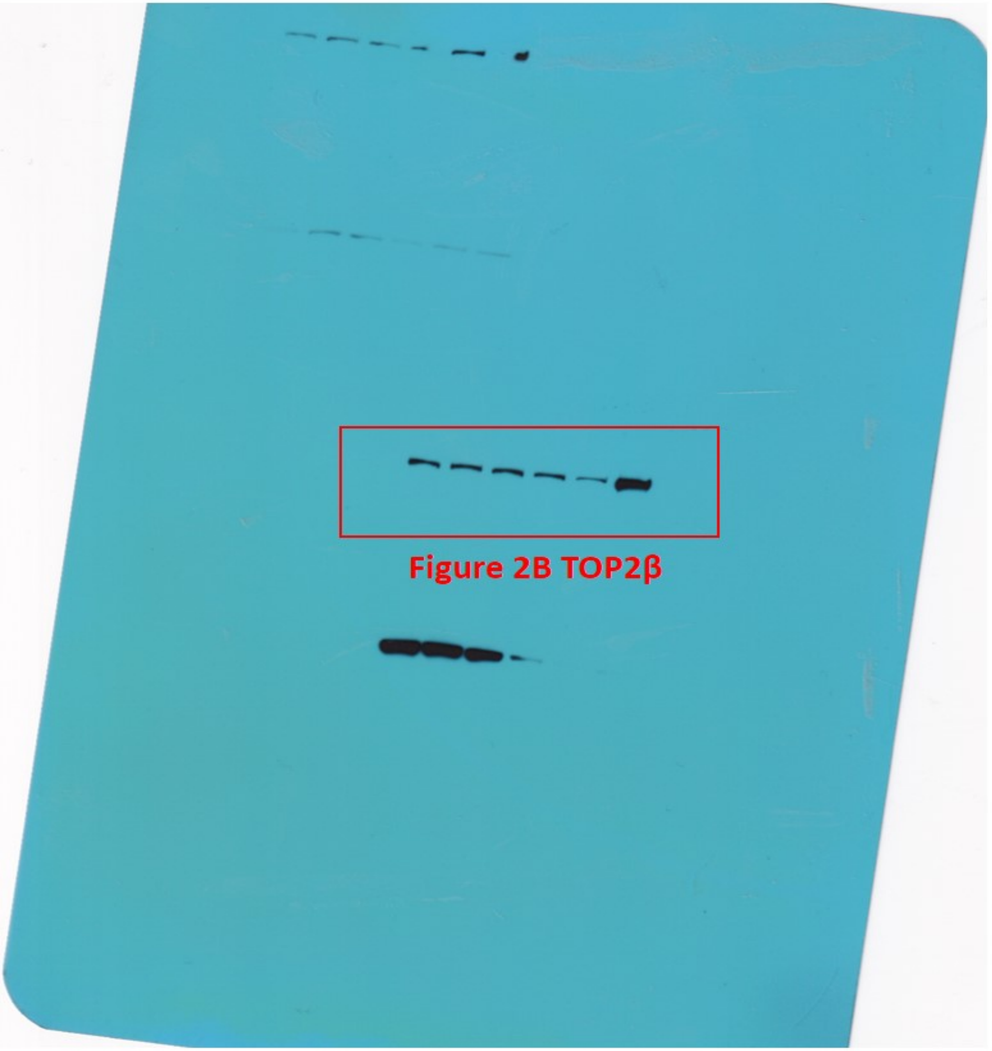

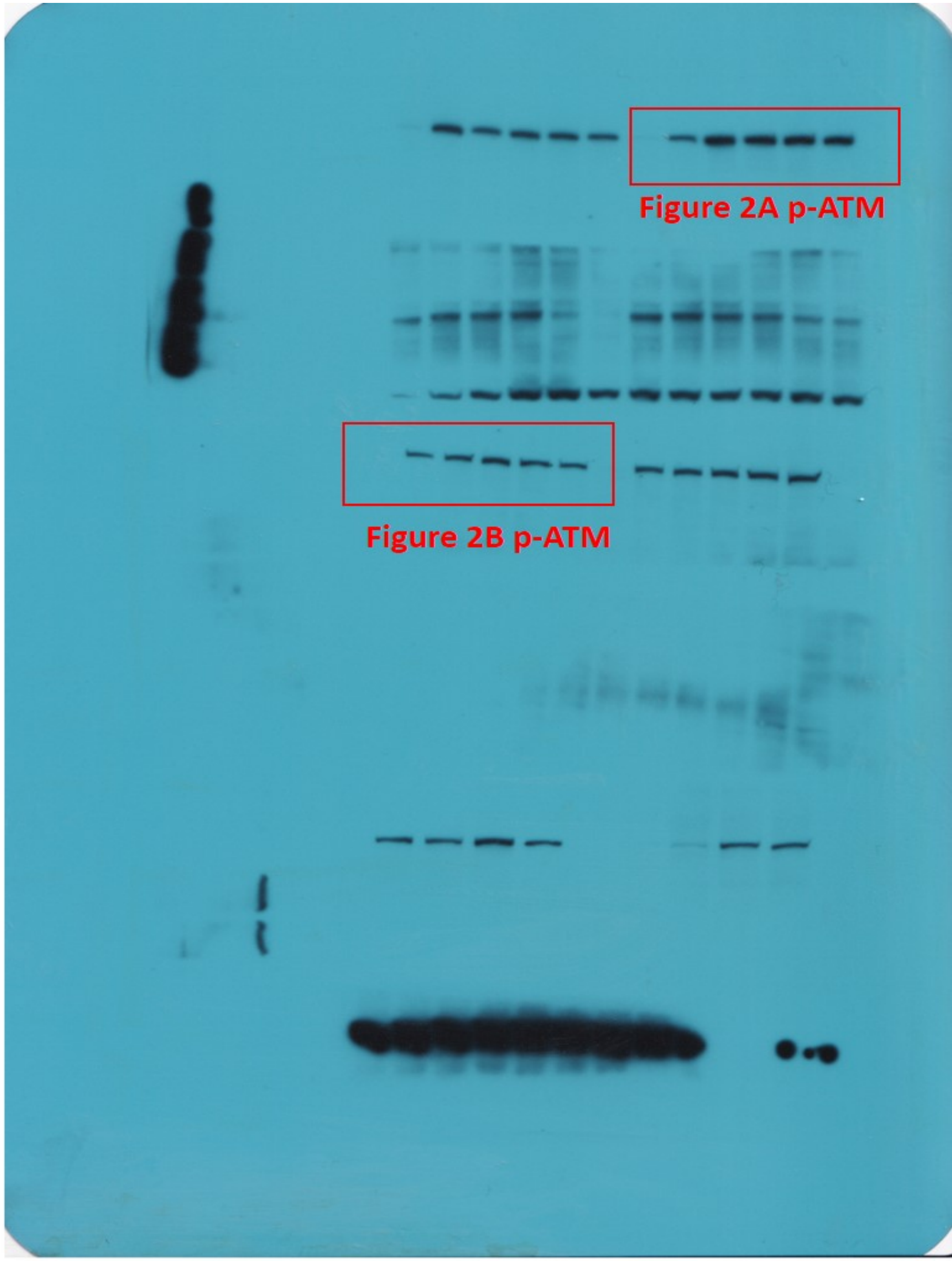

Supplementary Fig.2

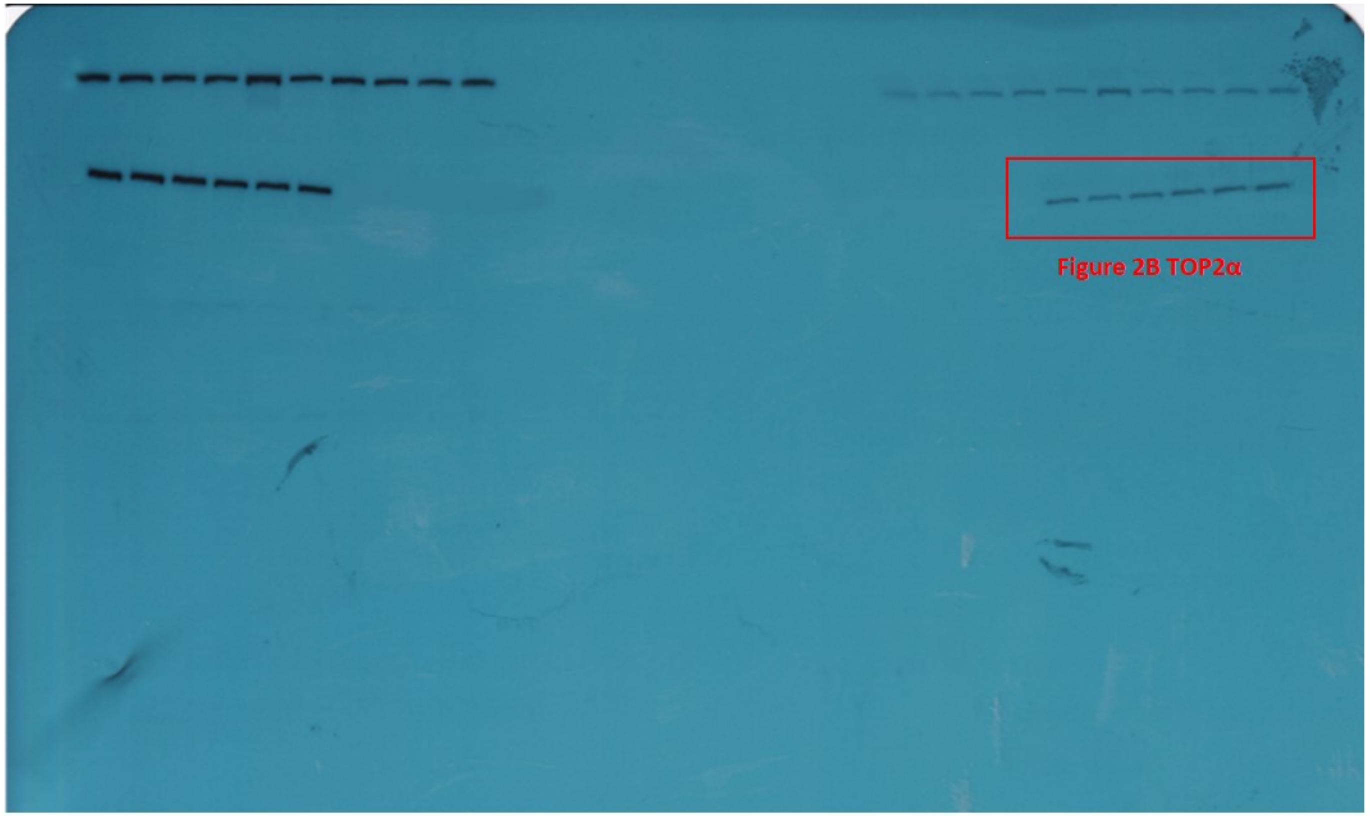

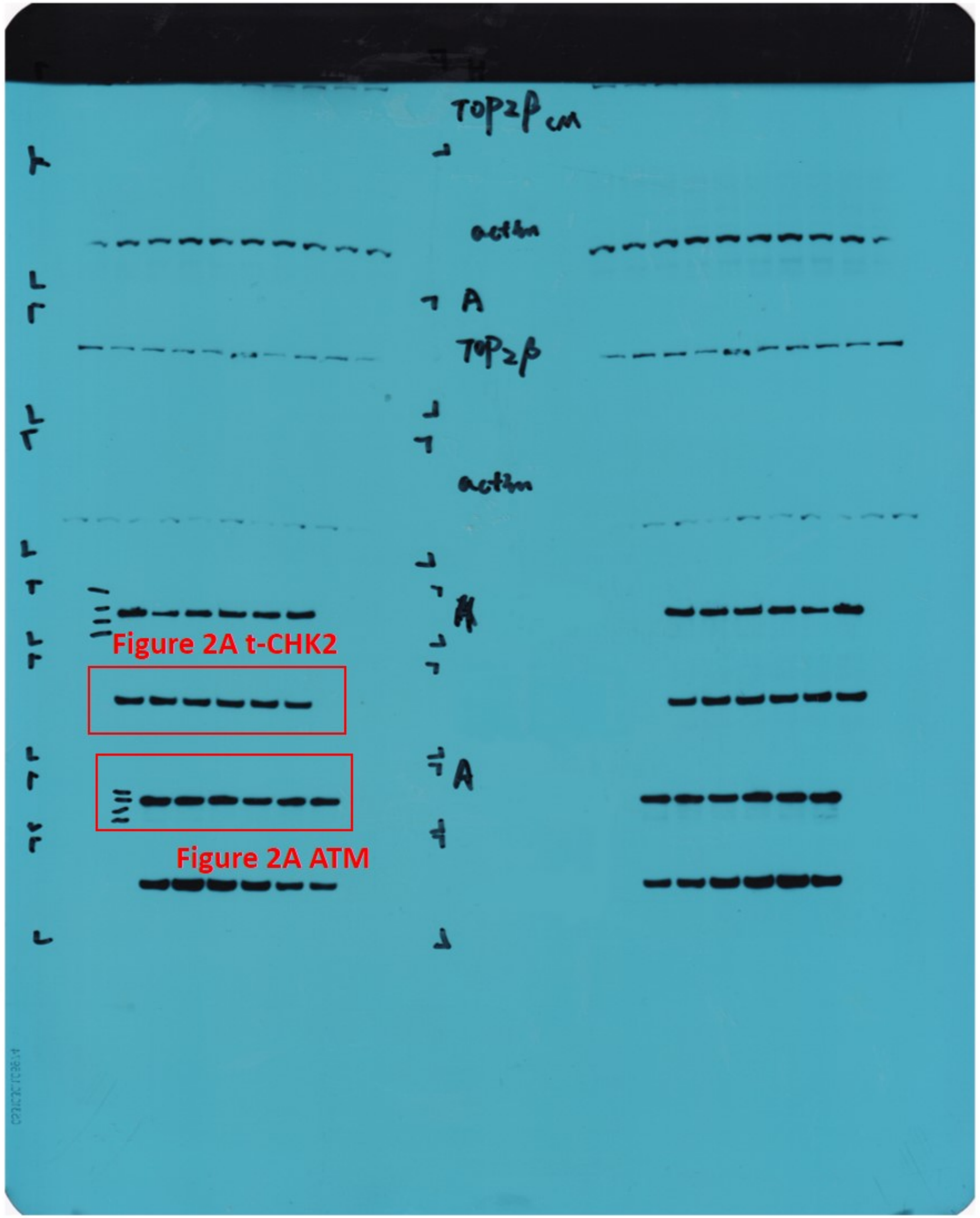

Supplementary Fig.2

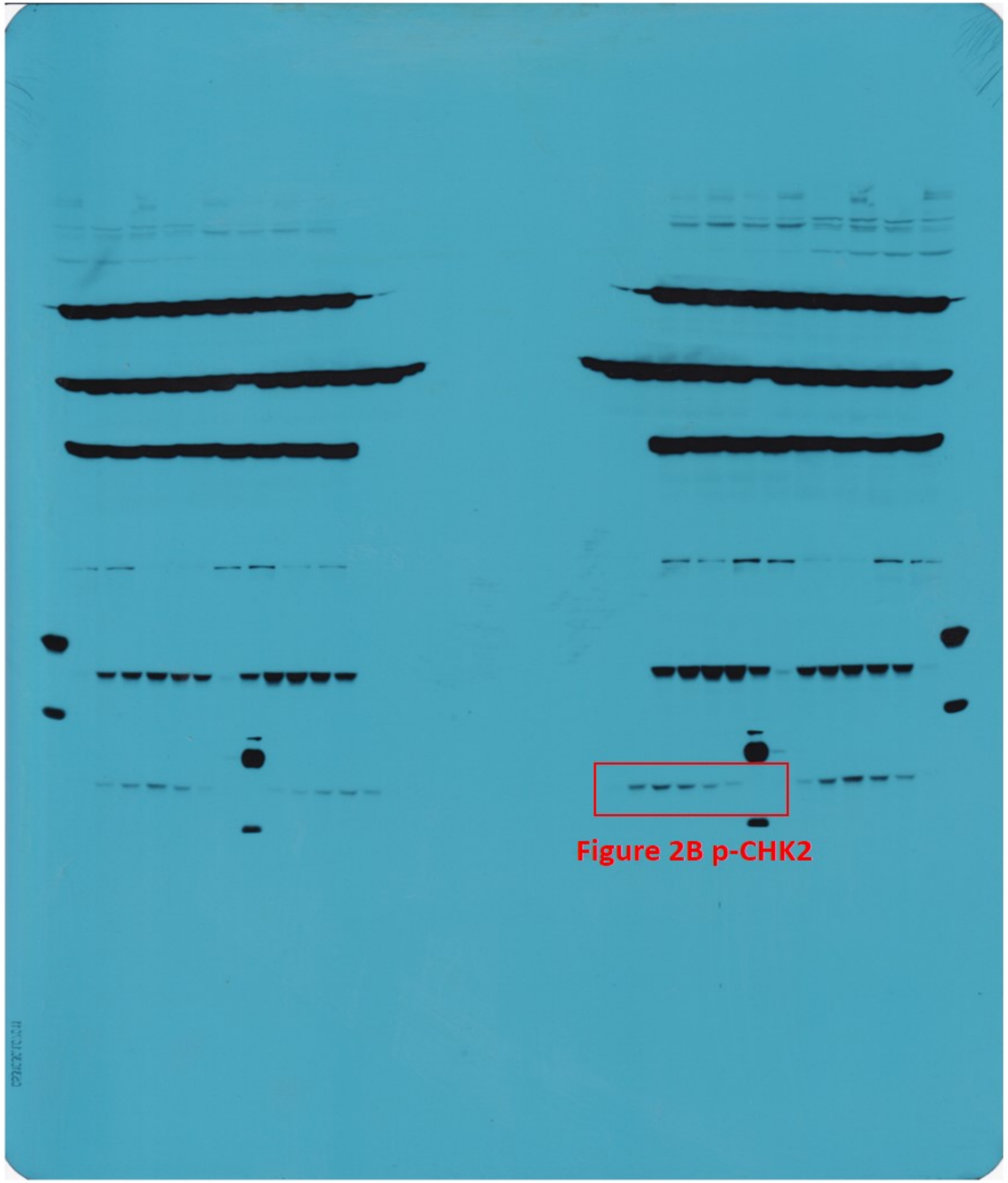

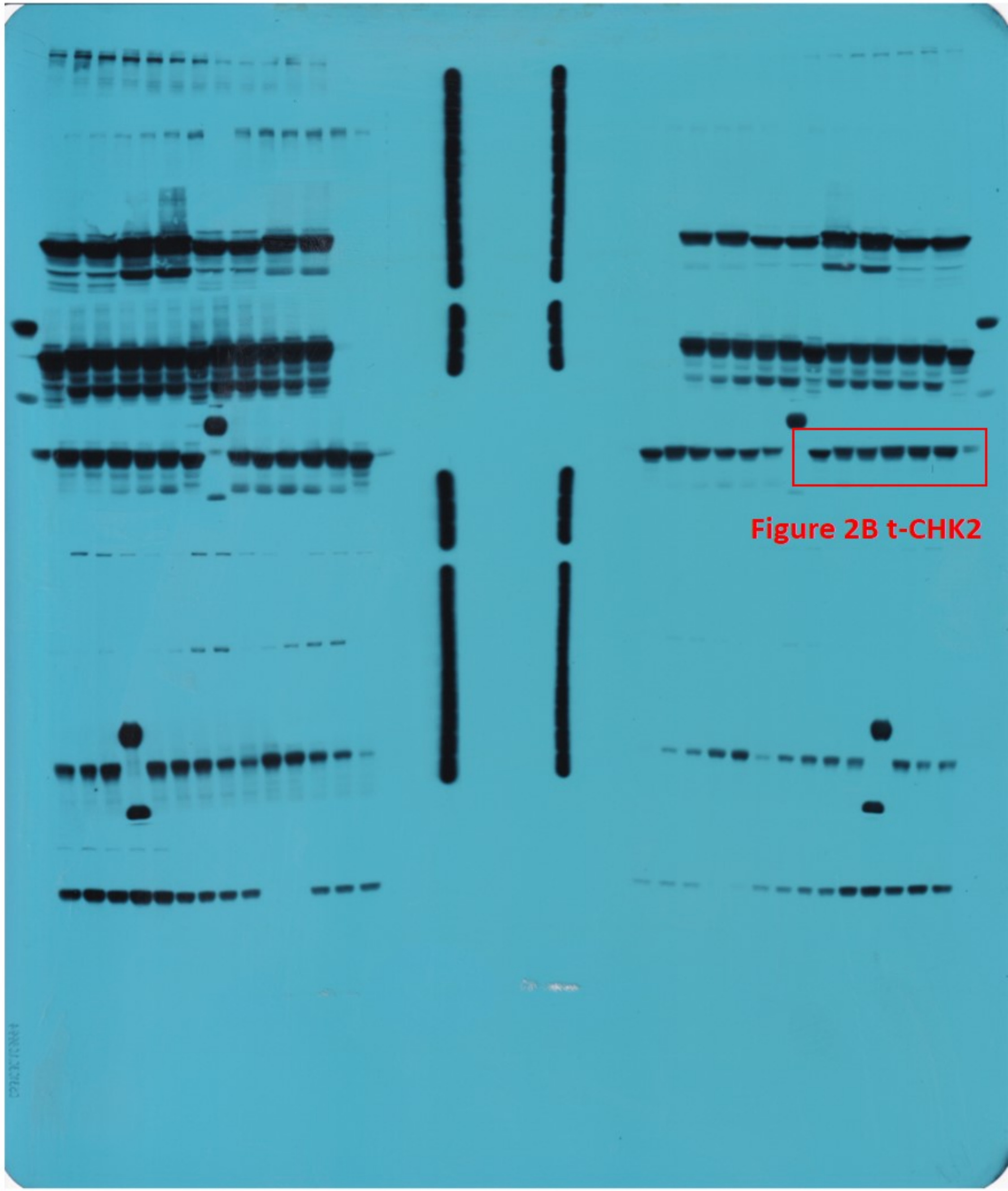

Supplementary Fig.2

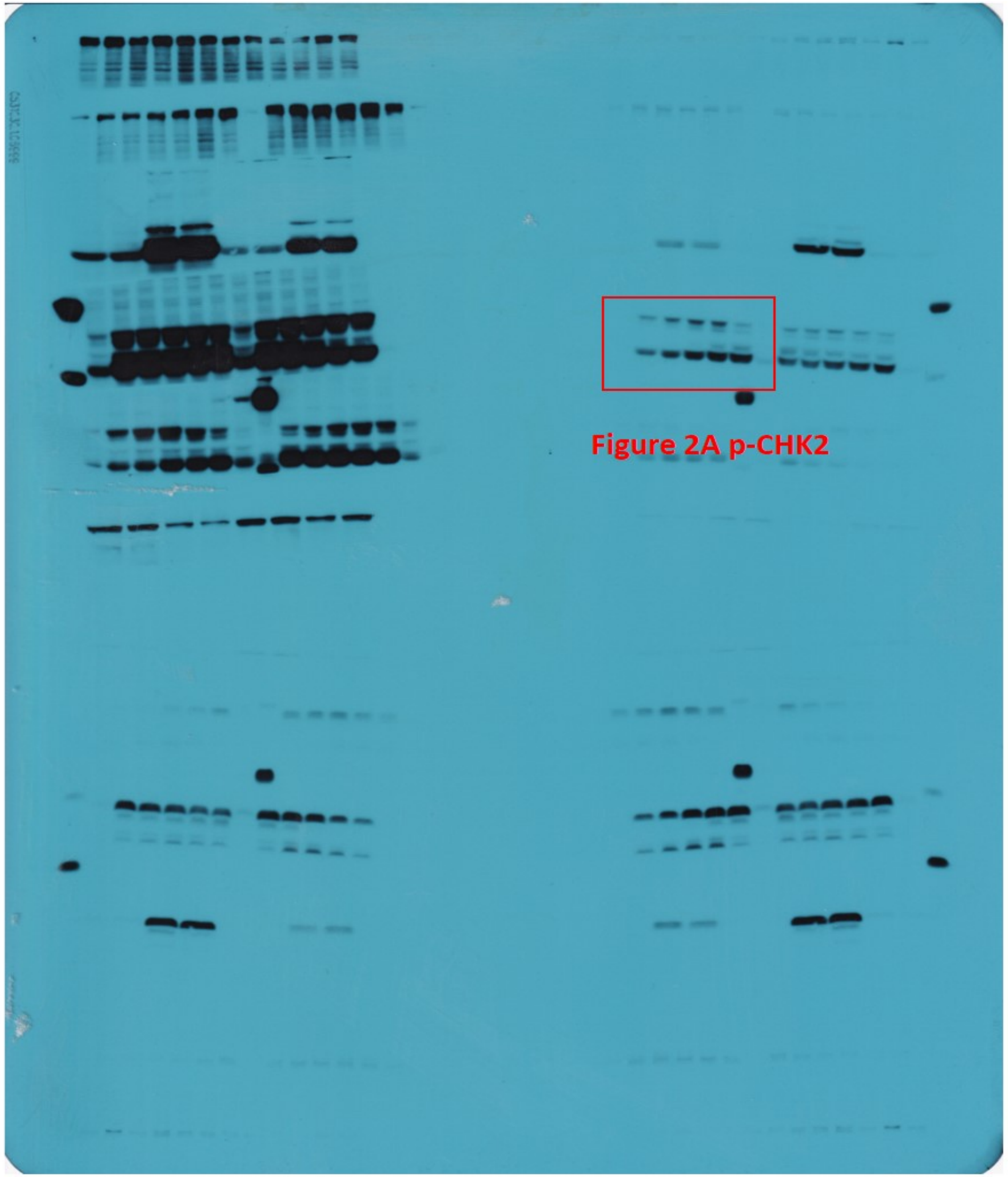

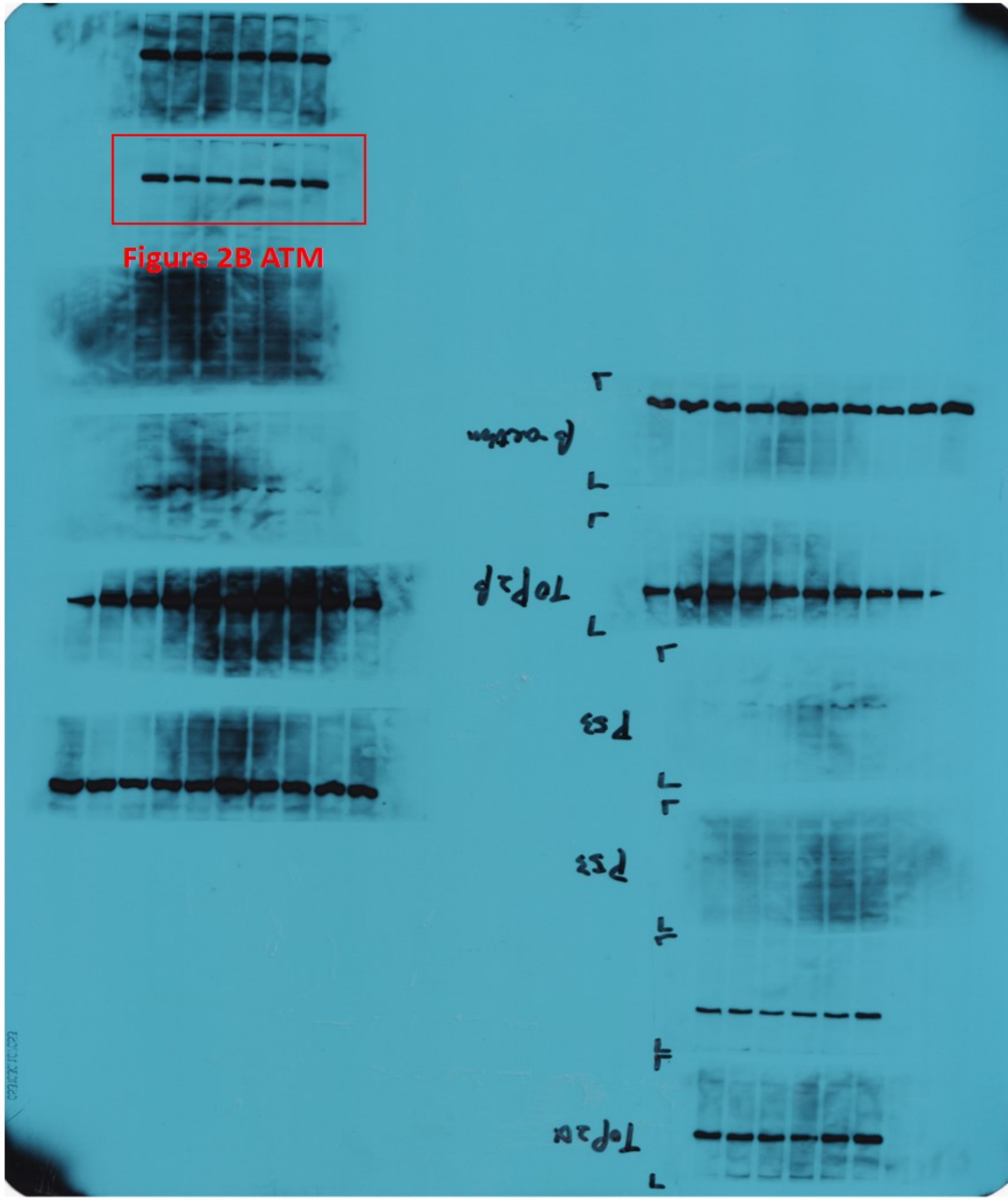

Supplementary Fig.2

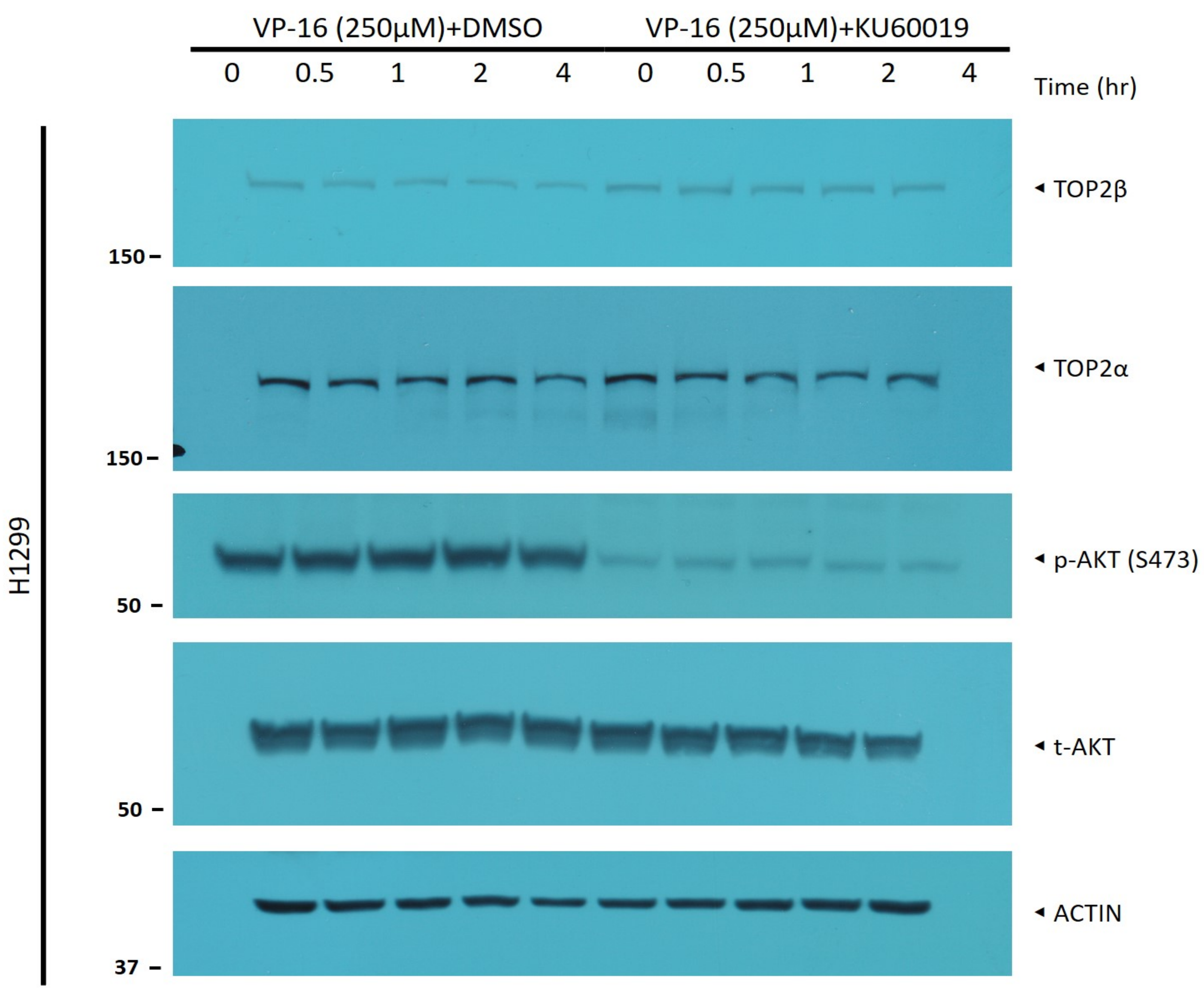

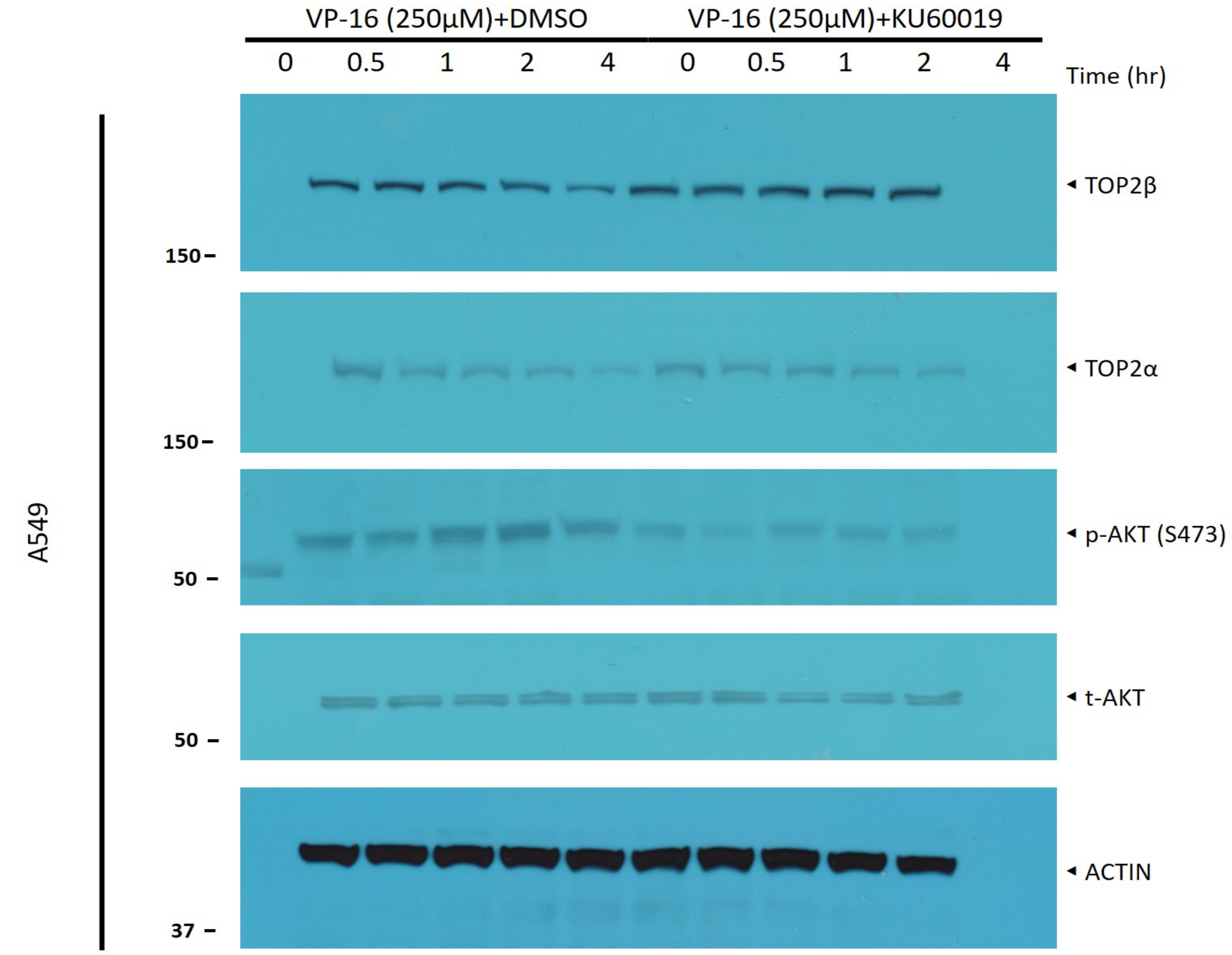

Supplementary Fig.2

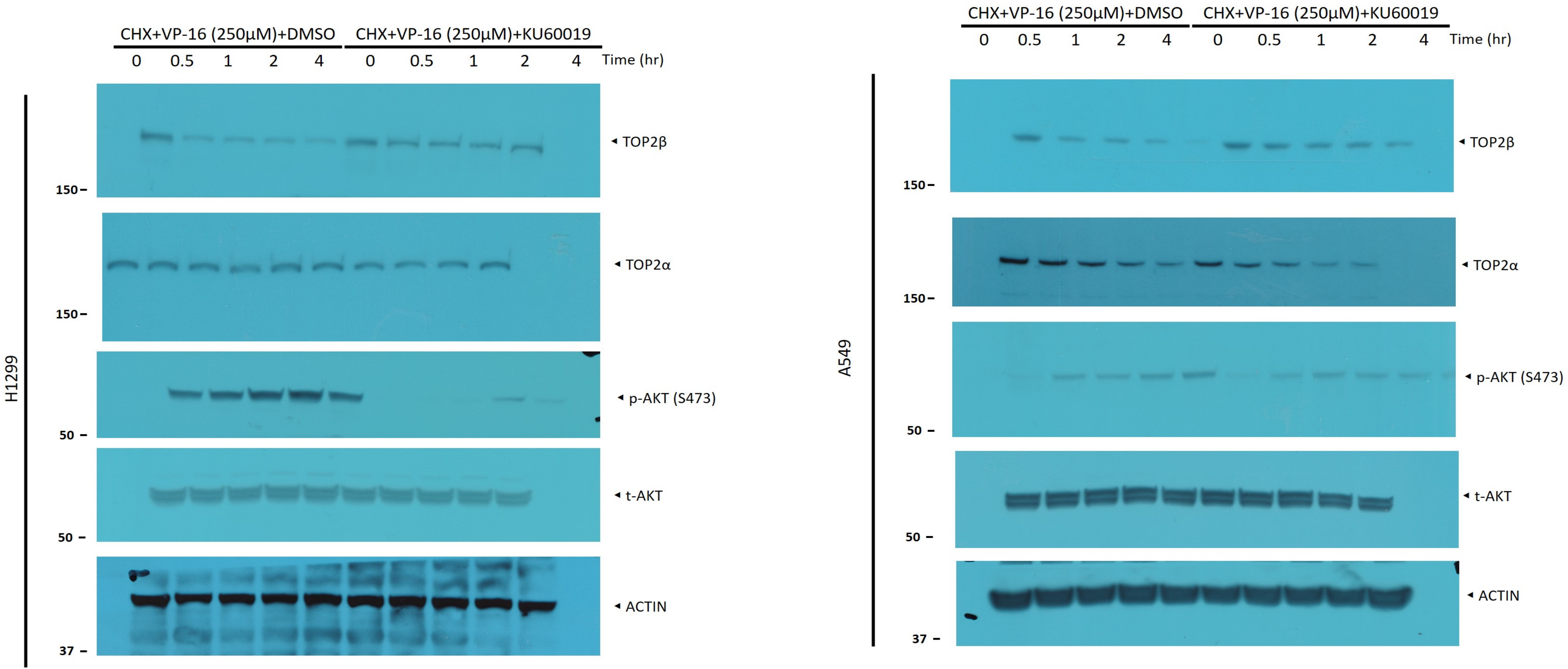

Supplementary Fig.2

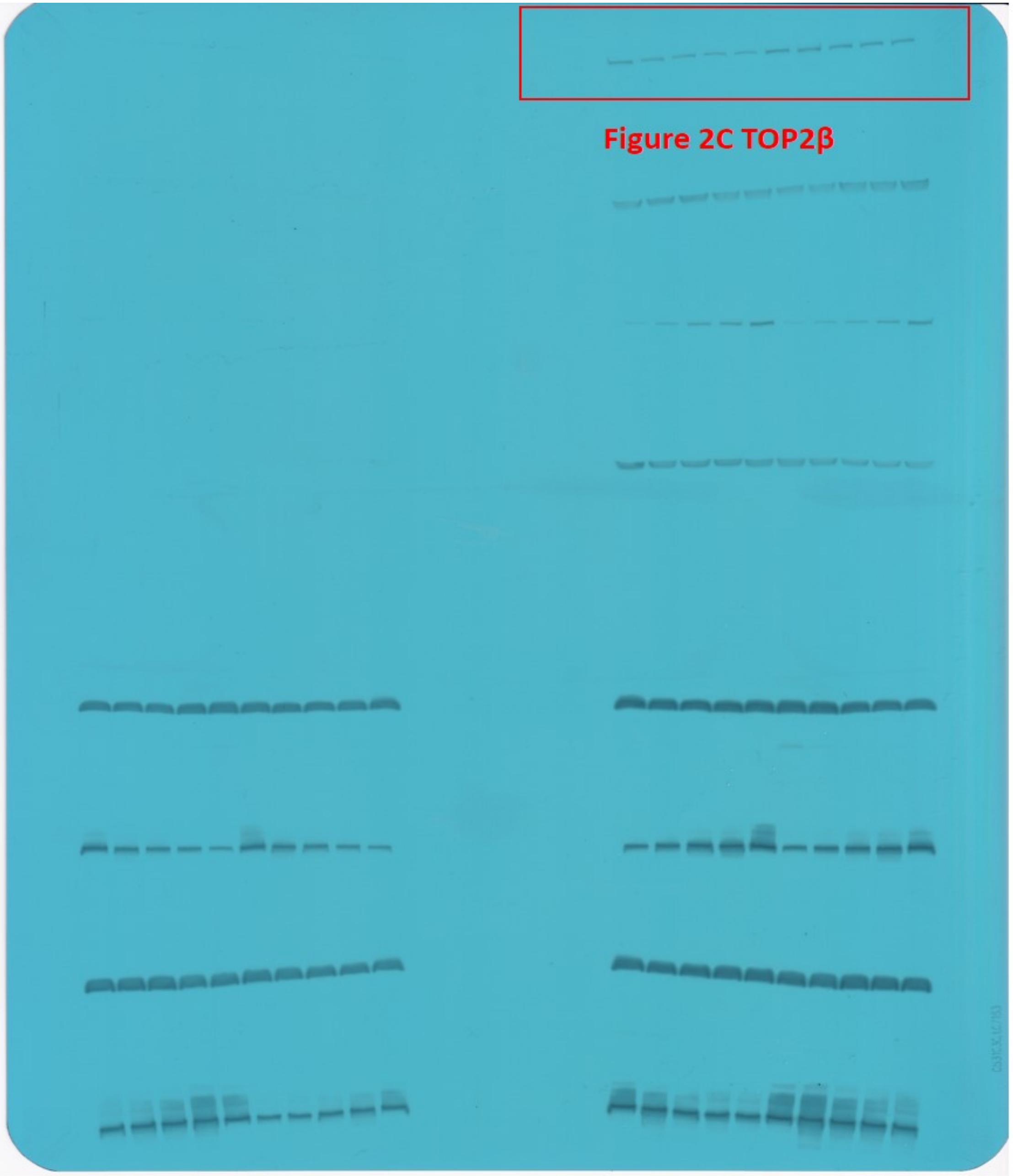

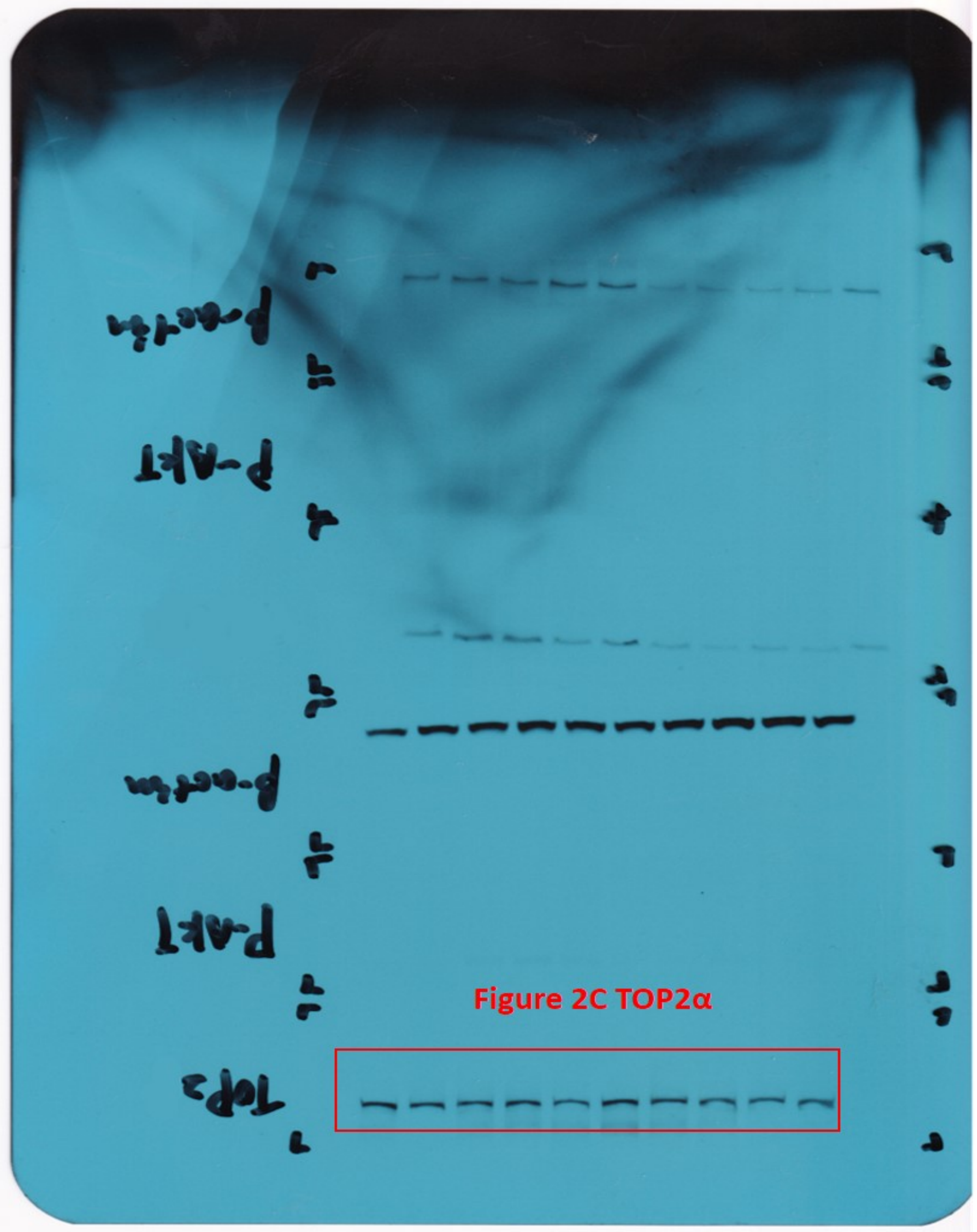

Supplementary Fig.2

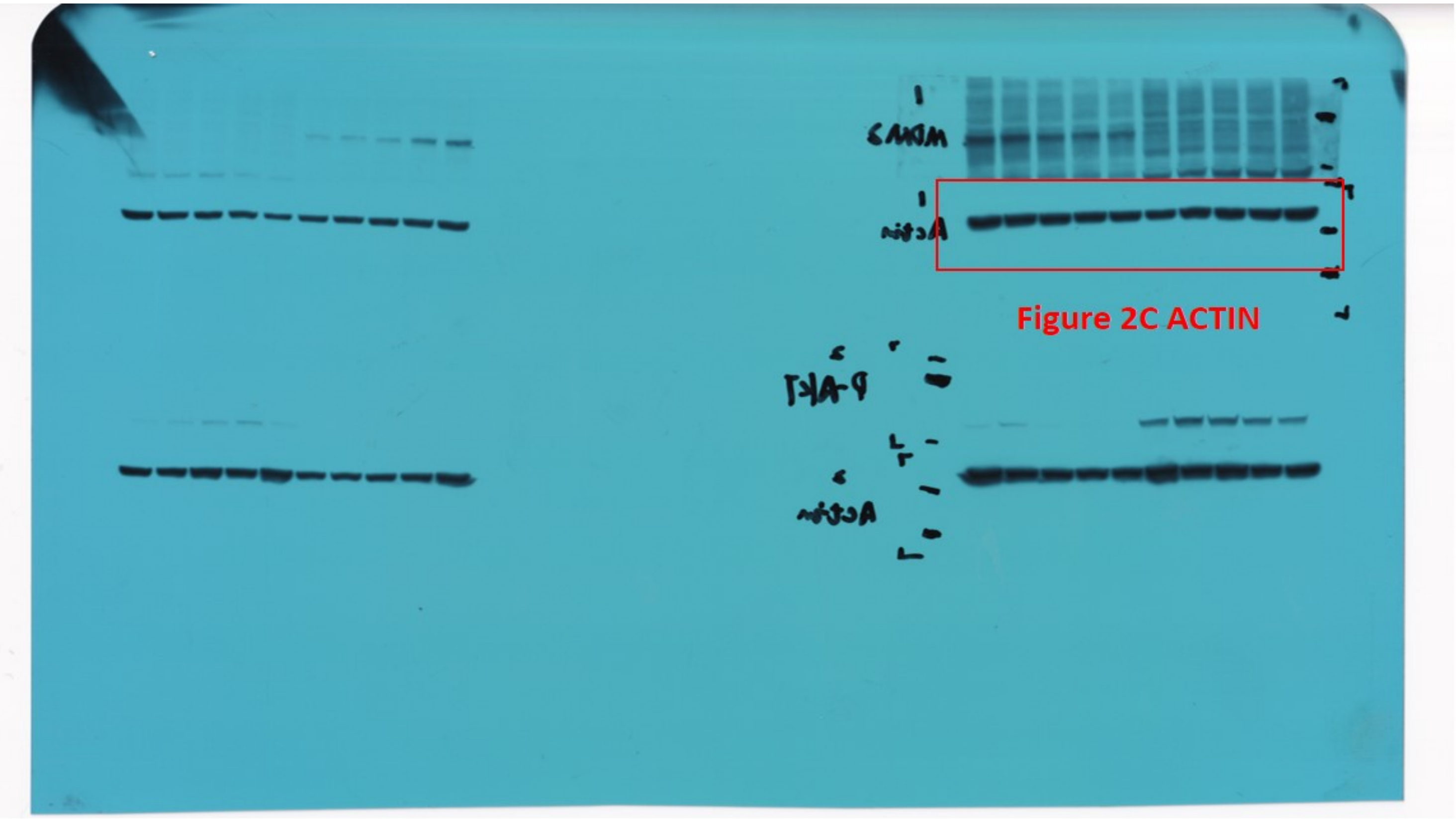

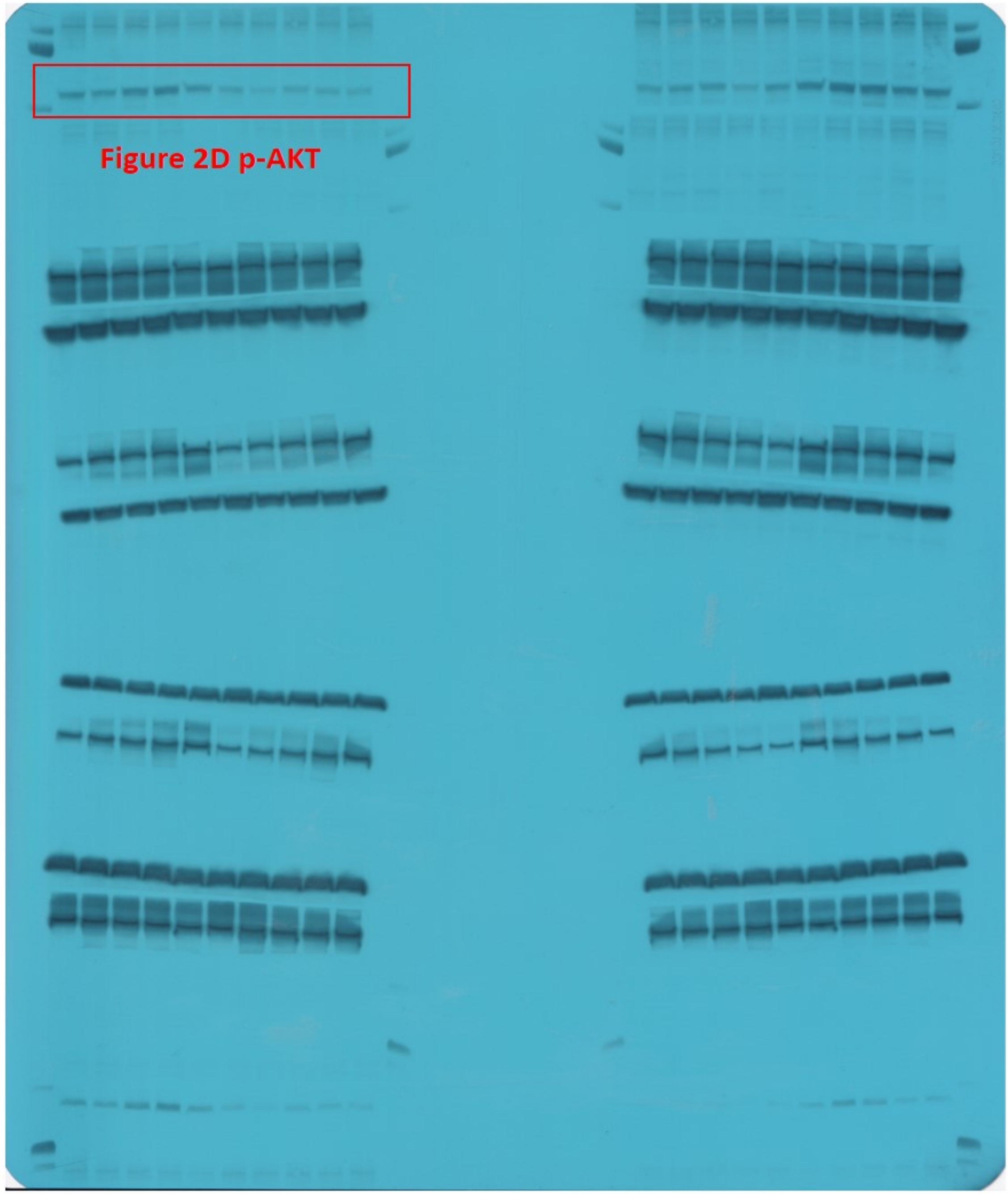

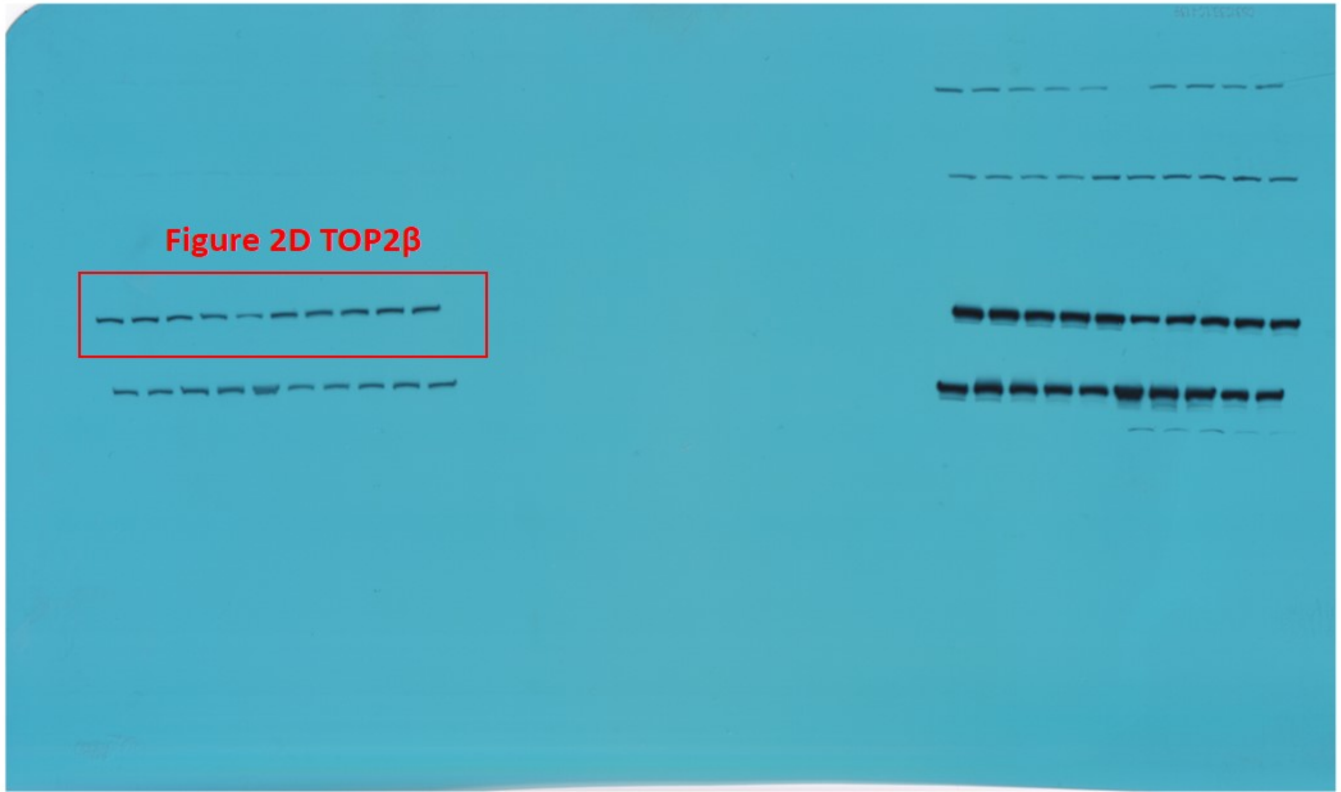

Supplementary Fig.2

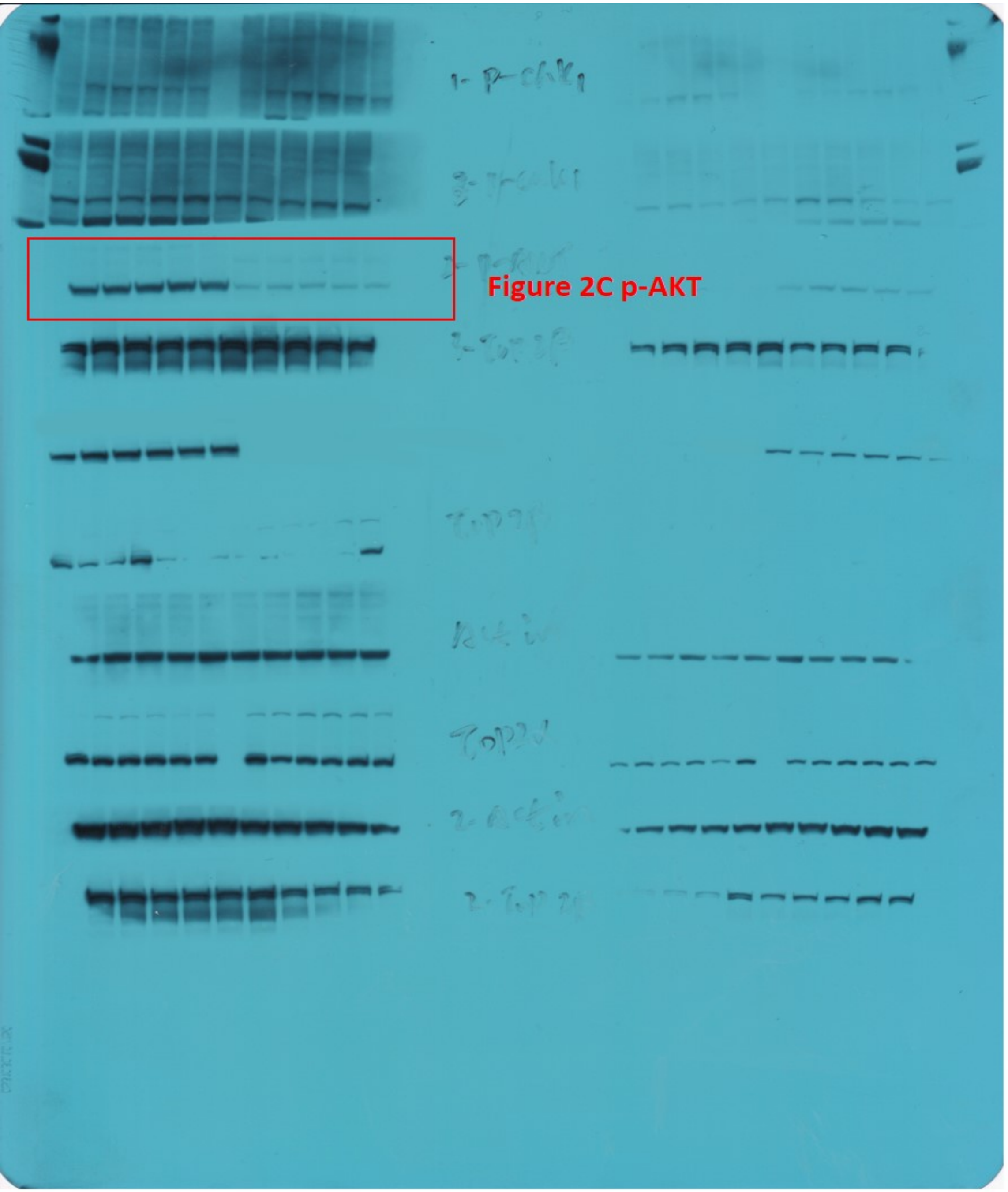

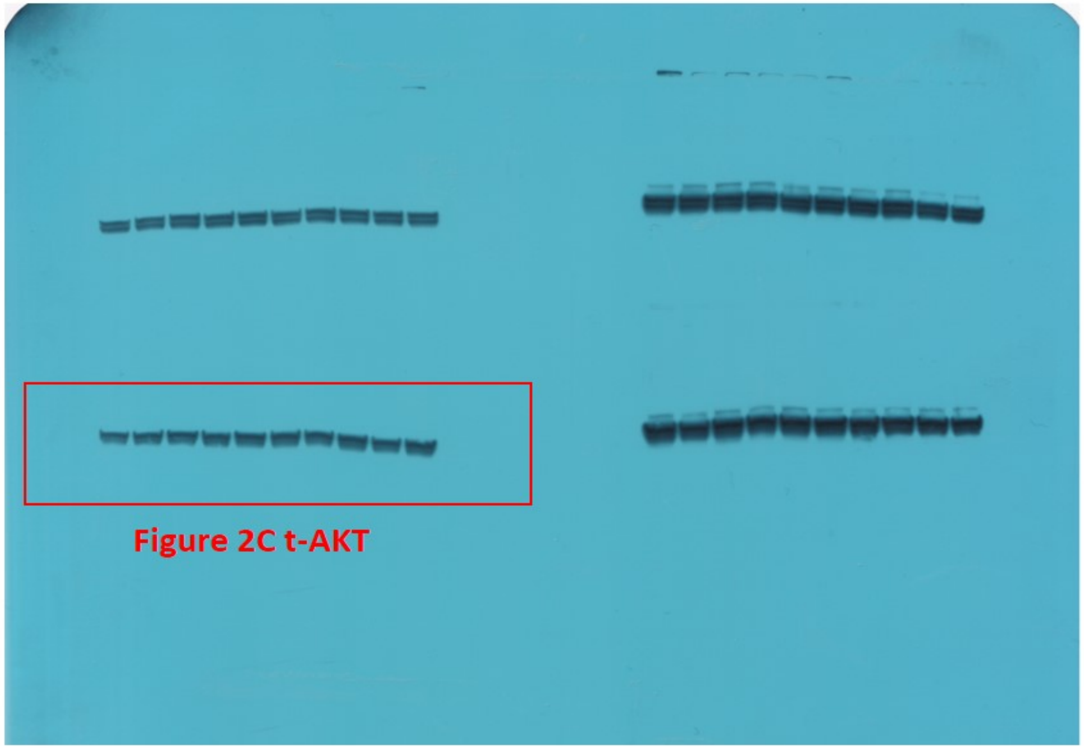

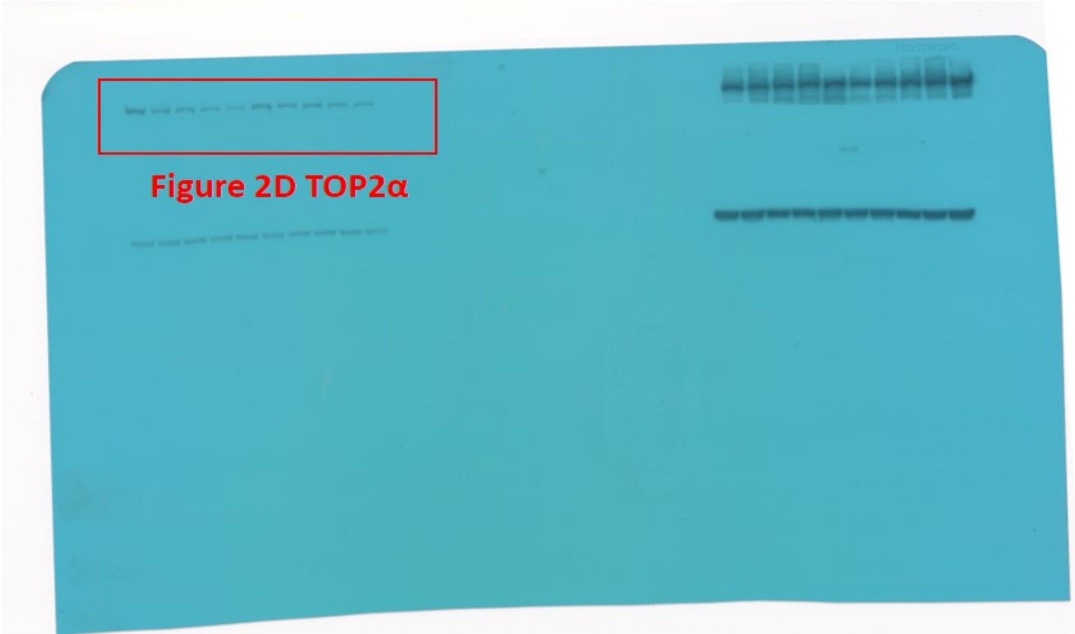

Supplementary Fig.2

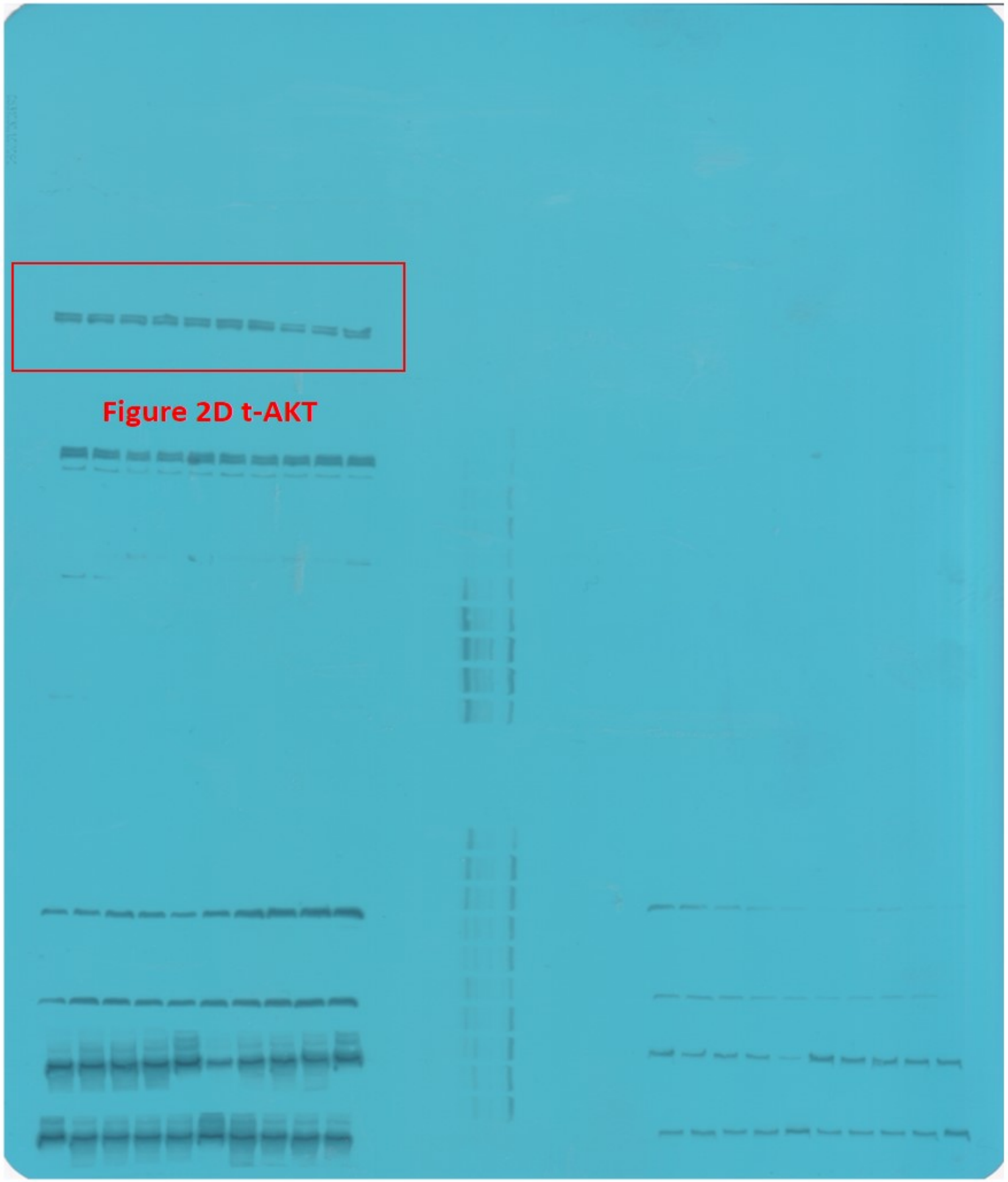

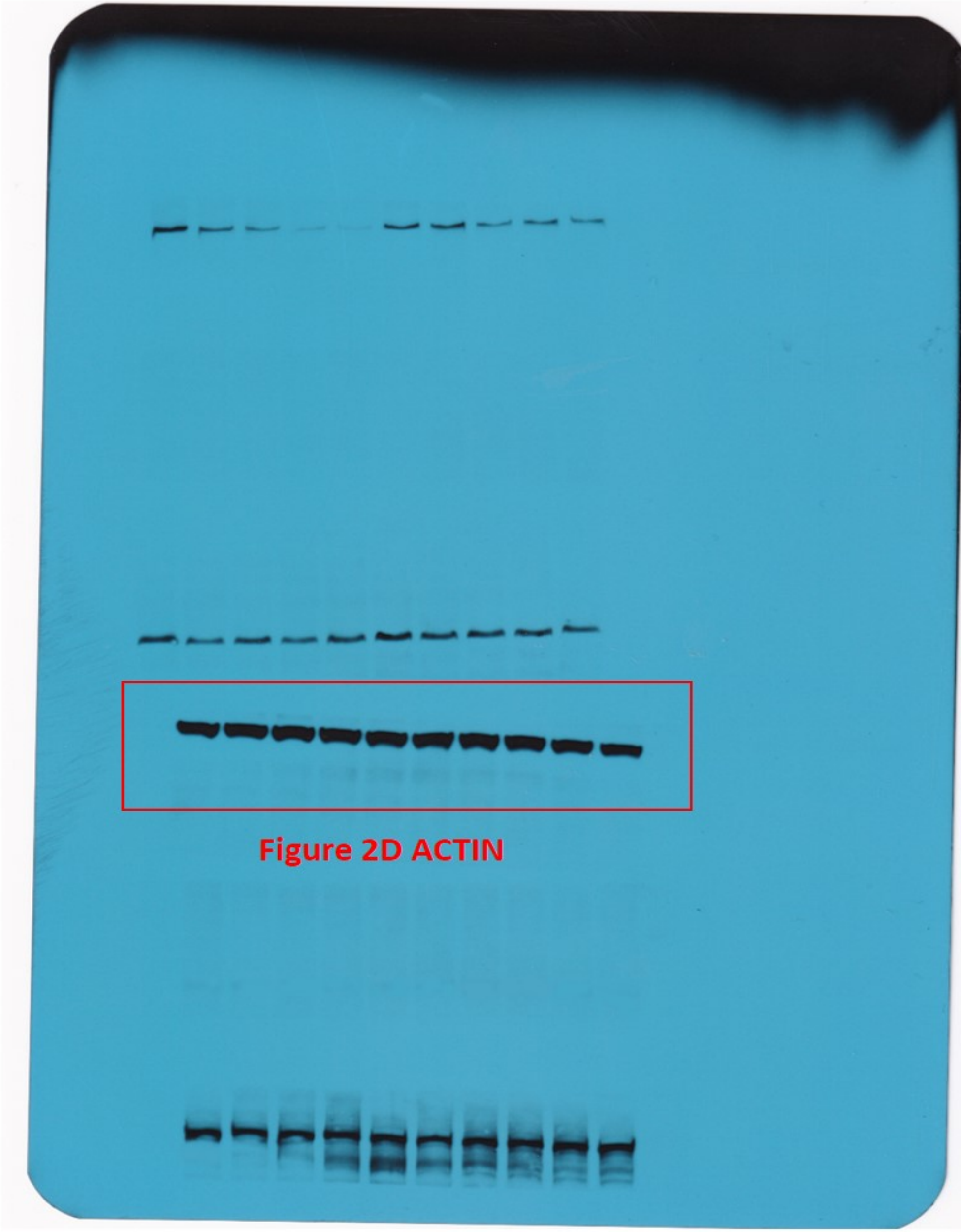

Supplementary Fig.2

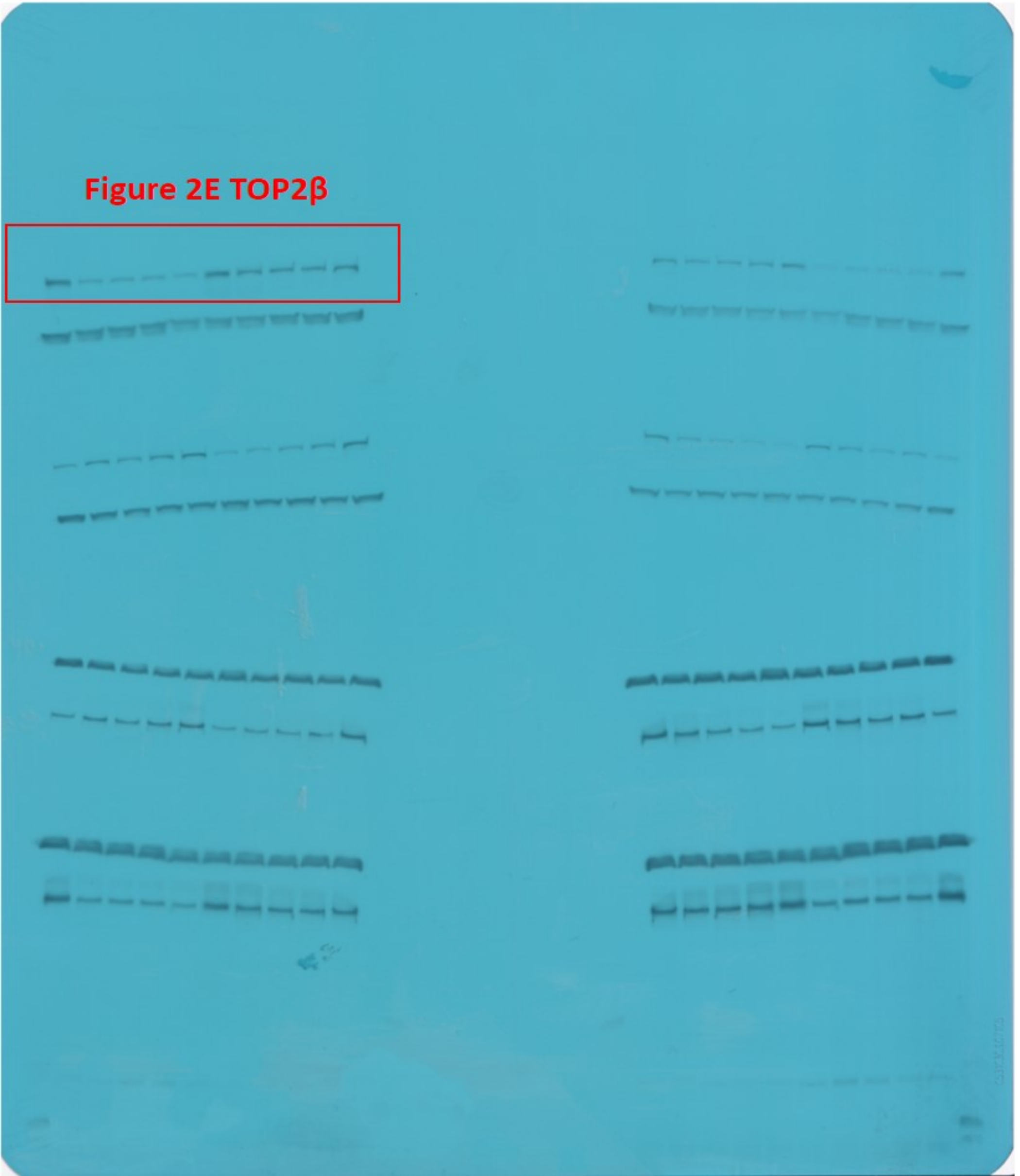

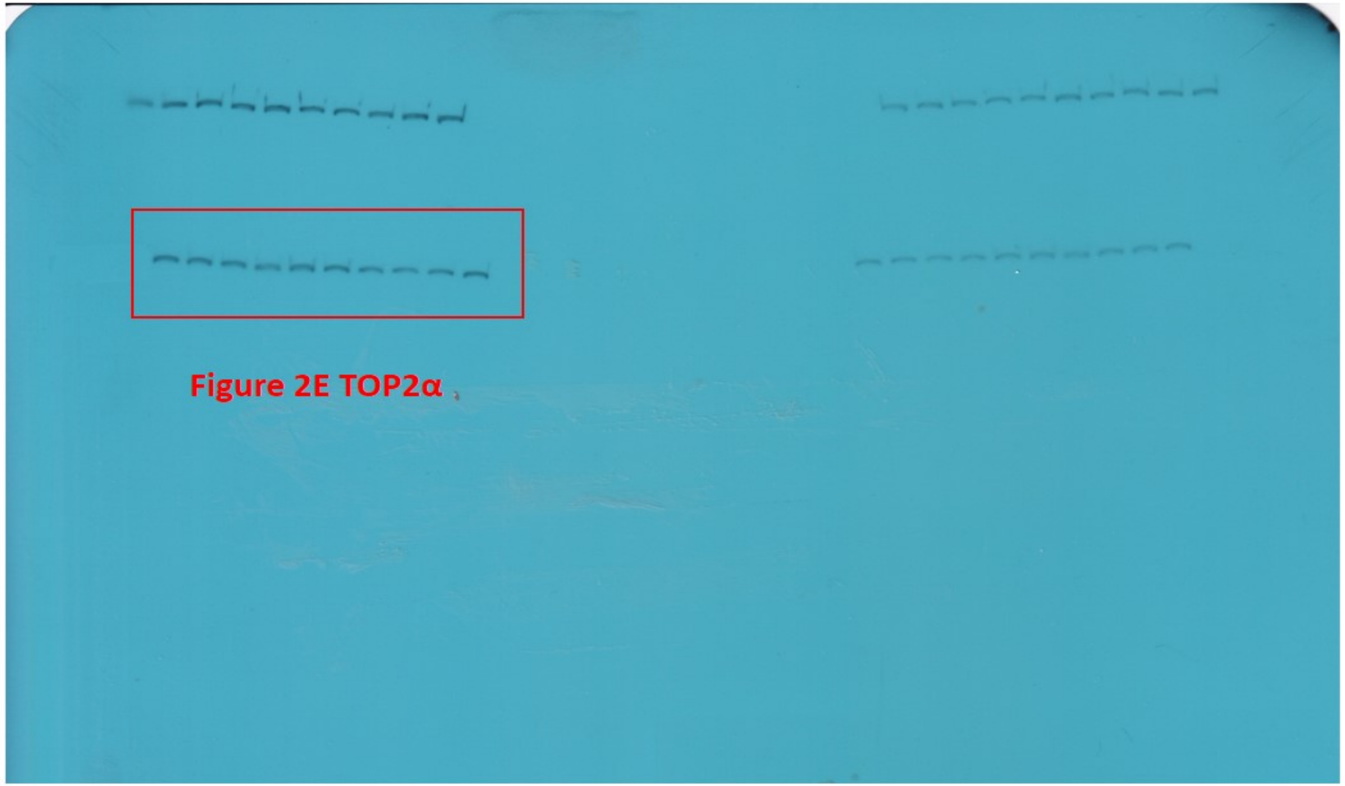

Supplementary Fig.2

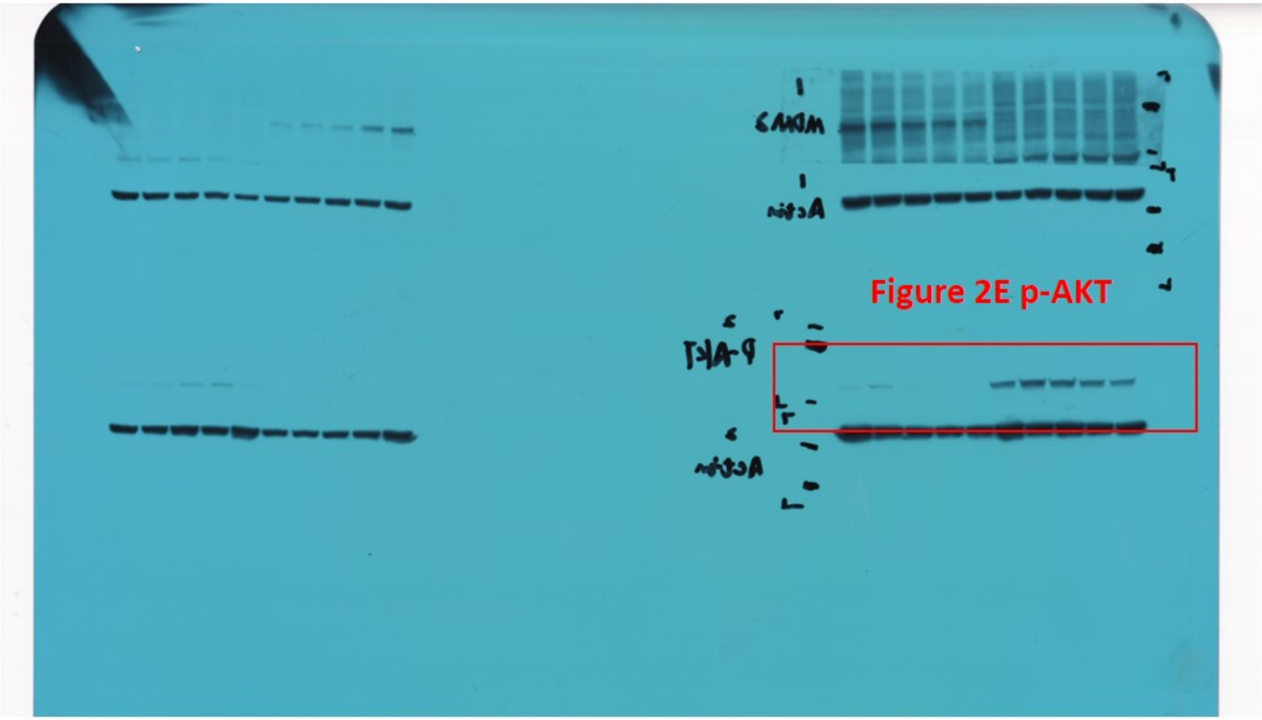

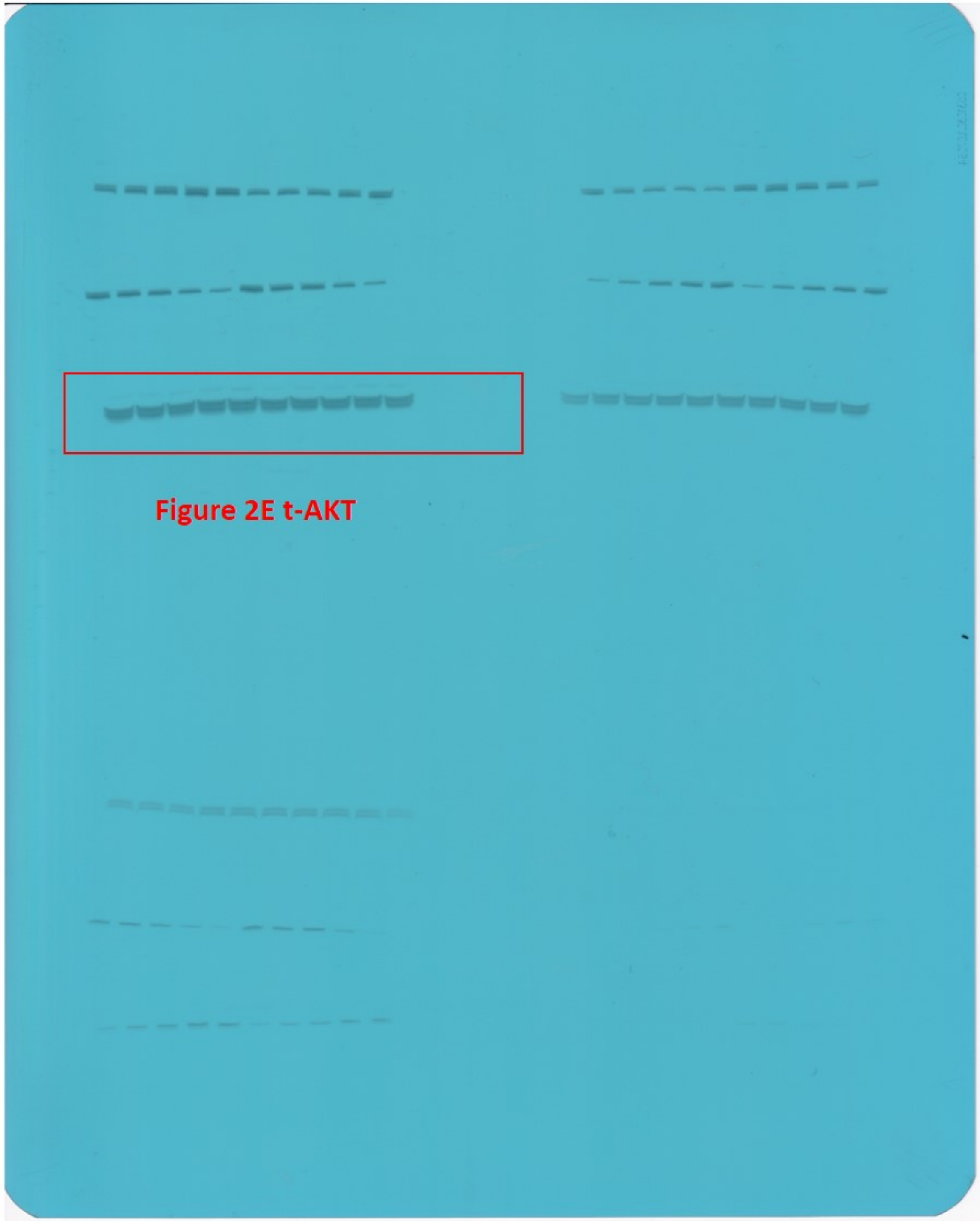

Supplementary Fig.2

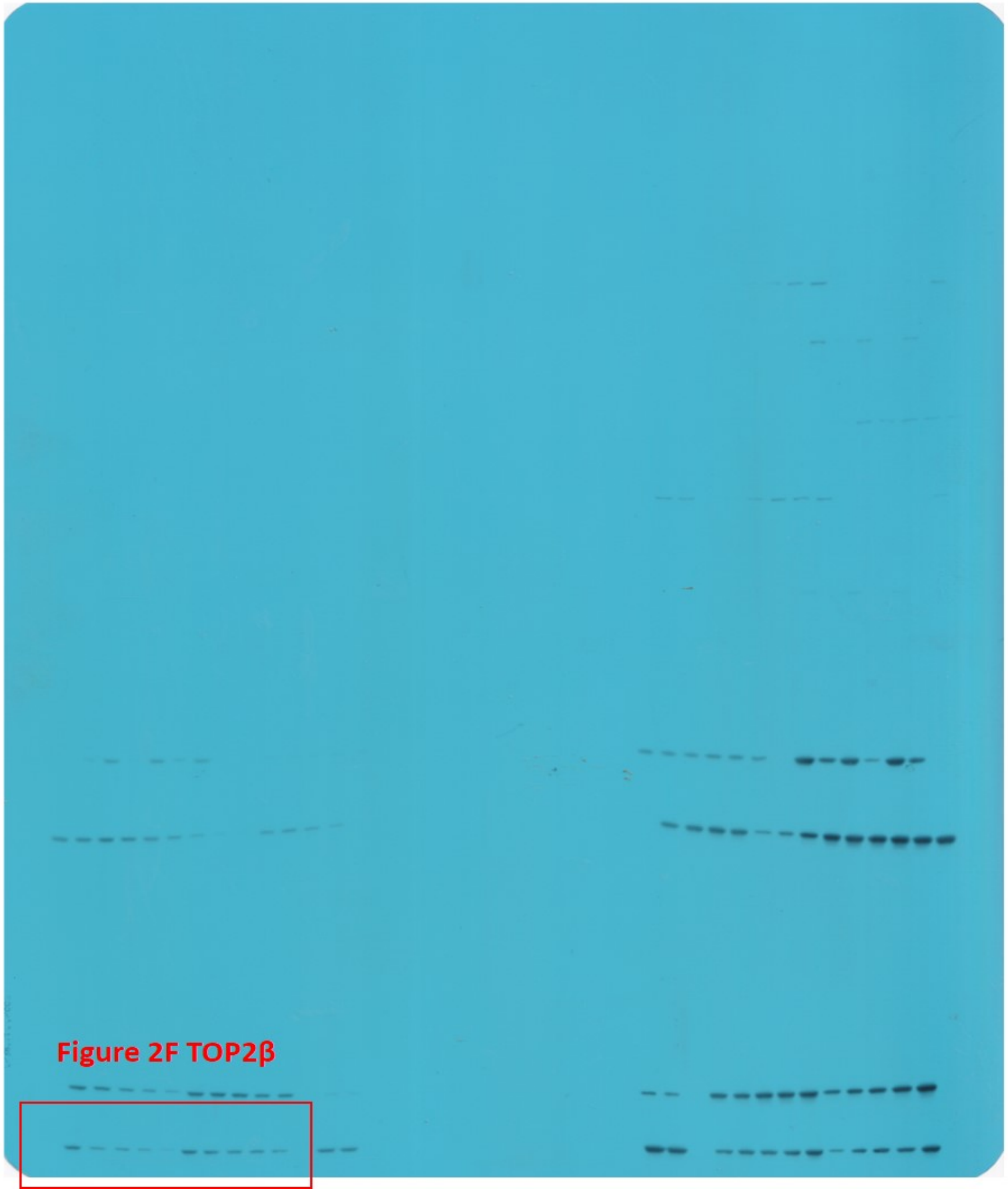

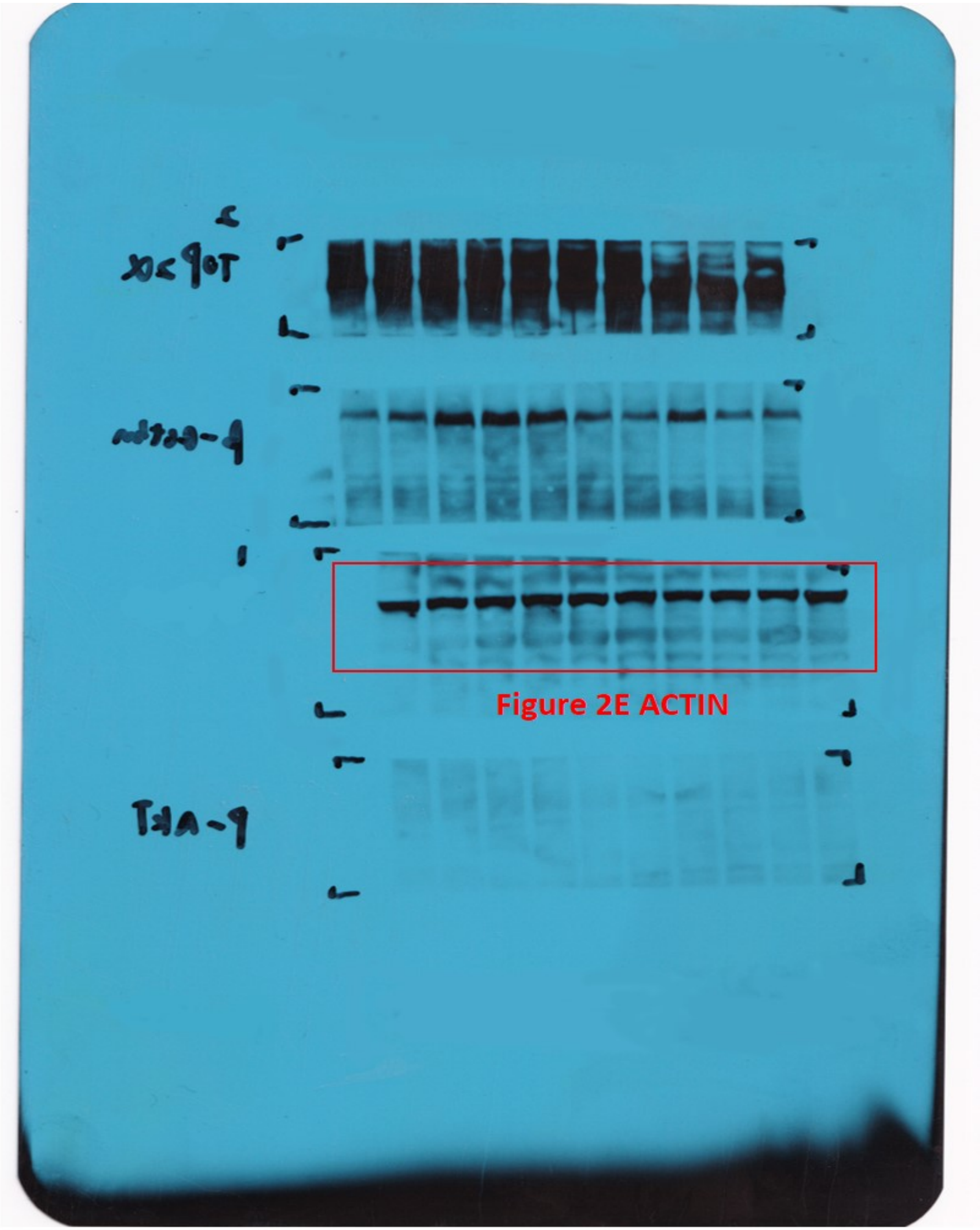

Supplementary Fig.2

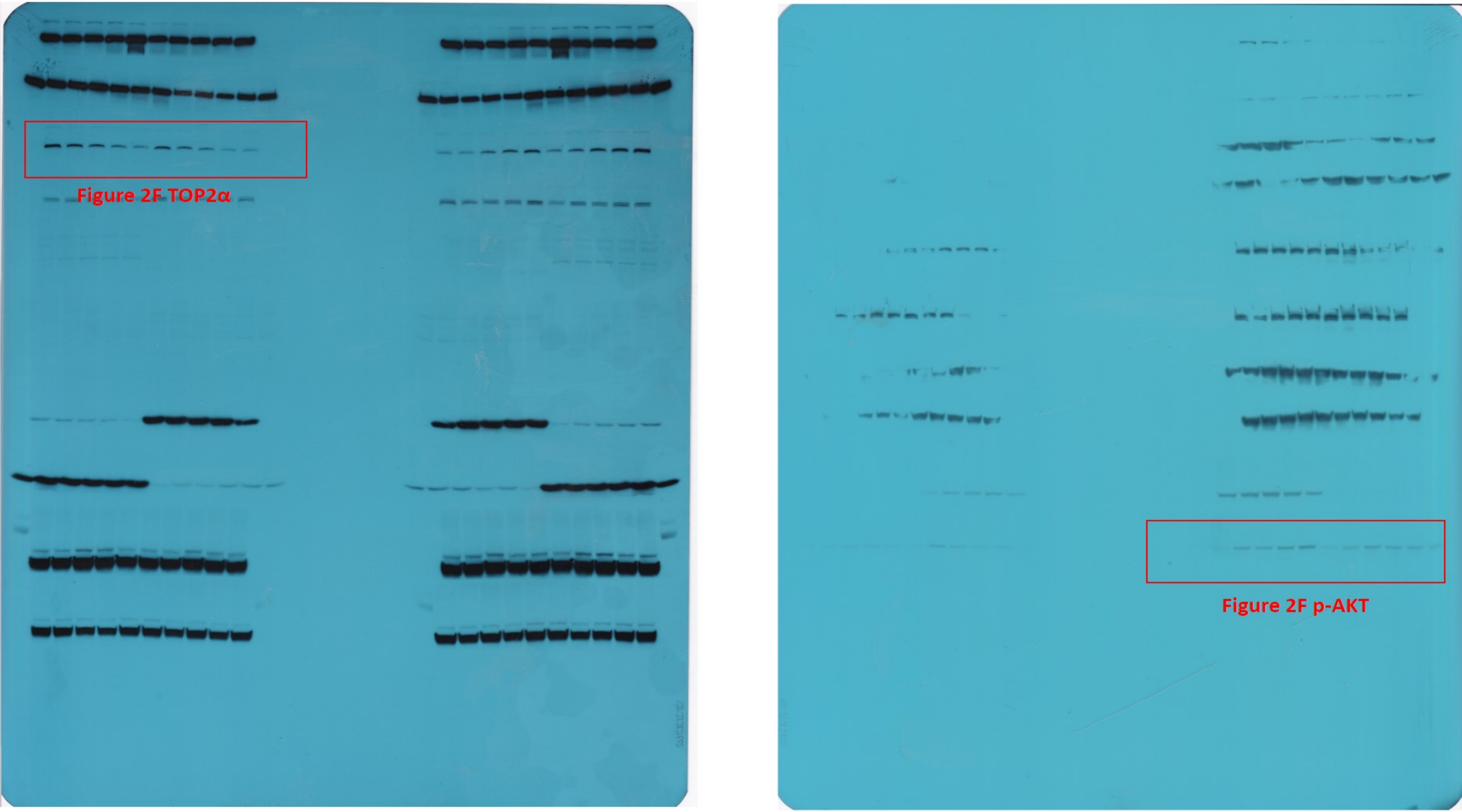

Supplementary Fig.2

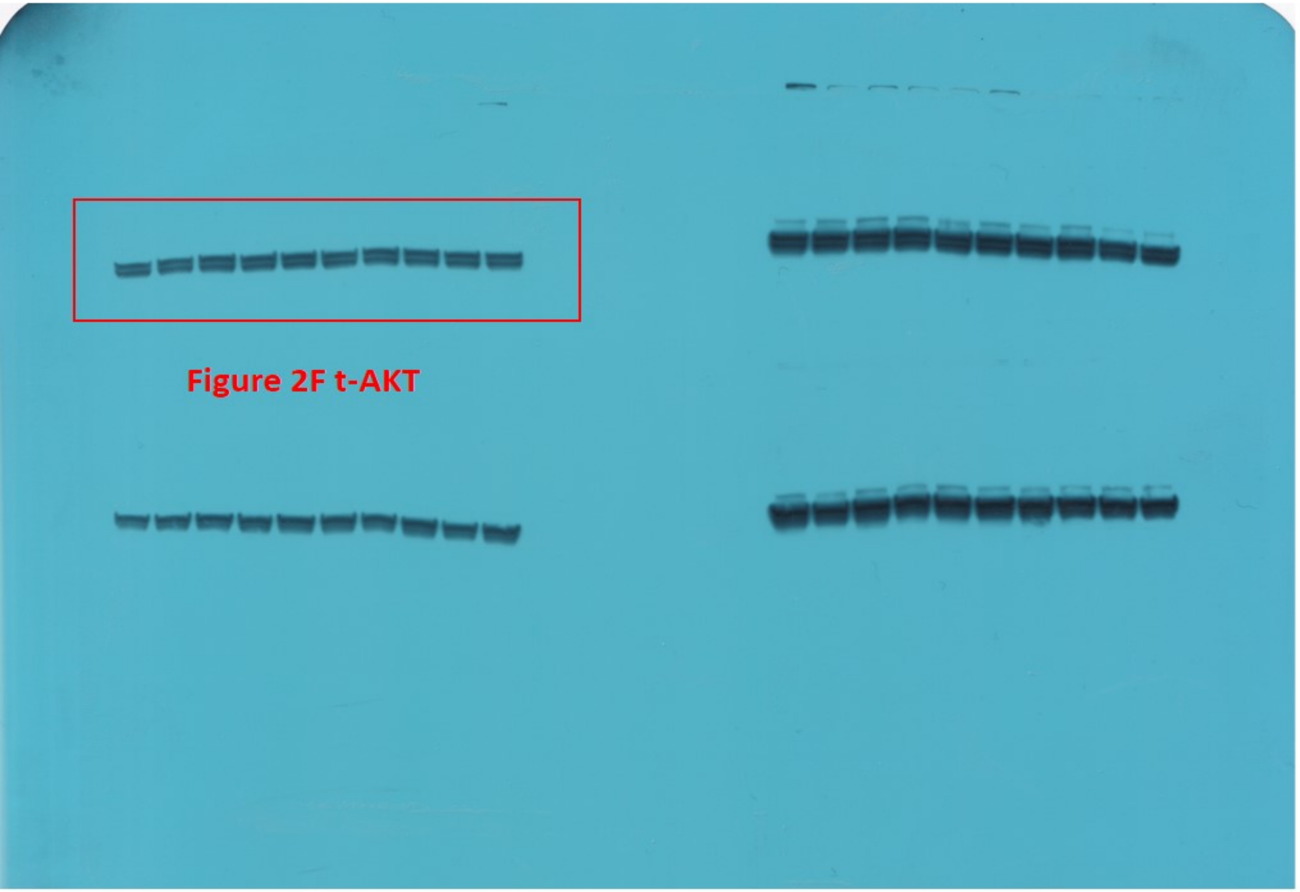

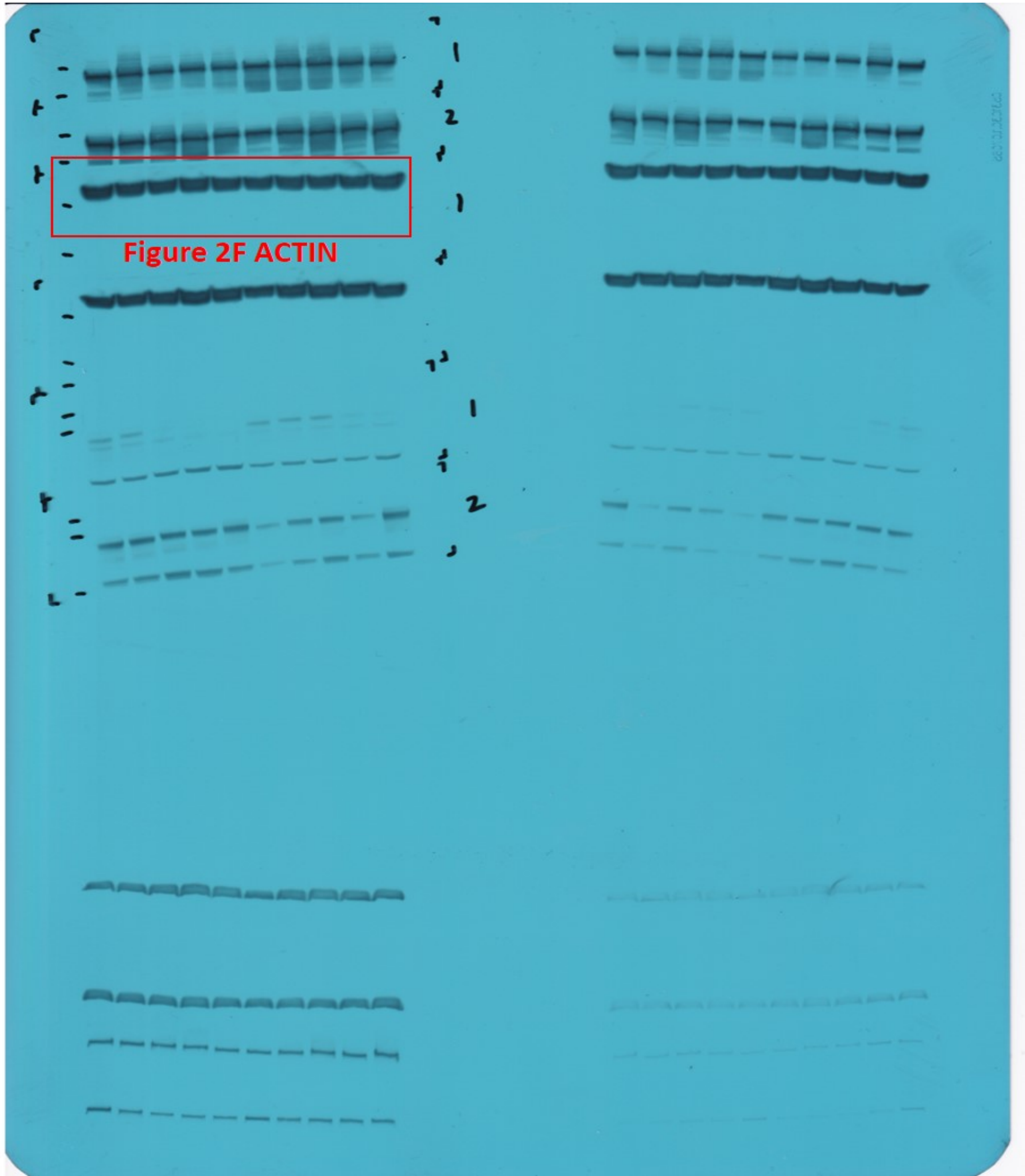

Supplement: Supplementary file 2 — Supplementary Information 2. [file 41598_2024_59332_MOESM2_ESM.pdf]
