# Supplementary material for: Author Correction: ATM inhibitor KU60019 synergistically sensitizes lung cancer cells to topoisomerase II poisons by multiple mechanisms
Source: Sci Rep. 2024 Apr 16;14:8785. doi: 10.1038/s41598-024-59332-9 (PMC11021496; doi:10.1038/s41598-024-59332-9)

Supplementary Fig.5

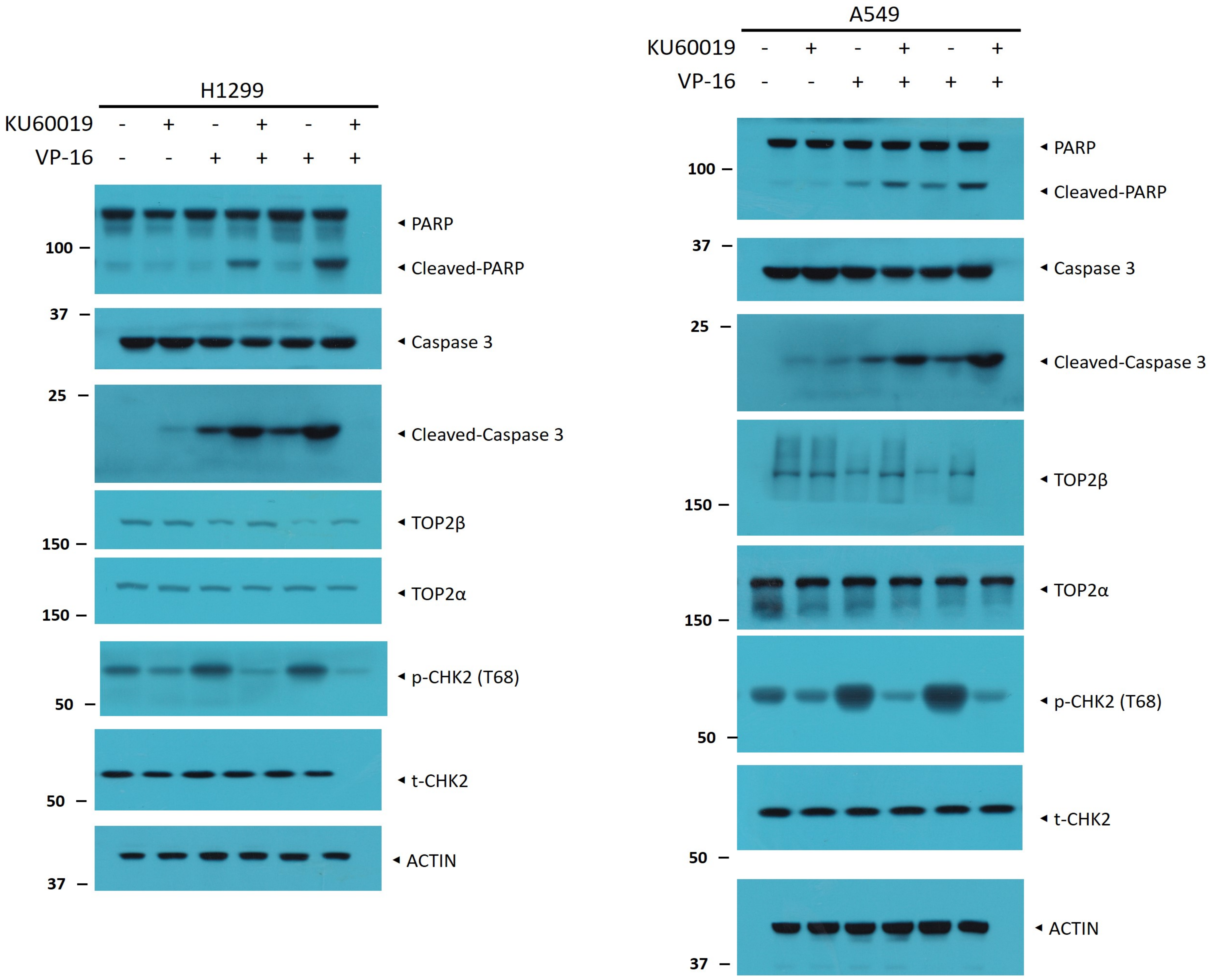

Supplementary Fig.5

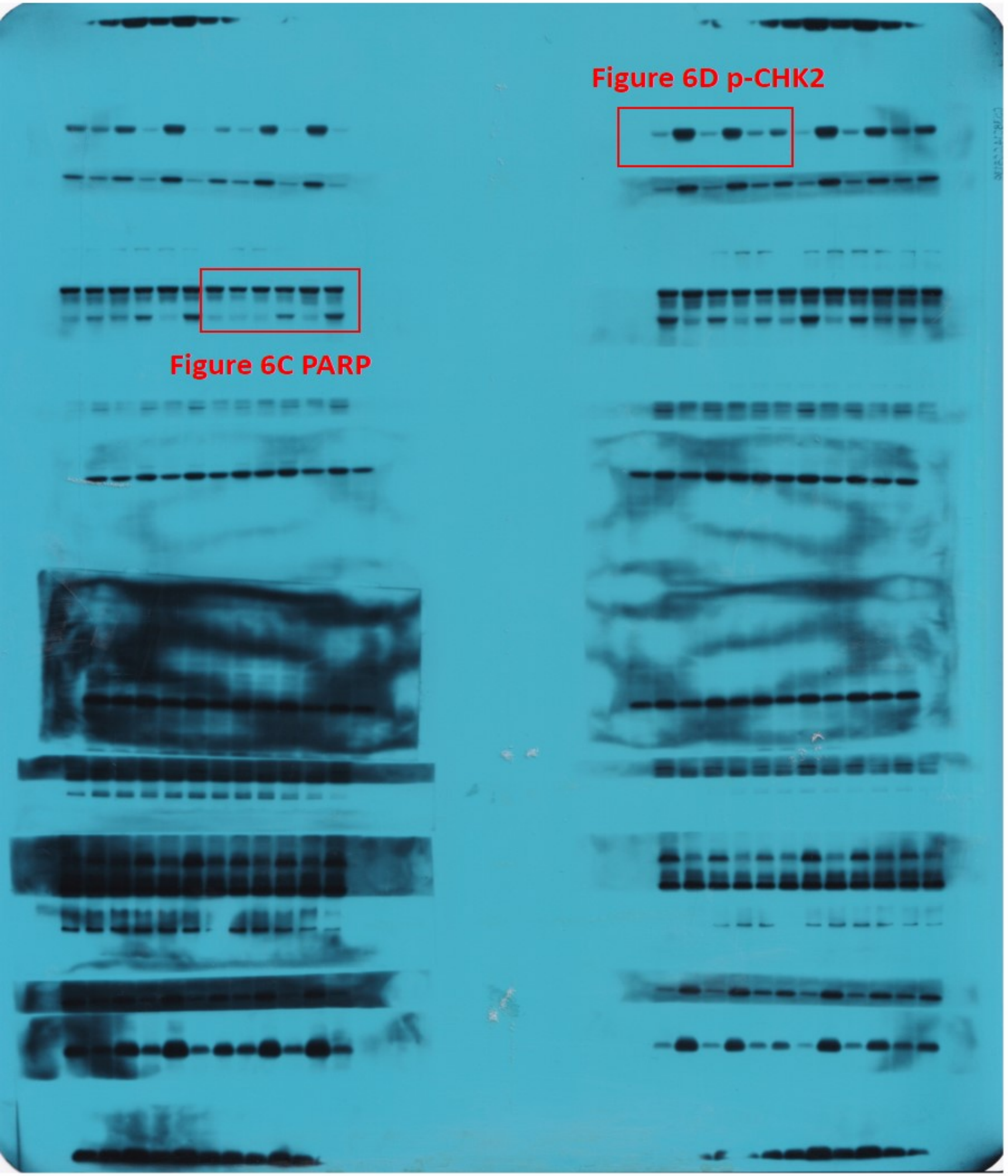

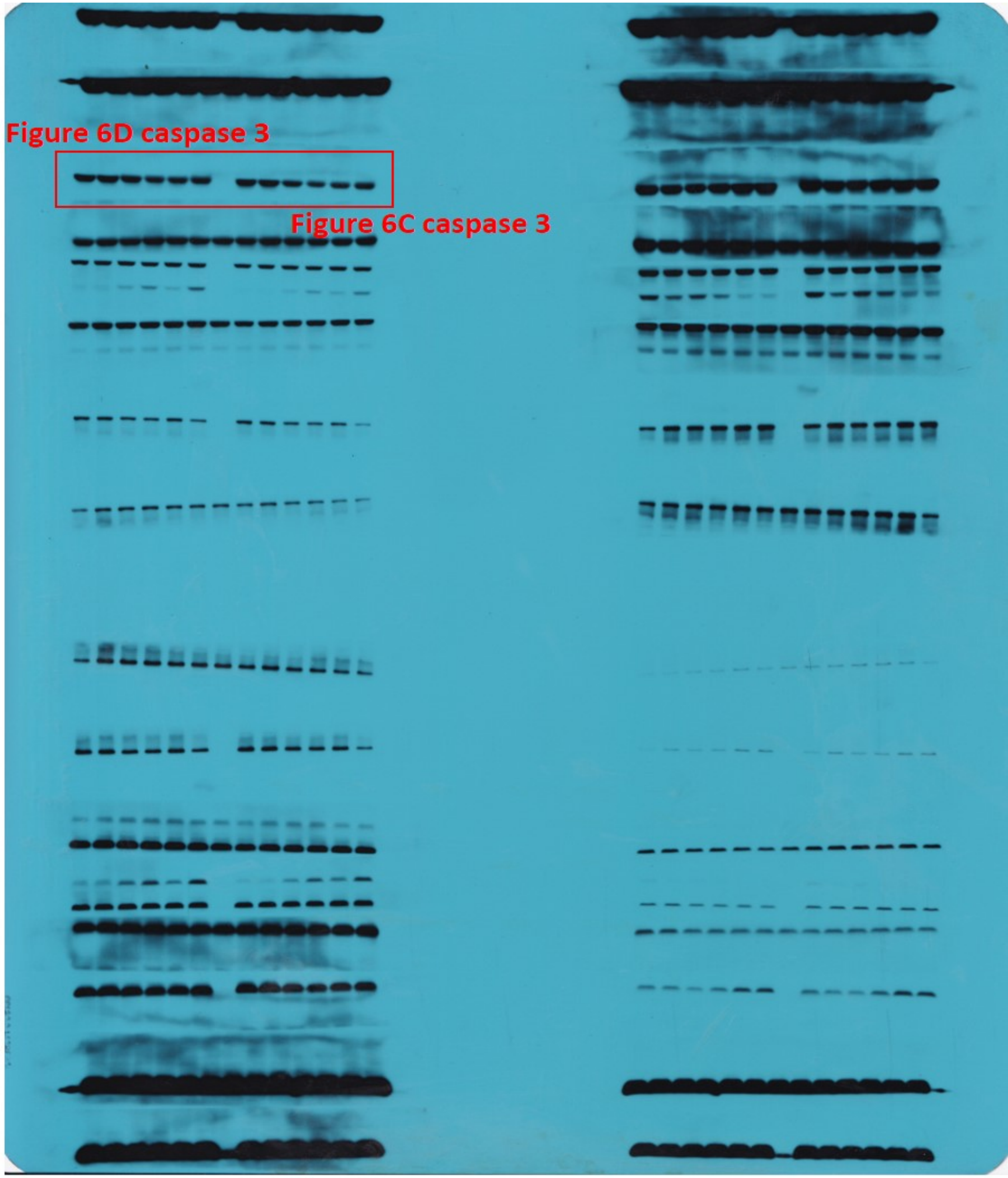

Supplementary Fig.5

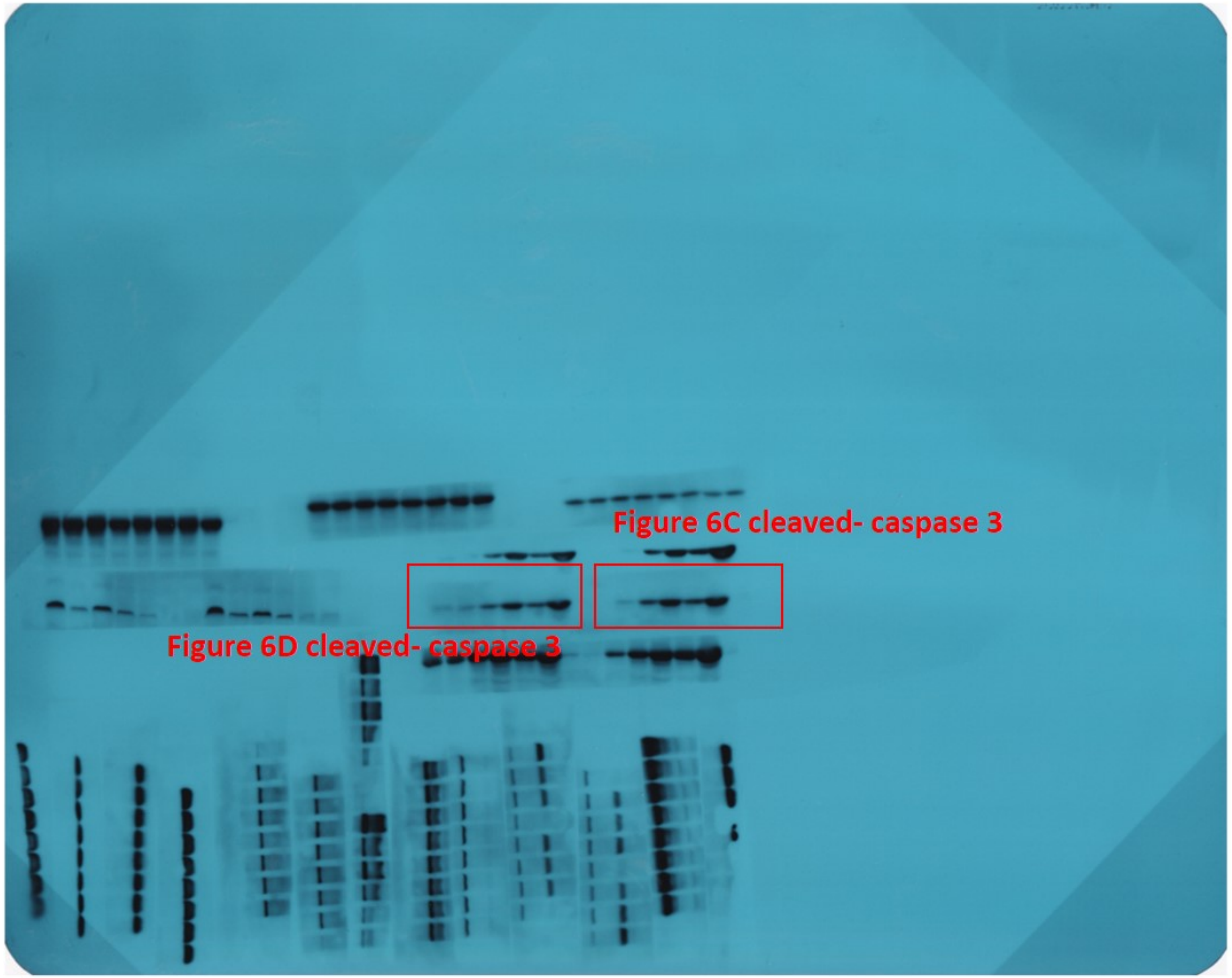

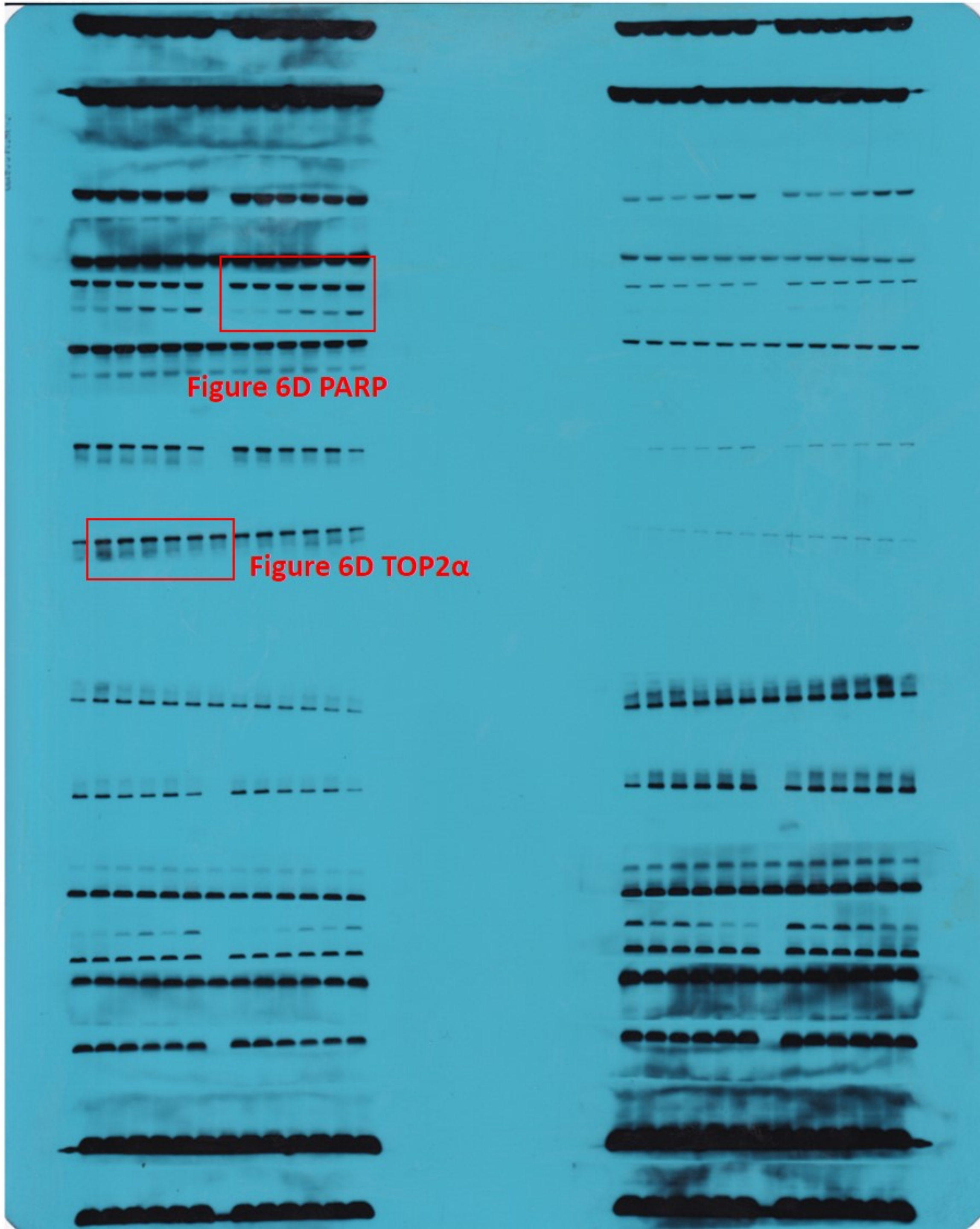

Supplementary Fig.5

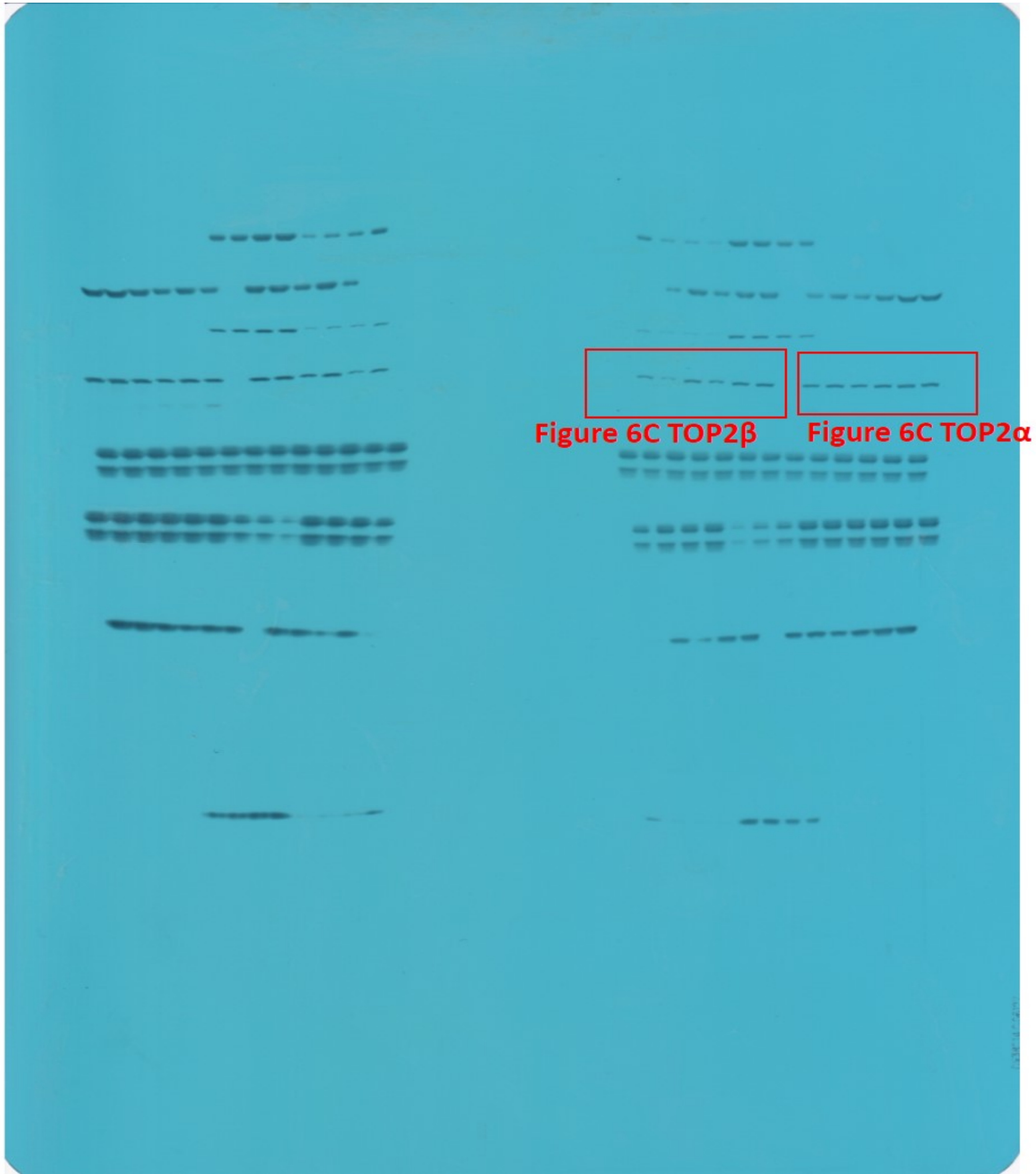

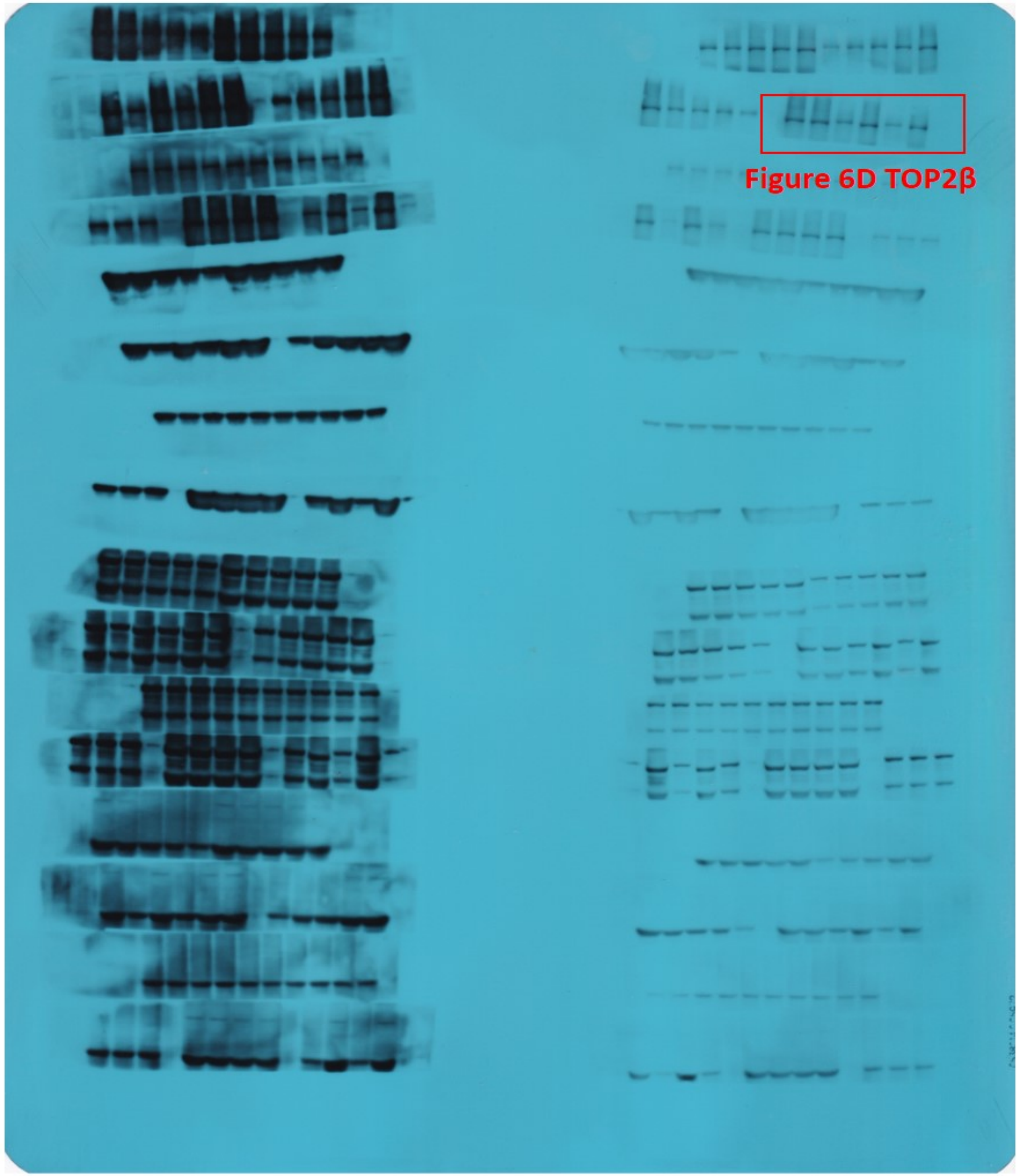

Supplementary Fig.5

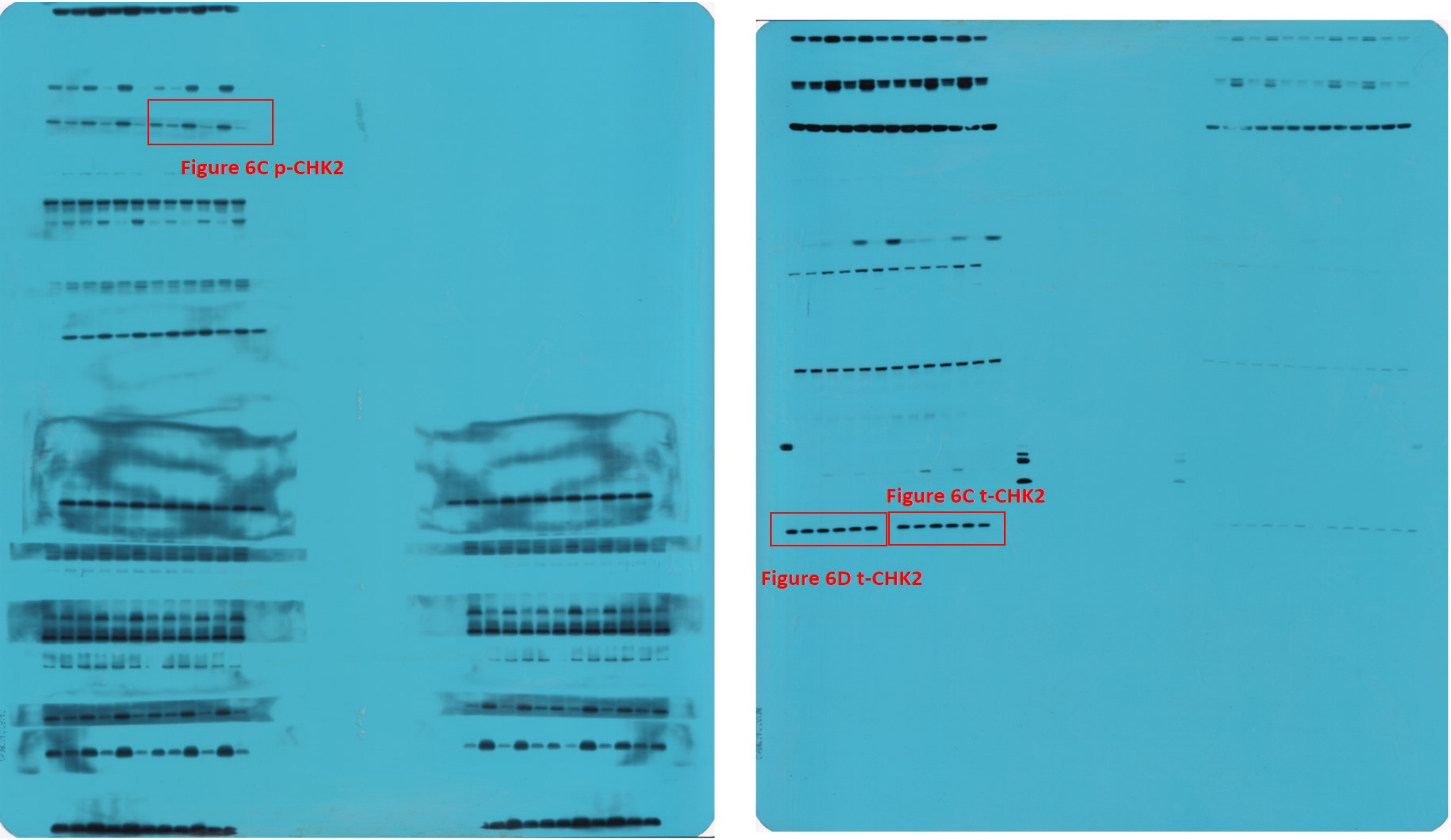

Supplementary Fig.5

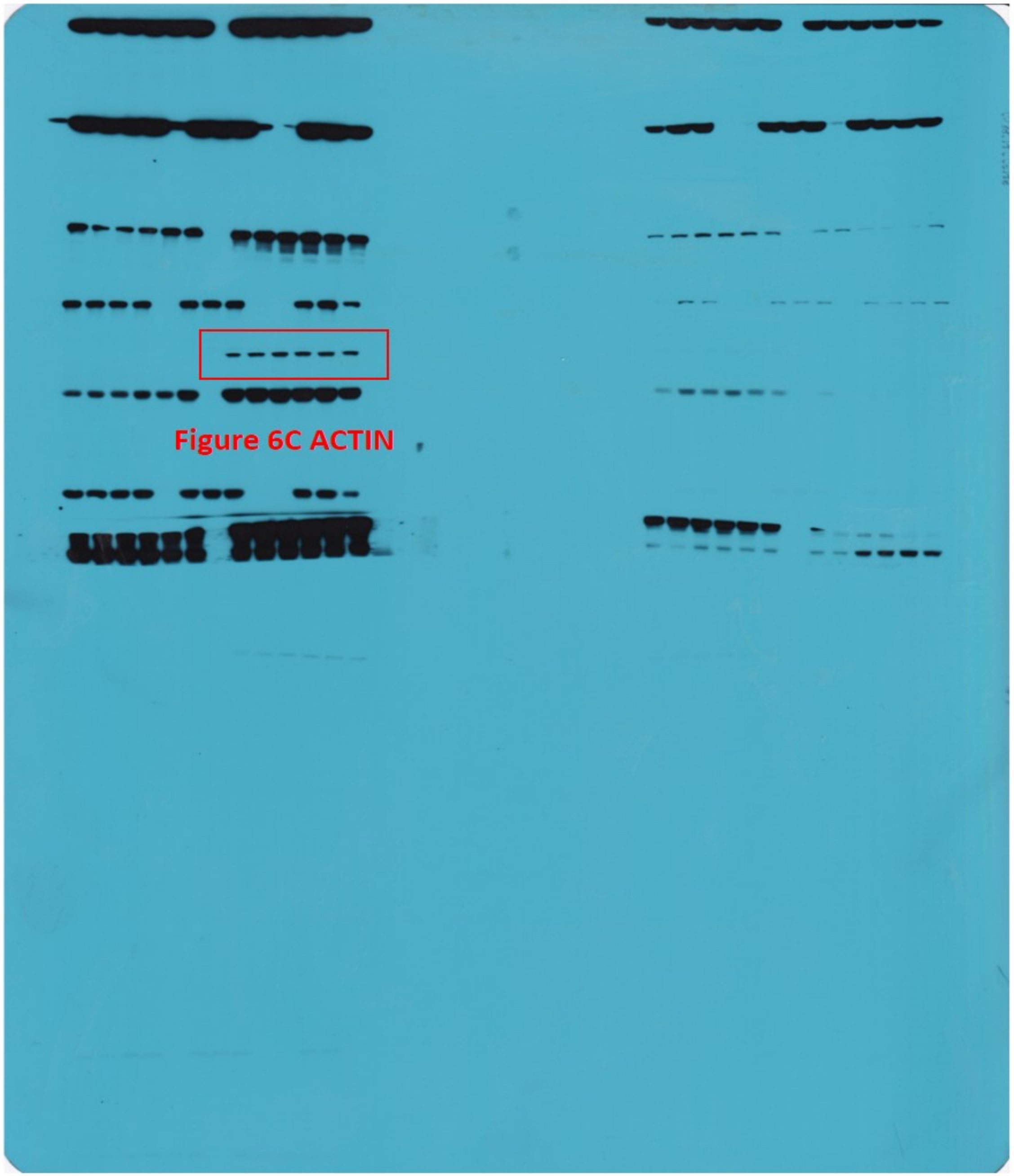

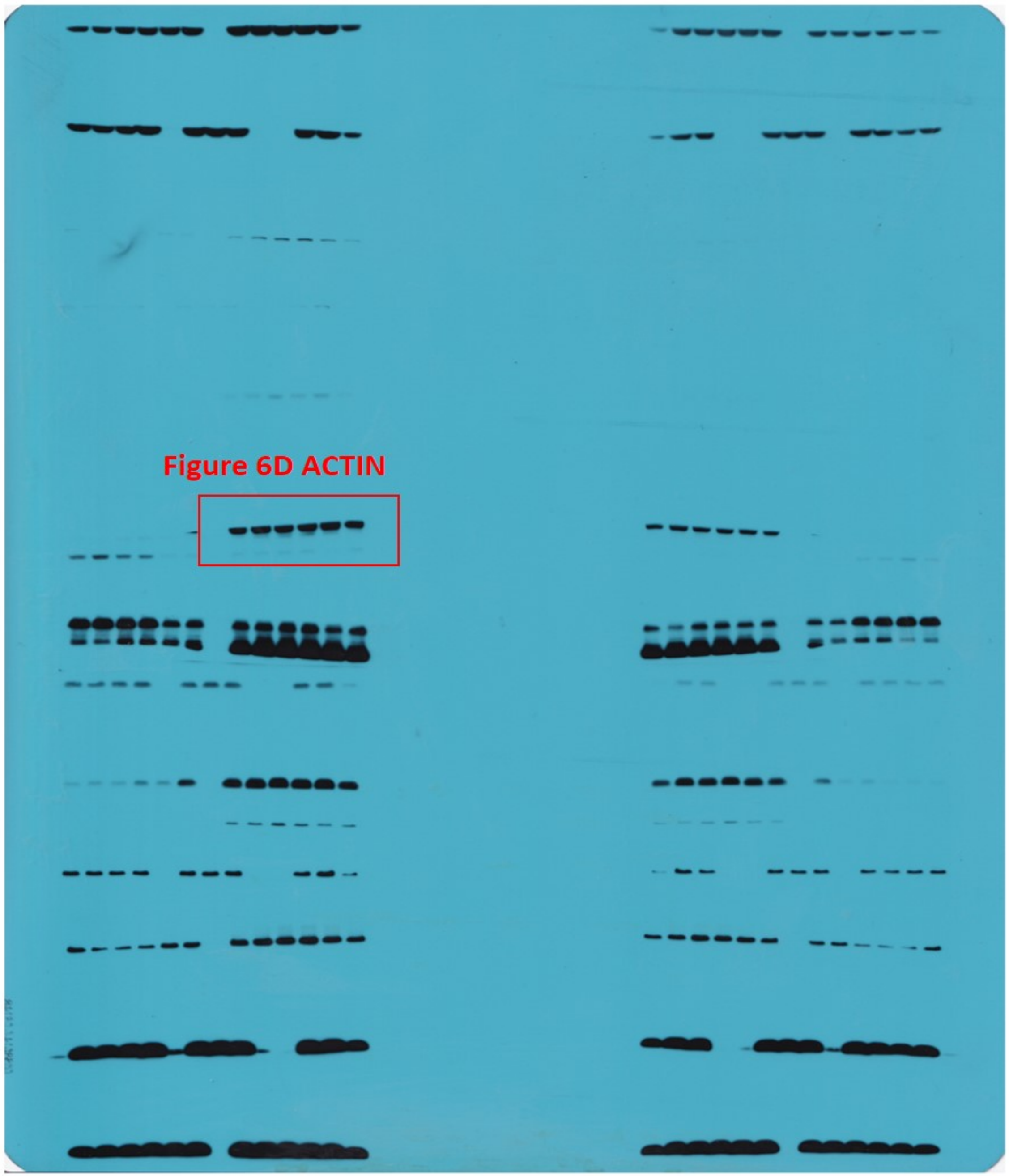

Supplement: Supplementary file 5 — Supplementary Information 5. [file 41598_2024_59332_MOESM5_ESM.pdf]
